# Supplementary material for: Intact Transition Epitope Mapping—Force Differences between Original and Unusual Residues (ITEM-FOUR)
Source: Biomolecules. 2023 Jan 16;13(1):187. doi: 10.3390/biom13010187 (PMC9856199; doi:10.3390/biom13010187)
Supplement: Supplementary file 1 [file biomolecules-13-00187-s001.zip › biomolecules-2113799-supplementary.pdf]

biomolecules

MDPI

Supplement

Intact Transition Epitope Mapping–Force differences between Original and Unusual Residues (ITEM-FOUR)

Claudia Röwer [1], Christian Ortmann [2], Andrei Neamtu [3], Reham F. El-Kased [4], and Michael O. Glocker [1,*]

[1] Proteome Center Rostock, University Medicine Rostock and University of Rostock, Schillingallee 69, 18059 Rostock, Germany; claudia.roewer@uni-rostock.de
[2] Waters GmbH | TA Instruments, Helfmann-Park 10, 65760 Eschborn, Germany; christian_ortmann@waters.com
[3] TRANSCEND Centre – Regional Institute of Oncology (IRO) Iasi, Str. General Henri Mathias Berthelot, Nr. 2–4, 700483 Iași, România; andrei.neamtu@umfiasi.ro
[4] Department of Microbiology & Immunology, Faculty of Pharmacy, The British University in Egypt, Suez Rd, 11837 EL Sherouk City, Egypt
* Correspondence: reham.kased@bue.edu.eg (R.F.E.-K.); michael.glocker@uni-rostock.de (M.O.G.); Tel.: +49-381-494-4930 (M.O.G.)

Abstract: Antibody-based point-of-care diagnostics have become indispensable for modern medicine. In-depth analysis of antibody recognition mechanisms is the key to tailoring the accuracy and precision of test results, which themselves are crucial for targeted and personalized therapy. A rapid and robust method is desired by which binding strengths between antigens and antibodies of concern can be fine-mapped with amino acid residue resolution to examine the assumedly serious effects of single amino acid polymorphisms on insufficiencies of antibody-based detection capabilities of, e.g., life-threatening conditions such as myocardial infarction. The experimental ITEM-FOUR approach makes use of modern mass spectrometry instrumentation to investigate intact immune complexes in the gas phase. ITEM-FOUR together with molecular dynamics simulations, enables the determination of the influences of individually exchanged amino acid residues within a defined epitope on an immune complex's binding strength. Wild-type and mutated epitope peptides were ranked according to their experimentally determined dissociation enthalpies relative to each other, thereby revealing which single amino acid polymorphism caused weakened, impaired, and even abolished antibody binding. Investigating a diagnostically relevant human cardiac Troponin I epitope for which seven nonsynonymous single nucleotide polymorphisms are known to exist in the human population tackles a medically relevant but hitherto unsolved problem of current antibody-based point-of-care diagnostics.

Keywords: ITEM-FOUR; nanoESI mass spectrometry; immune complex analysis; personalized genomics; single amino acid polymorphism

Röwer et al. Supplemental Figures

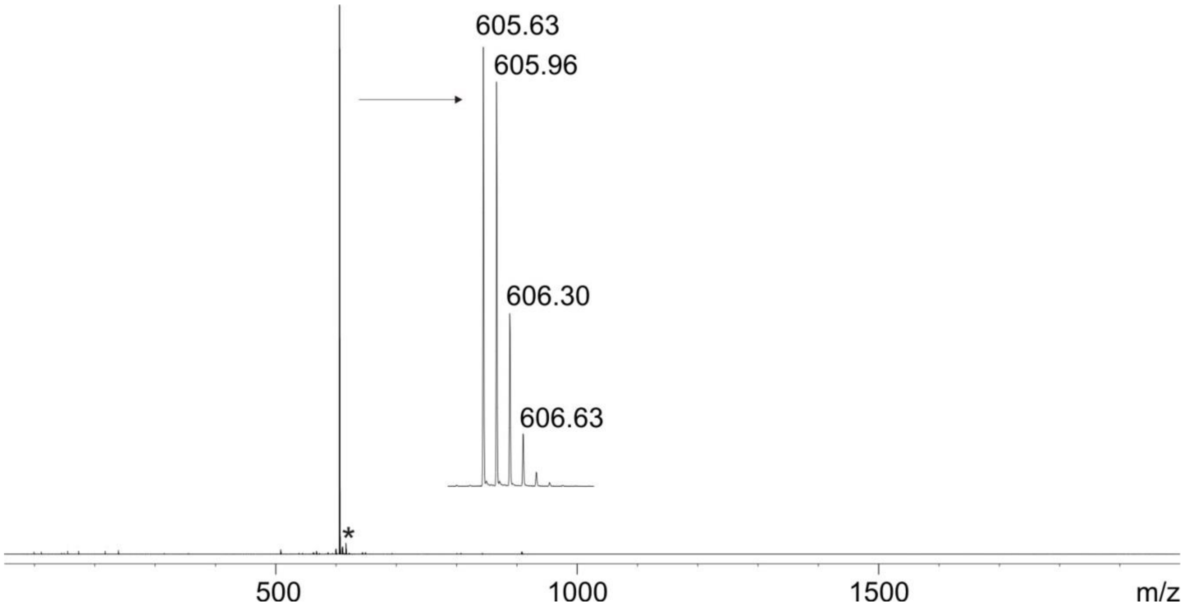

Figure S1. Nano-ESI mass spectrum of Troponin I peptide 1 (ENREVGDWRKNIDAL). The m/z values of the isotopically resolved triply charged peptide ion signal are given. *: sodium adduct. Solvent: 2% acetic acid, 10% methanol.

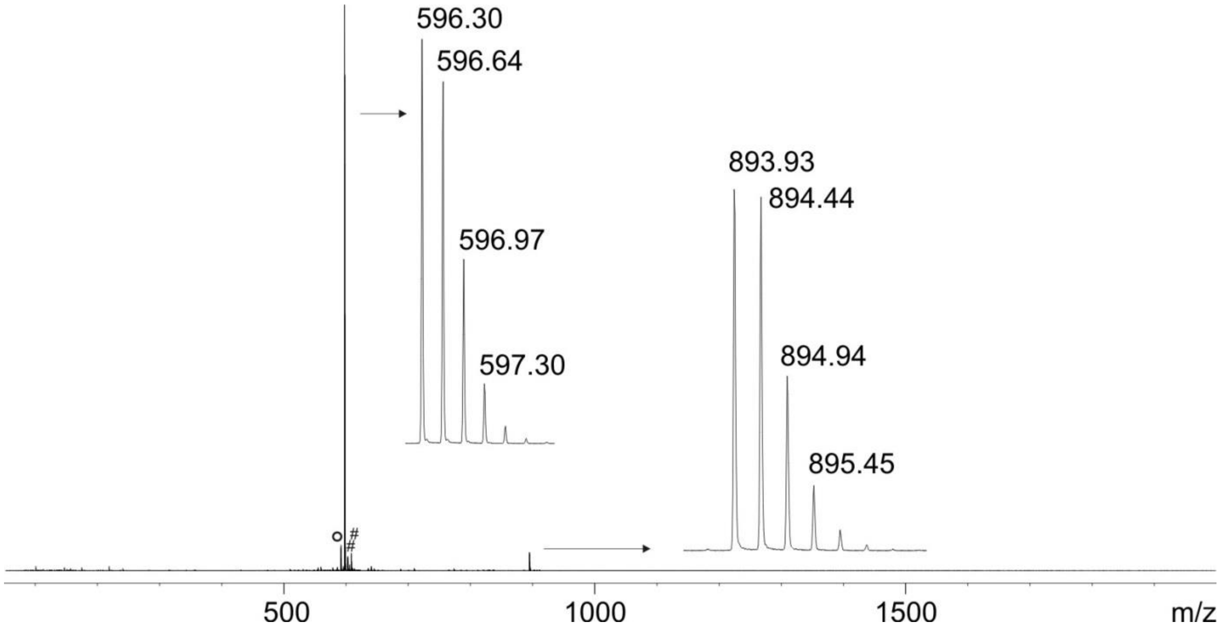

Figure S2. Nano-ESI mass spectrum of Troponin I peptide 2 (ENQEVGDWRKNIDAL). The m/z values of the isotopically resolved triply and doubly charged peptide ion signals are given. #: oxidation; °: loss of water. Solvent: 2% acetic acid, 10% methanol.

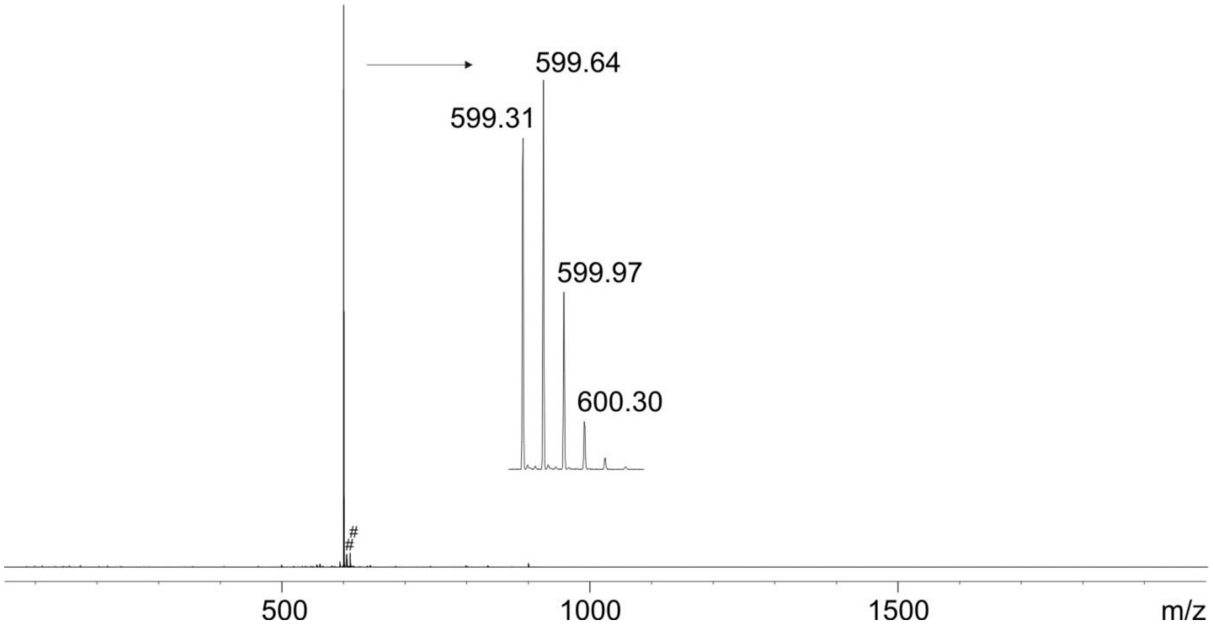

Figure S3. Nano-ESI mass spectrum of Troponin I peptide 3 (ENREVGDWHKNIDAL). The m/z values of the isotopically resolved triply charged peptide ion signal are given. #: oxidation. Solvent: 2% acetic acid, 10% methanol.

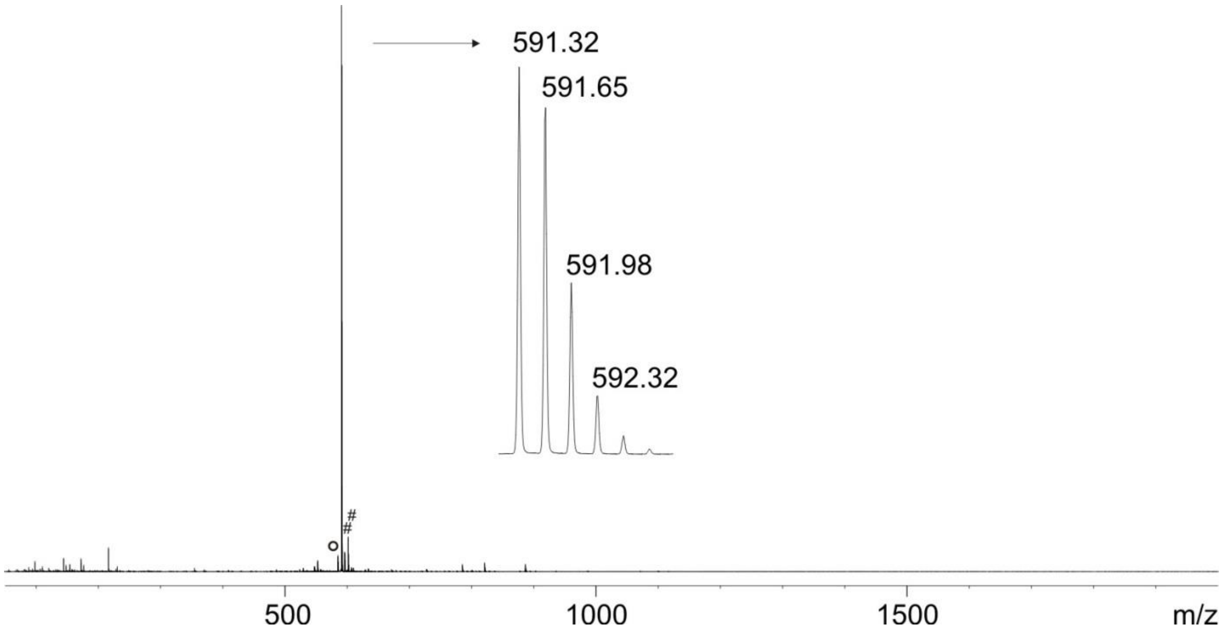

Figure S4. Nano-ESI mass spectrum of Troponin I peptide 4 (ENREVGDWLKNIDAL). The m/z values of the isotopically resolved triply charged peptide ion signal are given. #: oxidation; °: loss of water. Solvent: 2% acetic acid, 10% methanol.

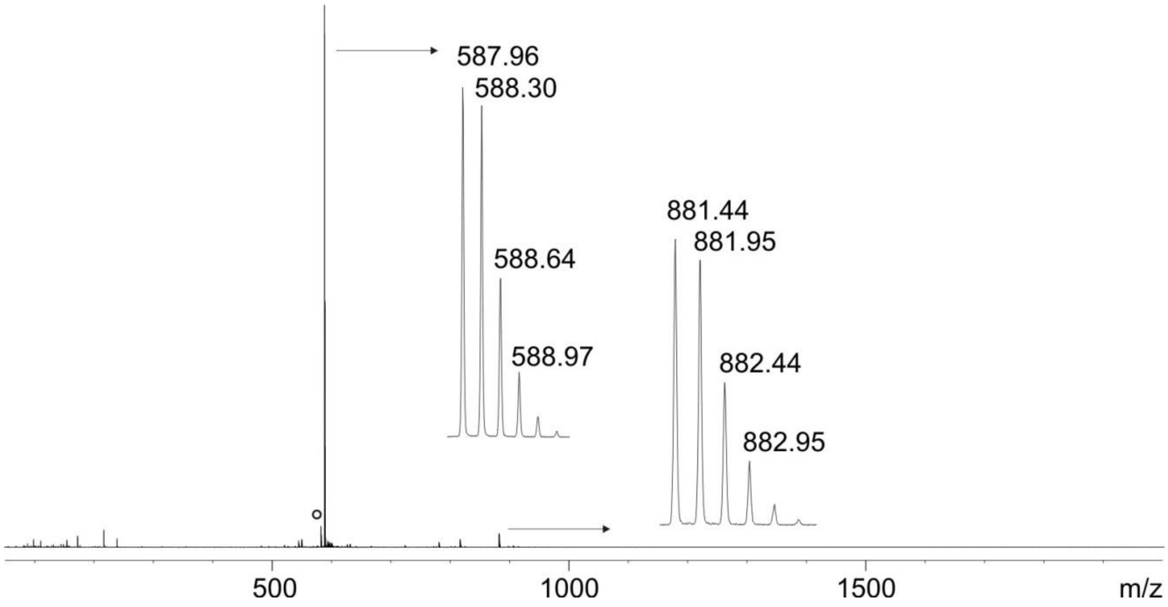

Figure S5. Nano-ESI mass spectrum of Troponin I peptide 5 (ENREVGDWCKNIDAL). The m/z values of the isotopically resolved triply and doubly charged peptide ion signals are given. °: loss of water. Solvent: 2% acetic acid, 10% methanol.

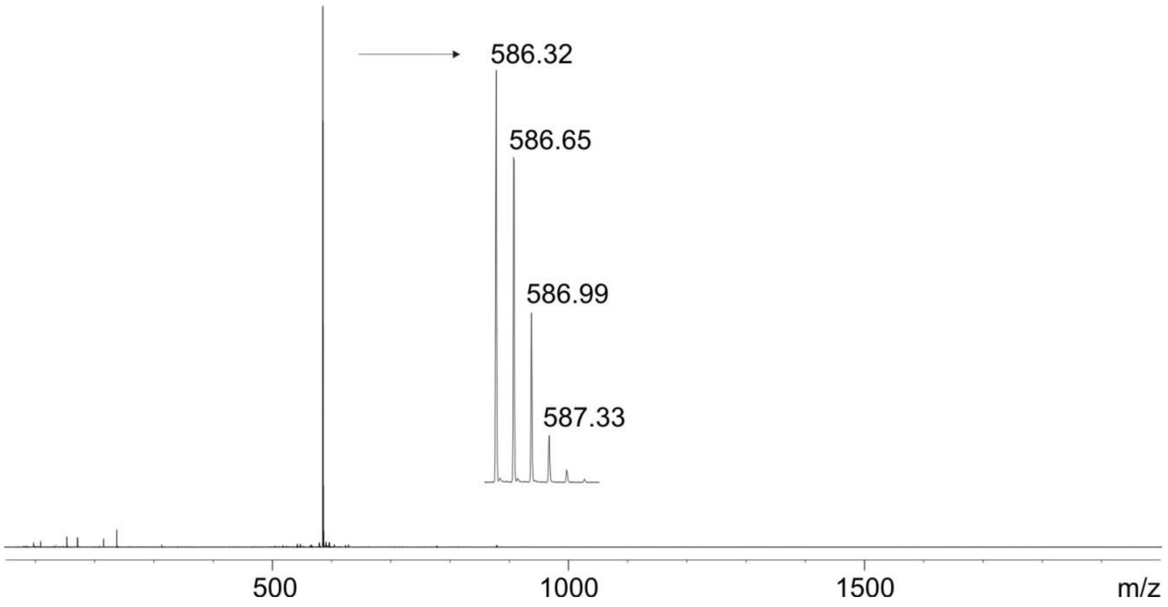

Figure S6. Nano-ESI mass spectrum of Troponin I peptide 6 (ENREVGGWRKNIDAL). The m/z values of the isotopically resolved triply charged peptide ion signal are given. Solvent: 2% acetic acid, 10% methanol.

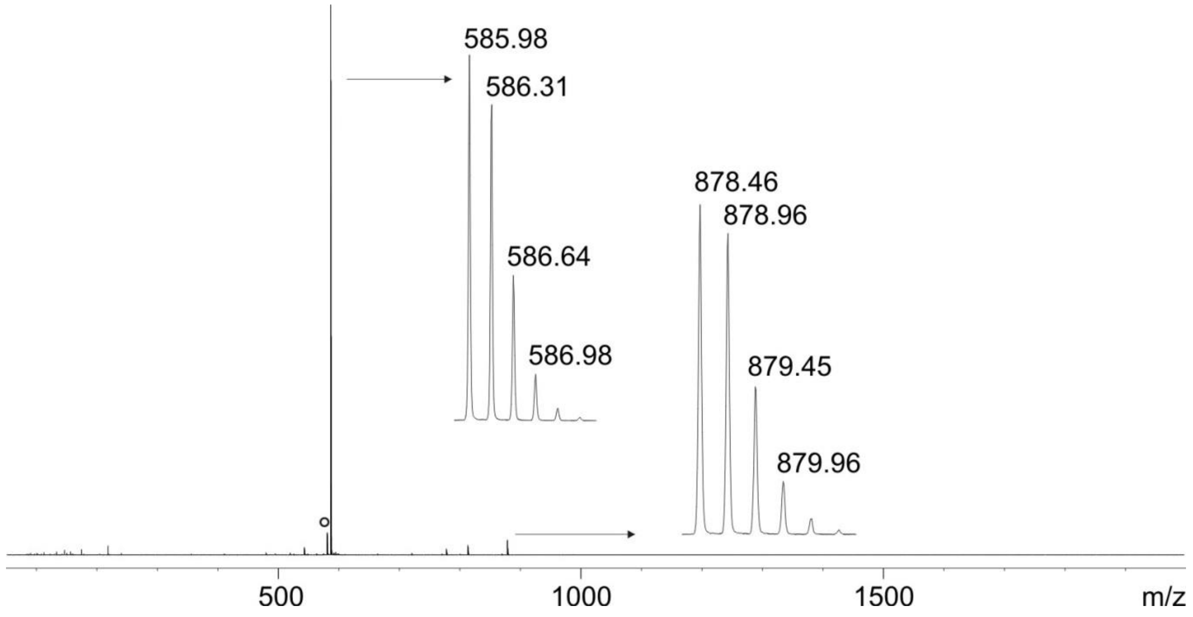

Figure S7. Nano-ESI mass spectrum of Troponin I peptide 7 (ENREVGDWPKNIDAL). The m/z values of the isotopically resolved triply and doubly charged peptide ion signals are given. °: loss of water. Solvent: 2% acetic acid, 10% methanol.

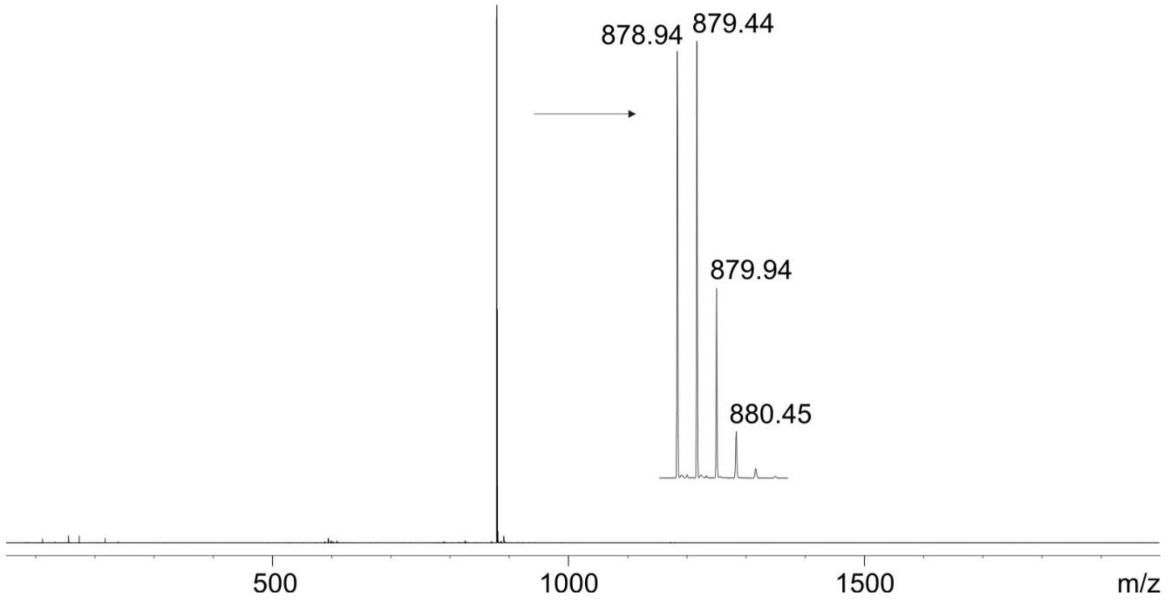

Figure S8. Nano-ESI mass spectrum of Troponin I peptide 8 (ENREVGDWPENIDAL). The m/z values of the isotopically resolved doubly charged peptide ion signal are given. Solvent: 2% acetic acid, 10% methanol.

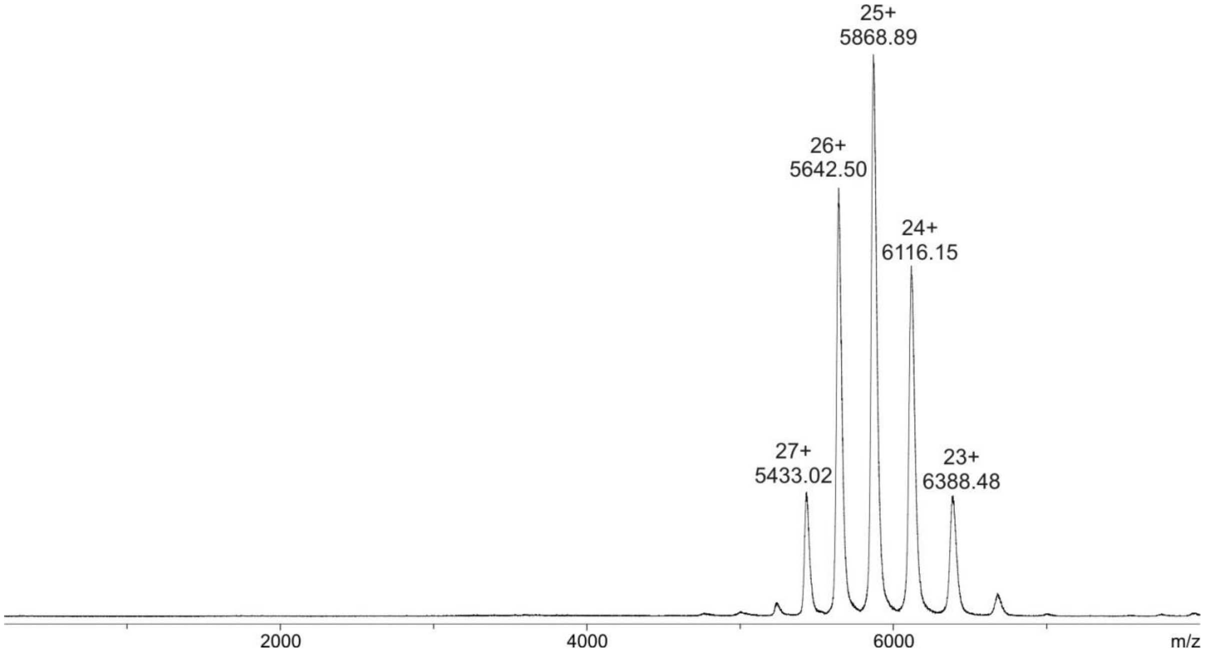

Figure S9. Nano-ESI mass spectra of the anti-Troponin I antibody. Charge states and m/z values are given. Solvent: 200 mM ammonium acetate, pH 6.7.

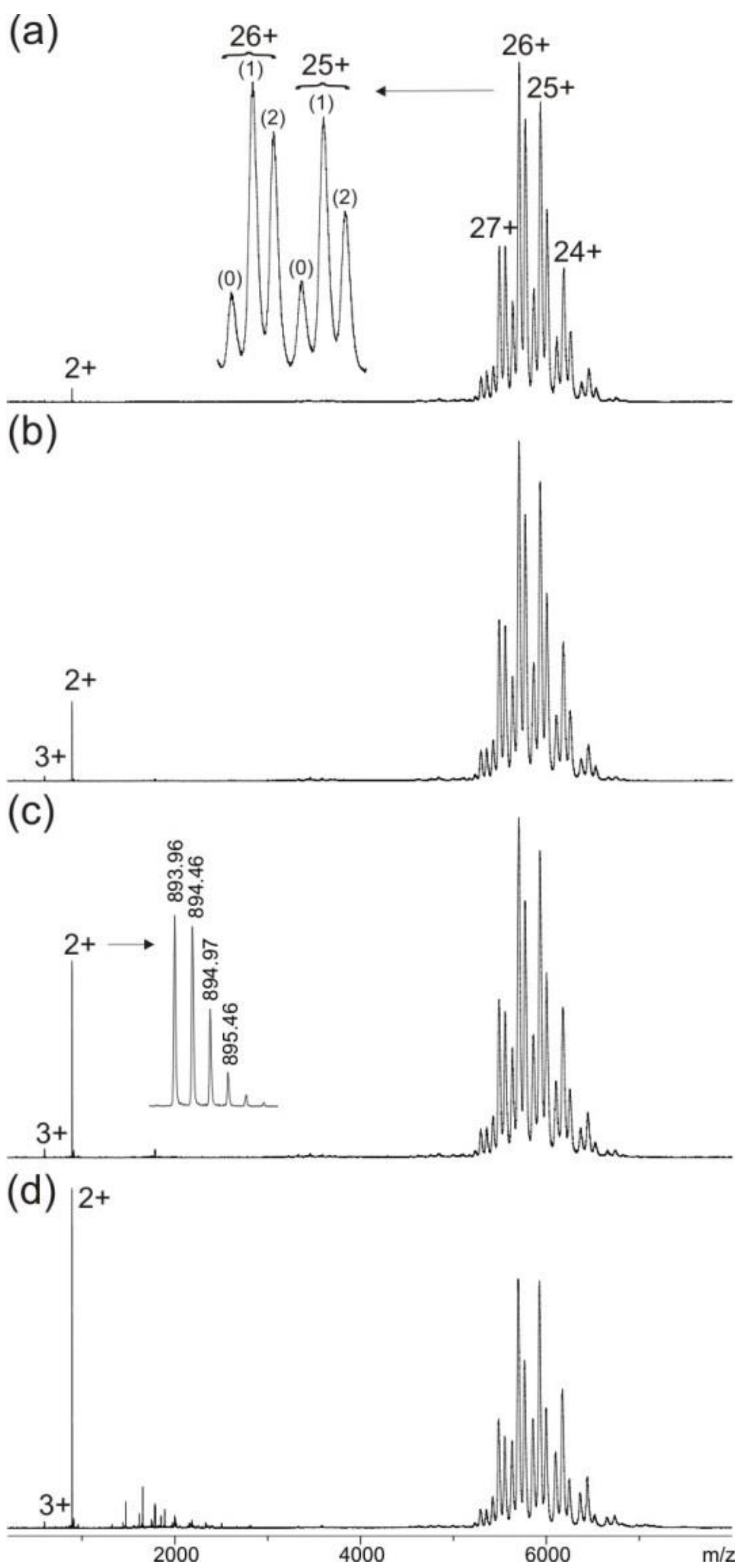

Figure S10. Nano-ESI mass spectra of Troponin I peptide 2 (ENQEVGDWRKNIDAL) – anti-Troponin I antibody immune complex with increasing collision cell voltage differences (ΔCV): (a) 4 V, (b) 16 V, (c) 30 V, (d) 80 V. Charge states are given for the immune complex (right ion series) and inlet in (a) shows a zoom of the 25+ and 26 + ion signals of the antibody (0) and the immune complex (antibody plus one peptide (1) and antibody plus two peptides (2)). Charge states and m/z values for peptide ion signals are given on the left and inlet in (c) shows a zoom of the isotopically resolved peptide ion signal. Antibody fragment ion signals are visible between m/z 1200 and 2300 in (d). Solvent: 200 mM ammonium acetate, pH 6.7.

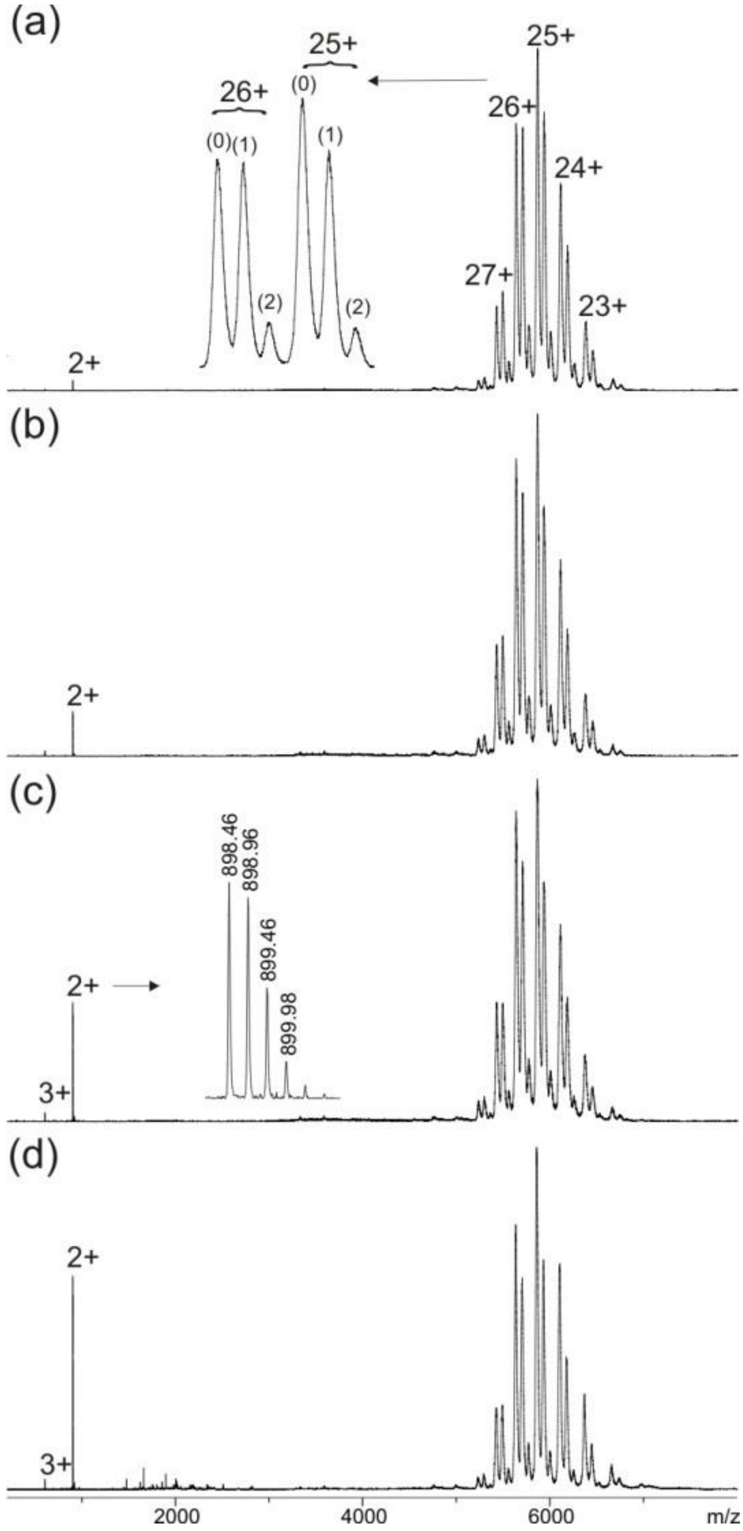

Figure S11. Nano-ESI mass spectra of Troponin I peptide 3 (ENREVGDWHKNIDAL) – anti-Troponin I antibody immune complex with increasing collision cell voltage differences (ΔCV): (a) 4 V, (b) 16 V, (c) 30 V, (d) 80 V. Charge states are given for the immune complex (right ion series) and inlet in (a) shows a zoom of the 25+ and 26 + ion signals of the antibody (0) and the immune complex (antibody plus one peptide (1) and antibody plus two peptides (2)). Charge states and m/z values for peptide ion signals are given on the left and inlet in (c) shows a zoom of the isotopically resolved peptide ion signal. Antibody fragment ion signals are visible between m/z 1200 and 2300 in (d). Solvent: 200 mM ammonium acetate, pH 6.7.

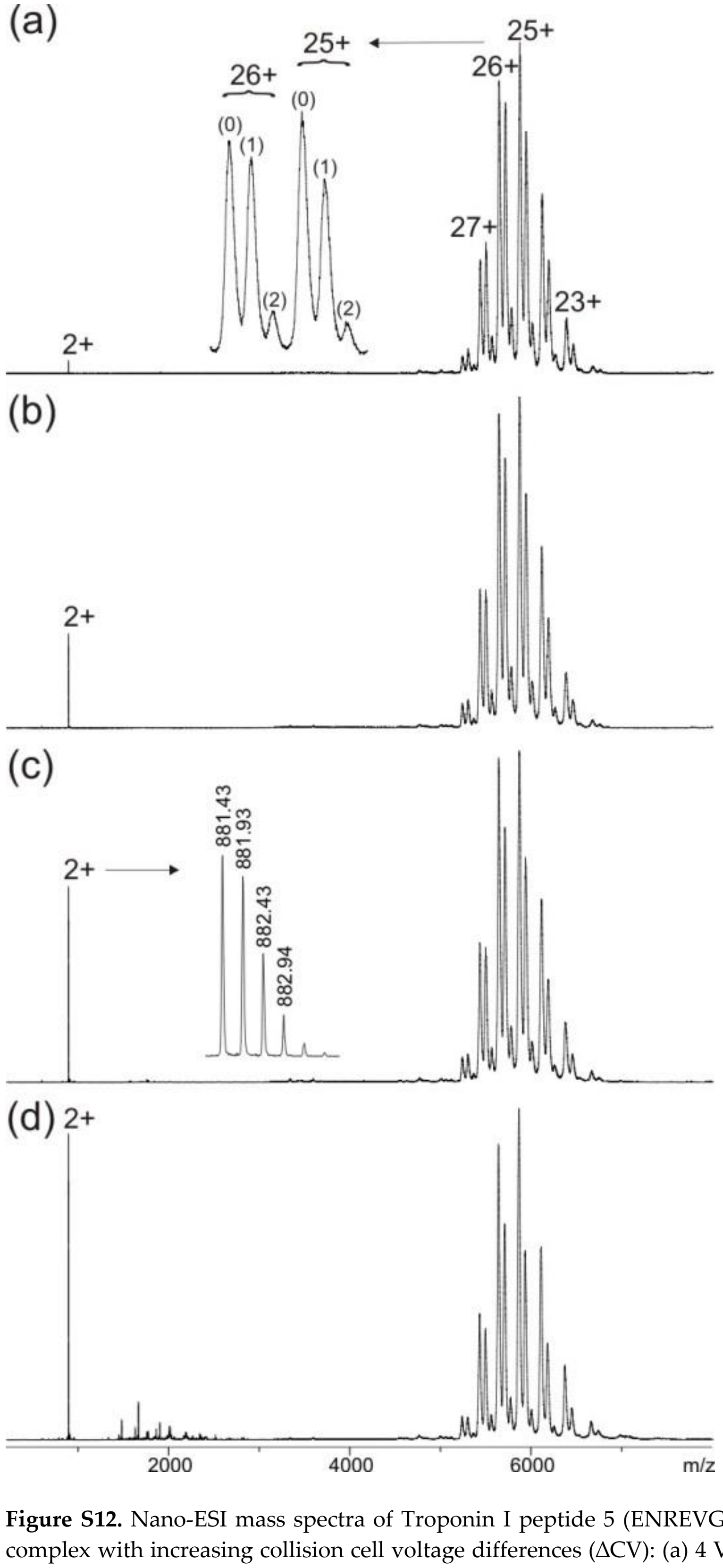

Figure S12. Nano-ESI mass spectra of Troponin I peptide 5 (ENREVGDWCKNIDAL) – anti-Troponin I antibody immune complex with increasing collision cell voltage differences (ΔCV): (a) 4 V, (b) 16 V, (c) 30 V, (d) 80 V. Charge states are given for the immune complex (right ion series) and inlet in (a) shows a zoom of the 25+ and 26 + ion signals of the antibody (0) and the immune complex (antibody plus one peptide (1) and antibody plus two peptides (2)). Charge states and m/z values for peptide ion signals are given on the left and inlet in (c) shows a zoom of the isotopically resolved peptide ion signal. Antibody fragment ion signals are visible between m/z 1200 and 2300 in (d). Solvent: 200 mM ammonium acetate, pH 6.7.

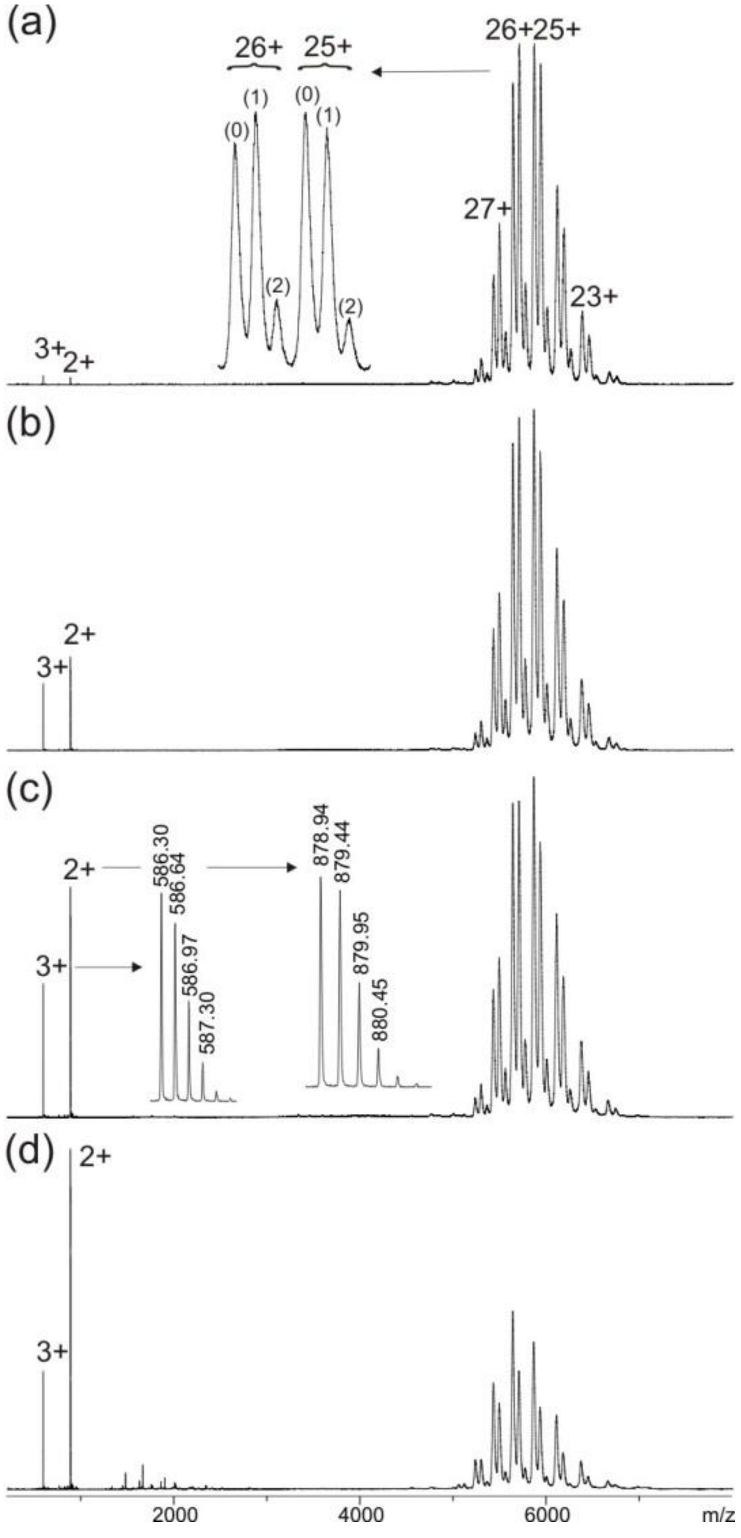

Figure S13. Nano-ESI mass spectra of Troponin I peptide 6 (ENREVGGWRKNIDAL) – anti-Troponin I antibody immune complex with increasing collision cell voltage differences (ΔCV): (a) 4 V, (b) 16 V, (c) 30 V, (d) 80 V. Charge states are given for the immune complex (right ion series) and inlet in (a) shows a zoom of the 25+ and 26 + ion signals of the antibody (0) and the immune complex (antibody plus one peptide (1) and antibody plus two peptides (2)). Charge states and m/z values for peptide ion signals are given on the left and inlet in (c) shows a zoom of the isotopically resolved peptide ion signals. Antibody fragment ion signals are visible between m/z 1200 and 2300 in (d). Solvent: 200 mM ammonium acetate, pH 6.7.

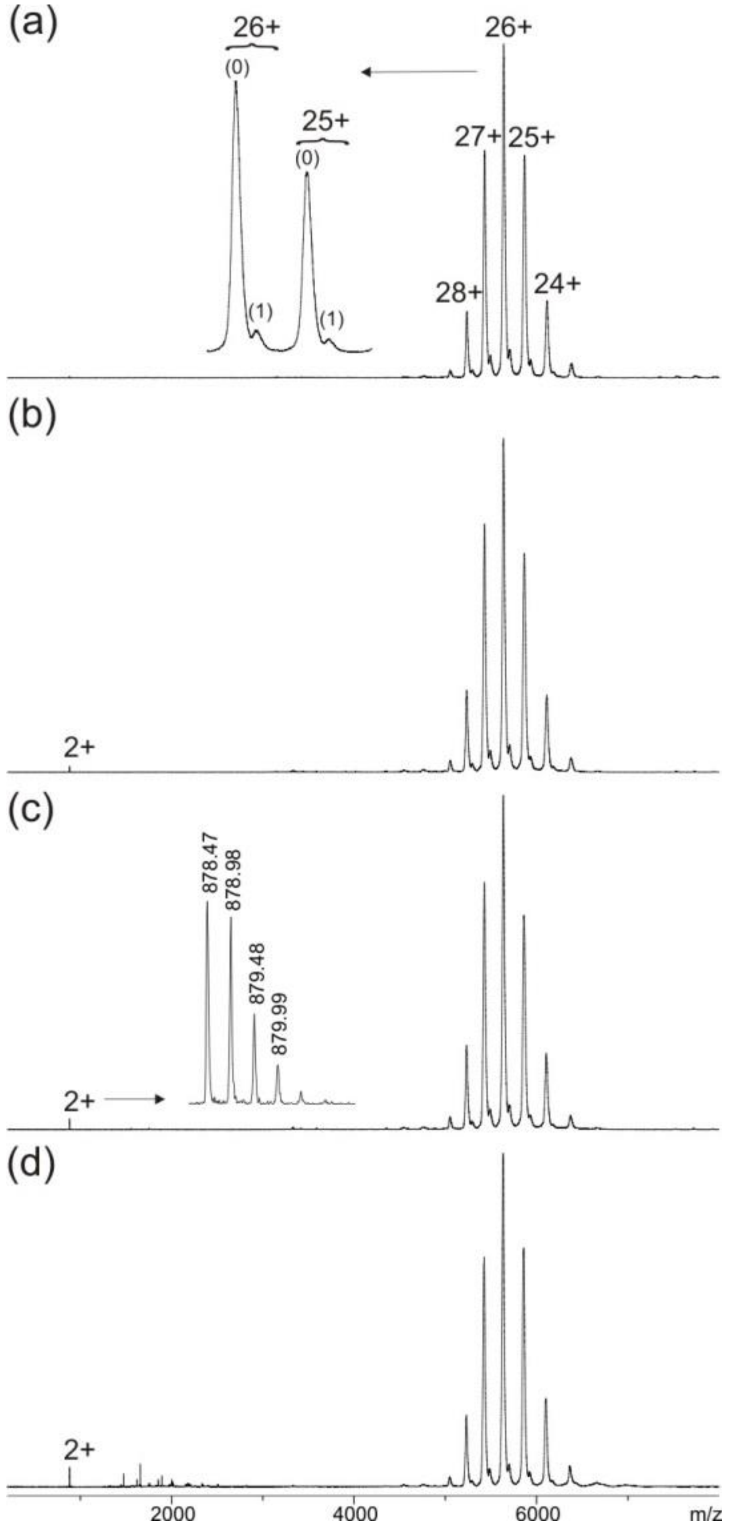

Figure S14. Nano-ESI mass spectra of Troponin I peptide 7 (ENREVGDWPKNIDAL) – anti-Troponin I antibody immune complex with increasing collision cell voltage differences (ΔCV): (a) 4 V, (b) 16 V, (c) 30 V, (d) 80 V. Charge states are given for the immune complex (right ion series) and inlet in (a) shows a zoom of the 25+ and 26 + ion signals of the antibody (0) and the immune complex (antibody plus one peptide (1)). Charge states and m/z values for peptide ion signals are given on the left and inlet in (c) shows a zoom of the isotopically resolved peptide ion signal. Antibody fragment ion signals are visible between m/z 1200 and 2300 in (d). Solvent: 200 mM ammonium acetate, pH 6.7.

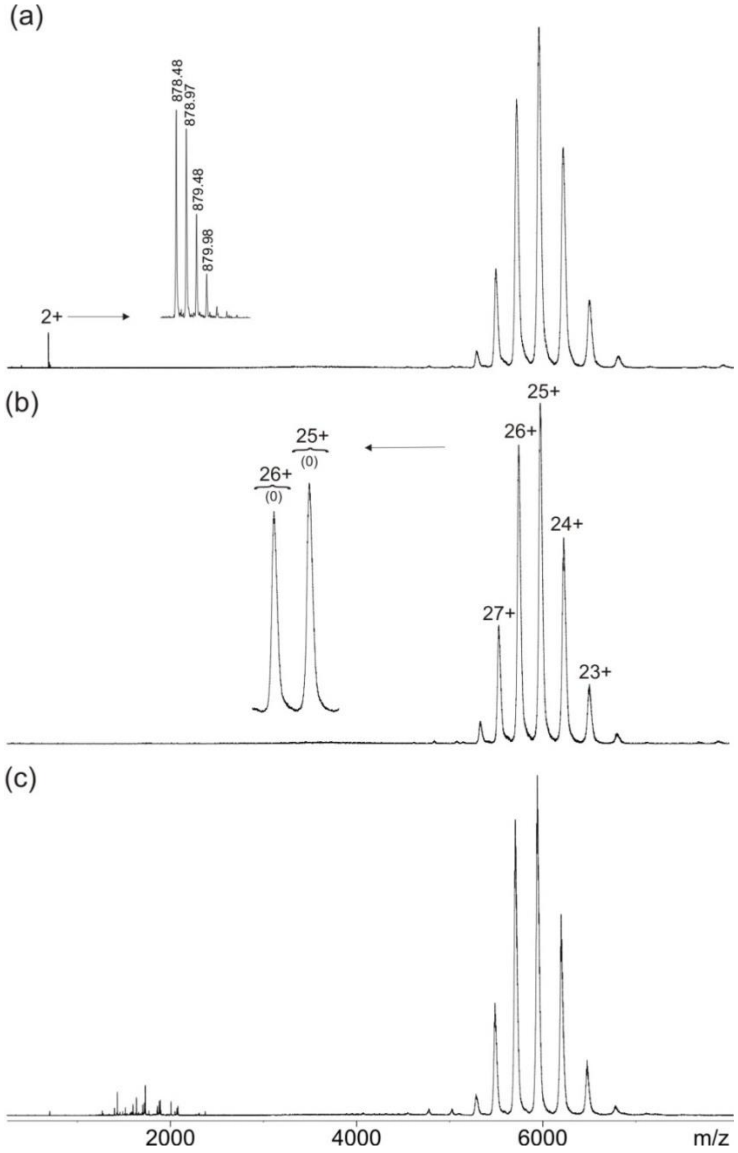

Figure S15. Nano-ESI mass spectra of Troponin I peptide 7 (ENREVGDWPKNIDAL) upon incubation with anti-Histag antibody with (a) unblocked ion transmission and collision cell voltage difference of 2 V (inlet shows zoom of the peptide ion signal) and (b) with blocked transmission of low mass ions (< 4800) and collision cell voltage differences of 2V and (c) with blocked transmission of low mass ion (< 4800) and collision cell voltage differences of 80V. Charge states are given for the antibody ion signal (right ion series) and inlet in (b) shows a zoom of the 25+ and 26 + ion signals of the antibody (0). Antibody fragment ion signals are visible between m/z 1200 and 2300 in (c). Solvent: 200 mM ammonium acetate, pH 6.7.

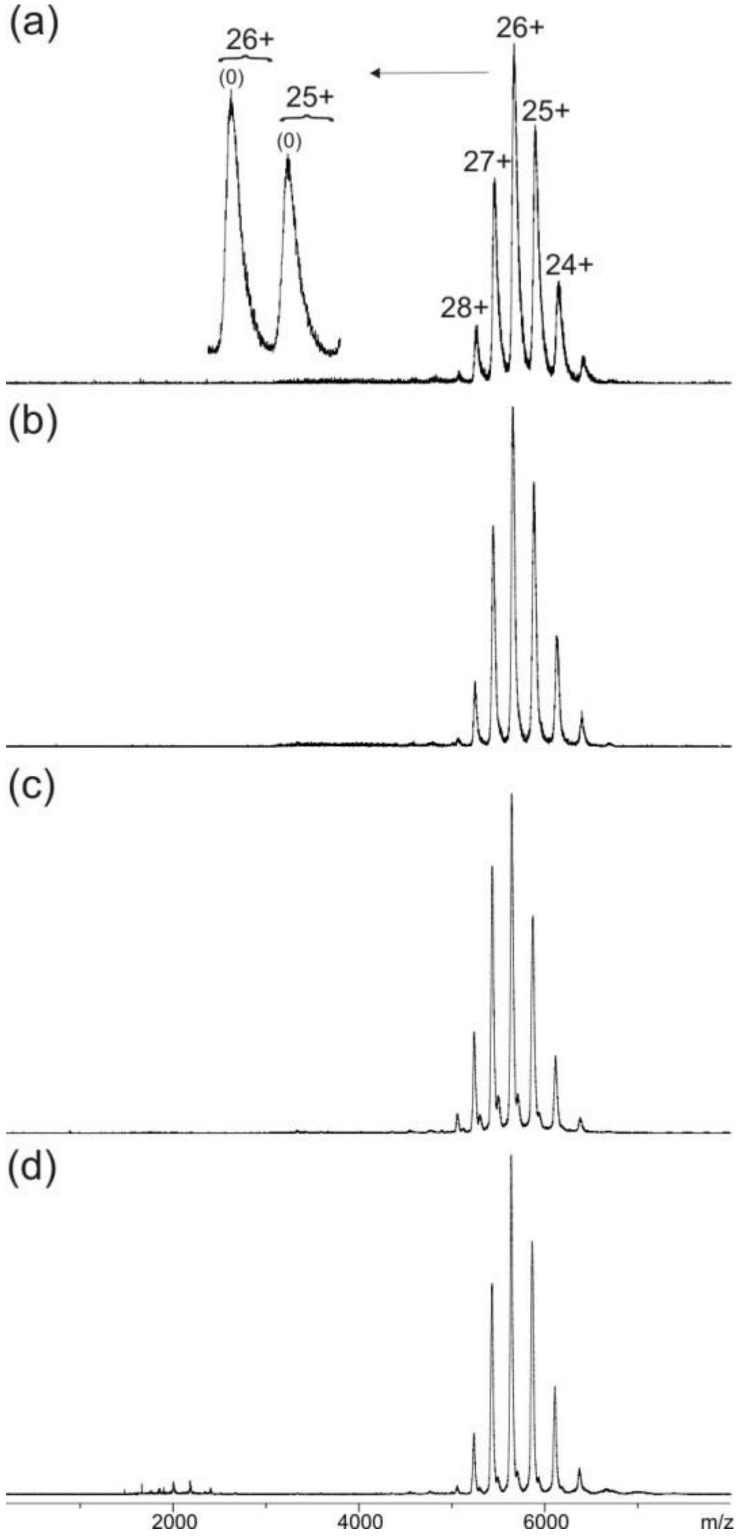

Figure S16. Nano-ESI mass spectra of Troponin I peptide 8 (ENREVGDWPENIDAL) after incubation with anti-Troponin I antibody with increasing collision cell voltage differences (ΔCV): (a) 4 V, (b) 16 V, (c) 30 V, (d) 80 V. Charge states are given for the antibody ion signal (right ion series) and inlet in (a) shows a zoom of the 25+ and 26 + ion signals of the antibody (0). Antibody fragment ion signals are visible between m/z 1200 and 2300 in (d). Solvent: 200 mM ammonium acetate, pH 6.7.

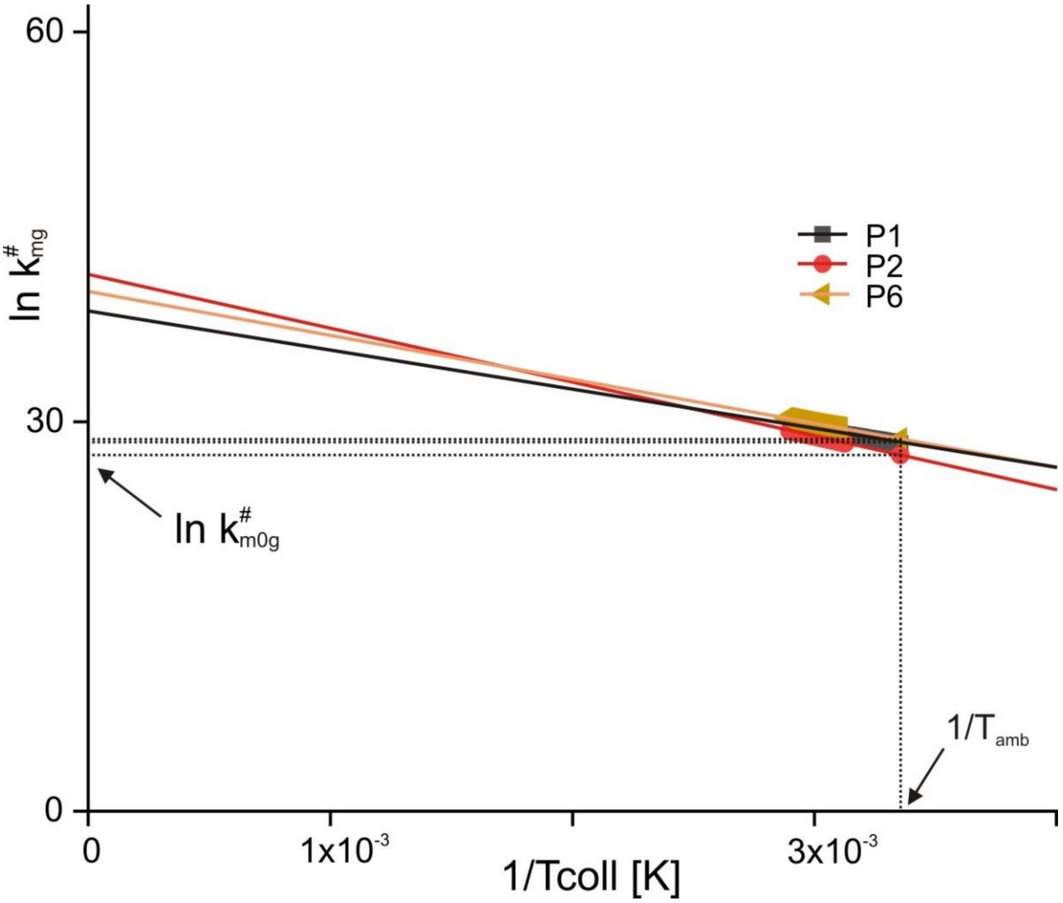

Figure S17. Arrhenius plot for the immune complex dissociations of anti-hcTroponin I antibody and human cardiac Troponin I peptides 1 (black square), 2 (red circle), and 6 (orange triangle) in the gas phase. Each data point (thickened parts of the lines) has been obtained experimentally, corresponding lines have been extrapolated linearly. The values for lnk#m0g are taken at $1/T_{amb}$.

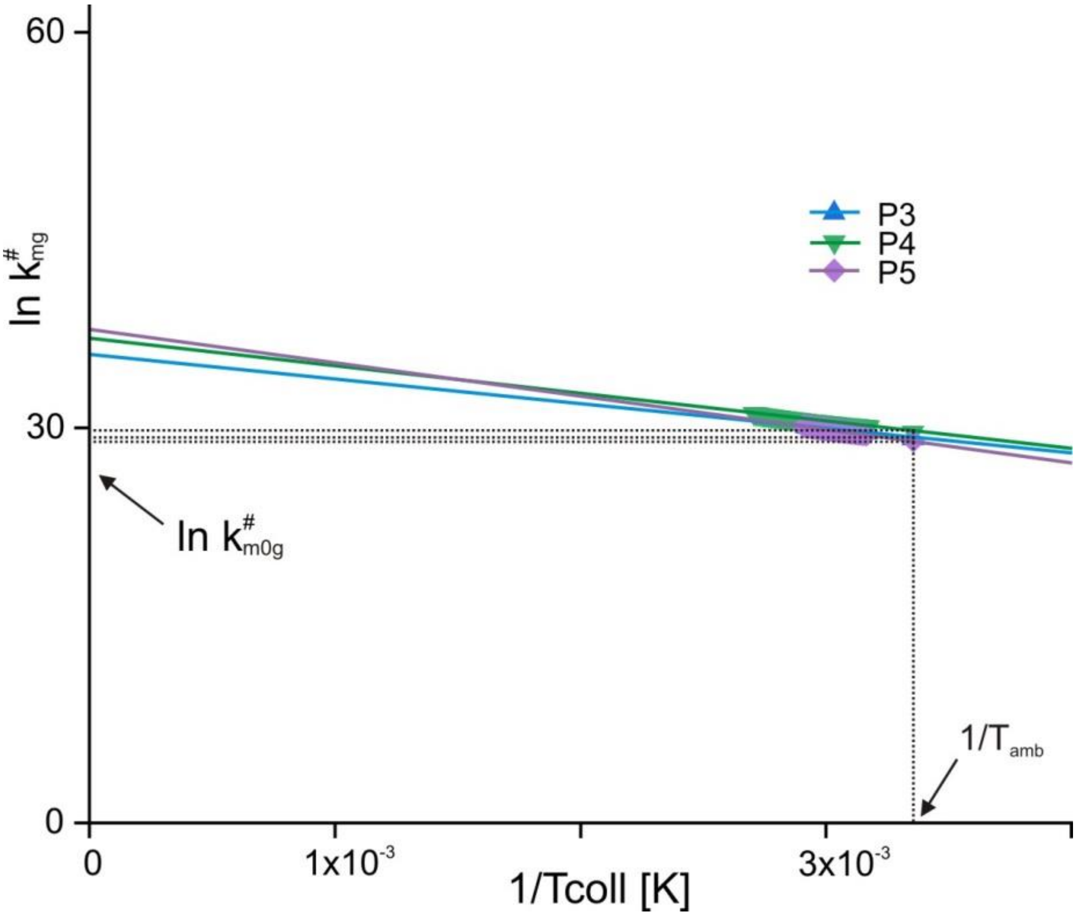

Figure S18. Arrhenius plot for the immune complex dissociations of anti-hcTroponin I antibody and human cardiac Troponin I peptides 3 (blue triangle), 4 (green triangle), and 5 (purple square) in the gas phase. Each data point (thickened parts of the lines) has been obtained experimentally, corresponding lines have been extrapolated linearly. The values for lnk#m0g are taken at $1/T_{amb}$.

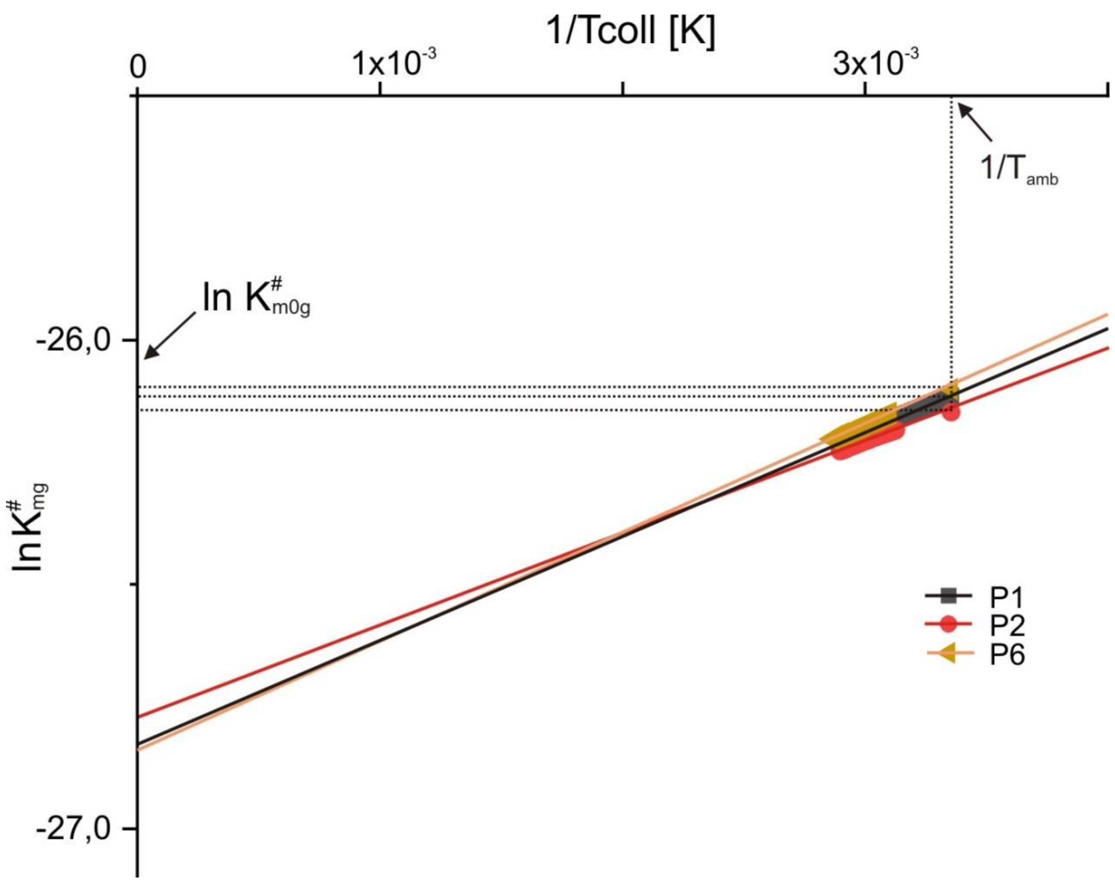

Figure S19. Gibbs-Helmholtz plot for the immune complex dissociations of anti-hcTroponin I antibody and human cardiac Troponin I peptides 1 (black square), 2 (red circle), and 6 (orange triangle) in the gas phase. Each data point (thickened parts of the lines) has been obtained experimentally, corresponding lines have been extrapolated linearly. The values for lnK#m0g are taken at $1/T_{amb}$. Calculated kinetic and thermodynamic values are listed in Table 3.

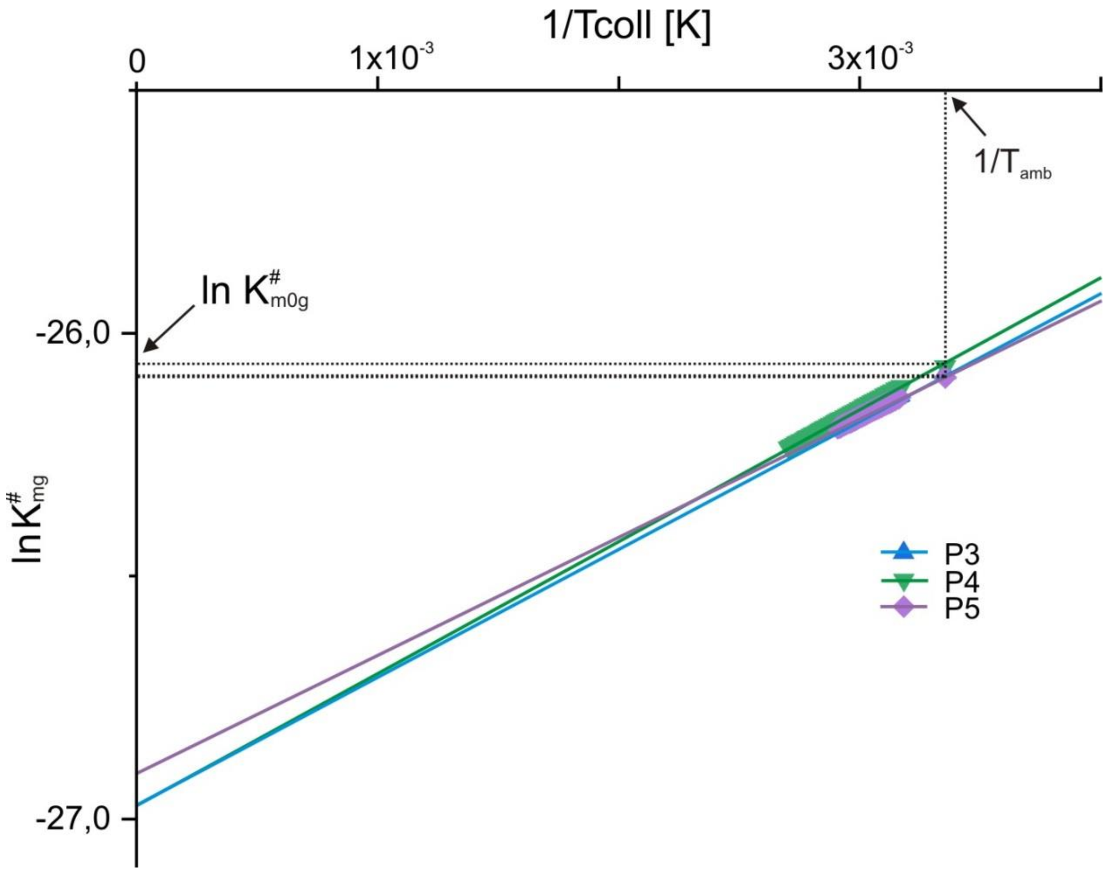

Figure S20. Gibbs-Helmholtz plot for the immune complex dissociations of anti-hcTroponin I antibody and human cardiac Troponin I peptides 3 (blue triangle), 4 (green triangle), and 5 (purple square) in the gas phase. Each data point (thickened parts of the lines) has been obtained experimentally, corresponding lines have been extrapolated linearly. The values for lnK#m0g are taken at $1/T_{amb}$. Calculated kinetic and thermodynamic values are listed in Table 3.

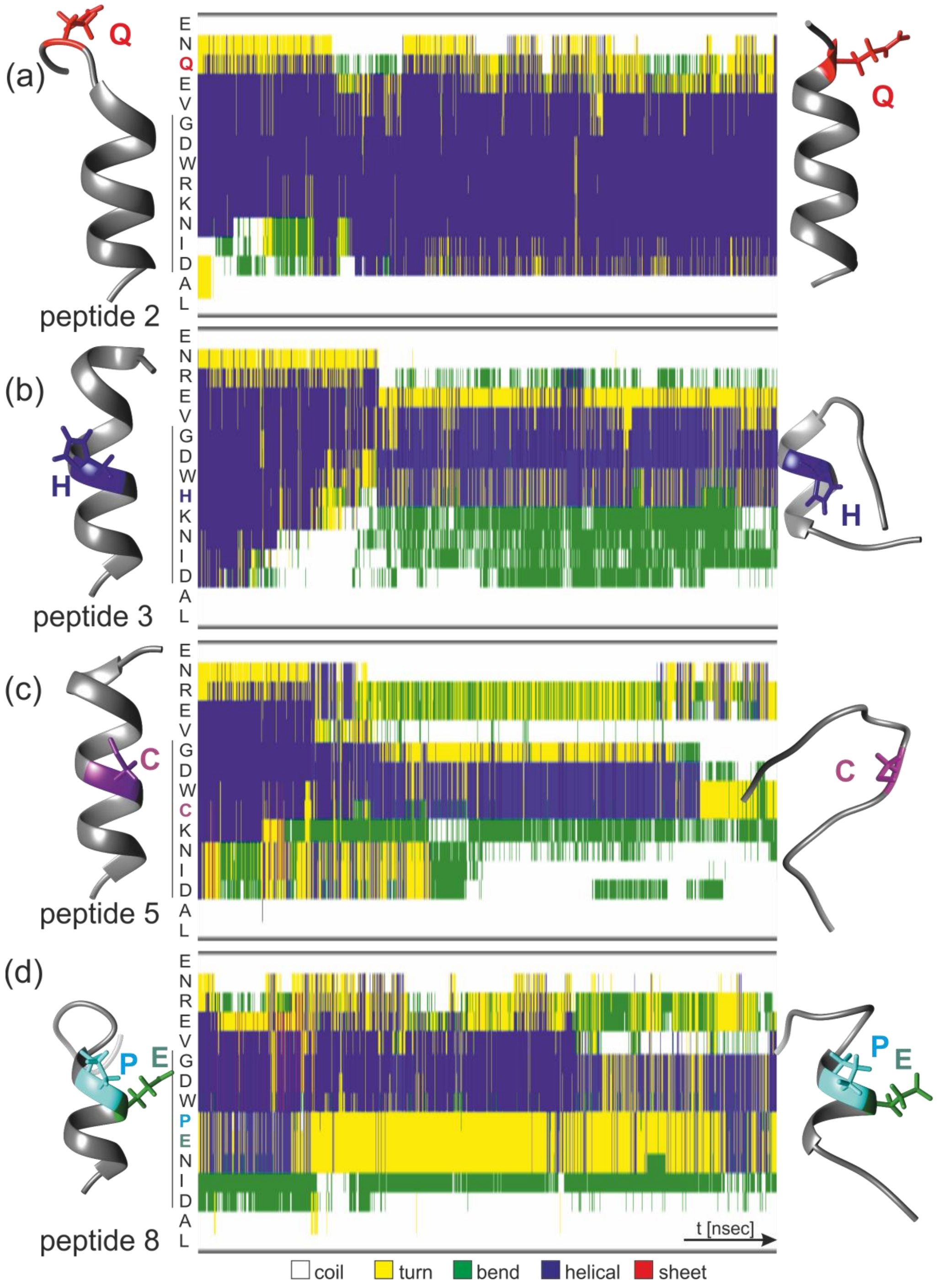

Figure S21. Peptide structure models and secondary structure elements prior to and after molecular dynamics simulations. Predicted epitope peptide model structures (left) were compared to structure models after 50 ns simulation (right). (a) peptide 2, (b) peptide 3, (c) peptide 5, and (d) peptide 8. Amino acid residues of peptides are listed from top to bottom (center). The vertical line at the left indicates the epitope region. The secondary structure element into which each residue is involved in at a given simulation time point is depicted from left to right as color coded bar (10,000 bars per line). Color code: white: coil; yellow: turn; green: bend; blue: helical; red: sheet.

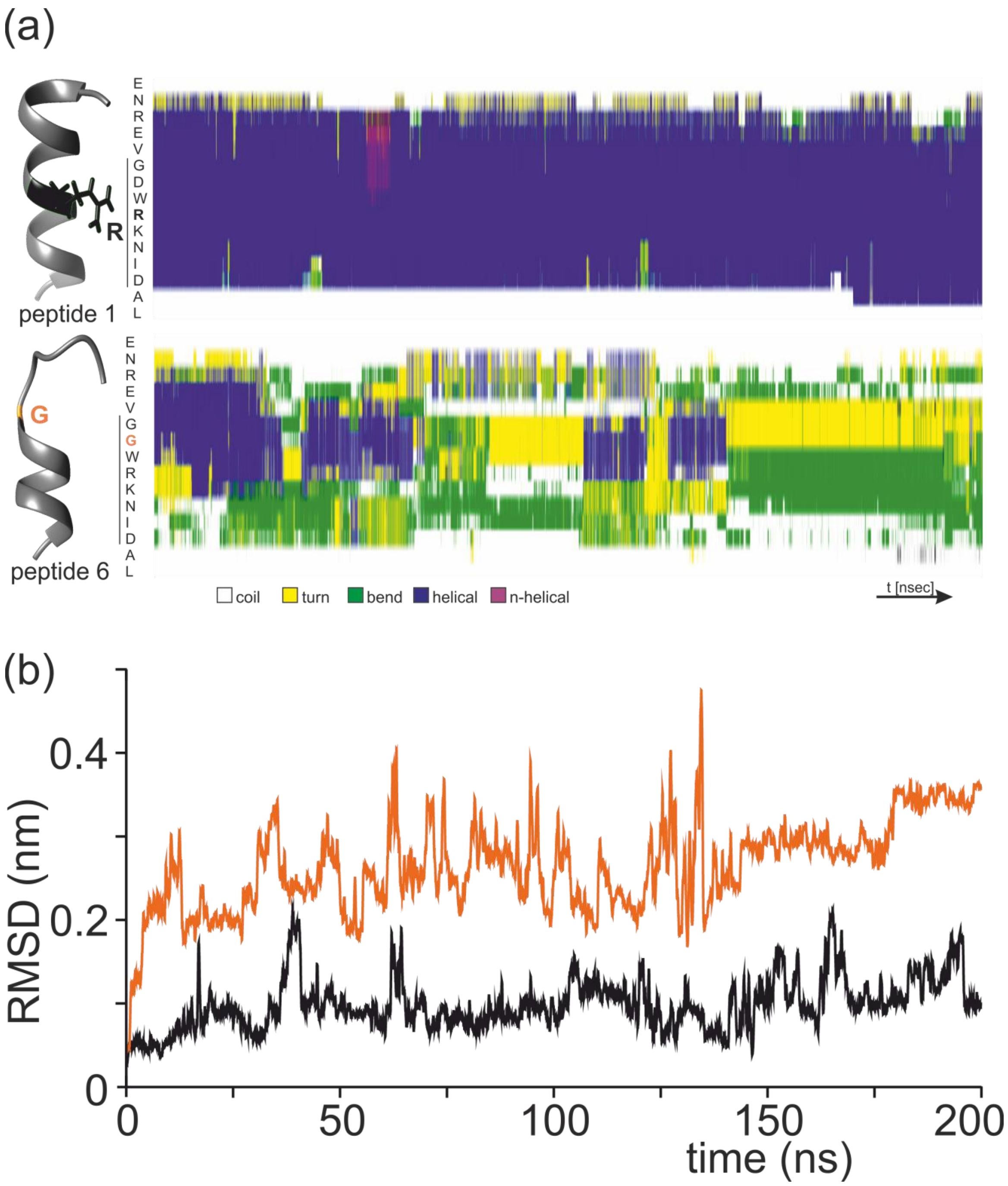

Figure S22. Peptide structure models and secondary structure elements prior to and after molecular dynamics simulations. (a) Predicted epitope peptide model structures (left) were compared to structure models after 200 ns simulation. Upper panel: peptide 1, lower panel: peptide 6. Amino acid residues of peptides are listed from top to bottom (center). The vertical line at the left indicates the epitope region. The secondary structure element into which each residue is involved in at a given simulation time point is depicted from left to right as color coded bar (40,000 bars per line). Color code: white: coil; yellow: turn; green: bend; blue: helical; purple: n-helix. (b) Positional flexibilities of backbone atoms of epitope peptides 1 (black trace) and 6 (orange trace). Root mean squared deviation (RMSD) as a function of simulation time.

Table S1. Numbering, amino acid sequences, SNP information, molecular masses, isoelectric points, numbers of atoms of human cardiac Troponin I peptides and number of ITEM-FOUR measurement repetitions.

peptide no.	amino acid sequence [a)]	base position [b)]	clin. var. ID [c)]	molecular mass	pI [d)]	number of atoms [e)]	ITEM-FOUR repetitions
1	ENREVGDWRKNIDAL	n.a.	n.a.	1813.91	4.6	251	7
2	EN**Q**EVGDWRKNIDAL	55,151,910	RCV000167988.8	1785.86	4.1	245	5
3	ENREVGDW**H**KNIDAL	55,151,892	RCV000156328.2	1794.86	4.6	245	2
4	ENREVGDW**L**KNIDAL	55,151,892	RCV000156328.2	1770.89	4.1	247	5
5	ENREVGDW**C**KNIDAL	55,151,893	RCV000152072.3	1760.82	4.1	237	5
6	ENREVG**G**WRKNIDAL	55,151,898	RCV000013235.24	1755.90	7.1	245	4
7	ENREVGDW**P**KNIDAL	55,151,892	RCV000156328.2	1754.86	4.1	242	2
8	ENREVGDW**PE**NIDAL	55,151,892<br>55,151,890	RCV000156328.2<br>RCV001296186.2	1755.81	3.5	237	2

a) aa184-aa198 from Tn I (UniProt: P19429); the epitope region of the monoclonal anti-hcTroponin I antibody (clone MF4, ab38210 from abcam) is underlined; amino acid exchanges in peptides 2-8 are printed in bold and are colored

b) position on chromosome 19 (exon 8)

c) https://www.ncbi.nlm.nih.gov/clinvar/; n.a.: not applicable

d) calculated according to Skoog&Wichmann (reference no.)

e) calculated from amino acid composition

Table S2. Ion charge states, m/z values, and molecular masses for anti-hcTn I antibody and rituximab.

	anti-hcTn I measurement 1 [a)]		anti-hcTn I measurement 2 [a)]		anti-hcTn I measurement 3 [a)]		rituximab measurement 1 [a)]		rituximab measurement 2 [a)]	
z	m/z	MM	m/z	MM	m/z	MM	m/z	MM	m/z	MM
29	n.d.	n.d.	n.d.	n.d.	n.d.	n.d.	5086.8	147487.9	5085.7	147455.4
28	5246.7	146879.6	n.d.	n.d.	n.d.	n.d.	5266.8	147443.5	5266.2	147425.9
27	5437.1	146775.5	5435.0	146716.7	5433.0	146664.5	5463.1	147477.2	5460.6	147410.0
26	5646.0	146770.0	5643.7	146710.5	5642.5	146679.0	5674.6	147514.6	5670.6	147410.4
25	5875.7	146867.5	5870.5	146736.5	5868.9	146697.3	5902.2	147528.8	5898.0	147425.5
24	6121.0	146880.5	6116.3	146767.4	6116.2	146763.6	n.d.	n.d.	n.d.	n.d.
mean		146834.6		146732.8		146701.1		147490.4		147425.4
s.d.		56.7		25.6		43.8		33.3		18.5

a) n.d.: not determined; MM: molecular mass; s.d.: standard deviation

Table S3. Ion species, charge states, m/z values and intensities for anti-Troponin I antibody complexed with Troponin I peptide 1 at measured collision cell voltage differences. [a,b)]

peptide 1, measurement 1

ion / charge state	m/z	ΔCV										
		**4**	**8**	**12**	**16**	**20**	**30**	**40**	**50**	**60**	**70**	**80**
**peptide dimer / 1+**	3628.90	5	10	10	10	10	10	10	10	10	10	10
**peptide 1 / 1+**	1814.95	5	11	10	10	10	14	18	23	42	51	81
**peptide 1 / 2+**	907.99	25	121	273	578	984	1988	3216	4454	6528	7557	7827
**peptide 1 / 3+**	605.71	8	53	83	196	329	546	802	1130	1545	1633	1617
**peptide 1 / 4+**	454.49	5	10	10	10	10	10	10	10	10	10	10
**antibody+0 pep / 28+**	5234	50	50	50	50	50	50	50	50	50	50	50
**antibody+0 pep / 27+**	5424	52	116	114	129	139	130	135	133	133	132	145
**antibody+0 pep / 26+**	5634	367	812	848	961	1061	995	1054	1071	1078	1132	1185
**antibody+0 pep / 25+**	5861	1134	2488	2514	2969	3160	3056	3251	3412	3572	3568	3720
**antibody+0 pep / 24+**	6105	1546	3549	3705	4175	4409	4365	4887	4951	5273	5186	5144
**antibody+0 pep / 23+**	6374	1113	2496	2728	2996	3079	3140	3627	3655	3985	4063	4070
**antibody+0 pep / 22+**	6671	434	1001	1105	1164	1168	1256	1433	1554	1747	1791	1779
**antibody+0 pep / 21+**	6979	50	50	50	50	50	50	50	50	50	50	50
**antibody+1 pep/ 28+**	5300	50	50	50	50	50	50	50	50	50	50	50
**antibody+1 pep/ 27+**	5493	107	218	230	264	274	246	249	247	254	237	255
**antibody+1 pep/ 26+**	5702	628	1385	1373	1620	1685	1577	1613	1645	1629	1621	1674
**antibody+1 pep/ 25+**	5935	1500	3230	3258	3787	3981	3803	4011	4056	4143	4111	4111
**antibody+1 pep/ 24+**	6182	1726	3841	4066	4527	4615	4549	4917	4912	5233	5031	5012
**antibody+1 pep/ 23+**	6454	1002	2193	2443	2581	2612	2694	2922	3106	3146	3159	3169
**antibody+1 pep/ 22+**	6754	345	759	871	863	896	915	1021	1092	1157	1212	1203
**antibody+1 pep/ 21+**	7066	50	50	50	50	50	50	50	50	50	50	50
**antibody+2 peps/ 28+**	5364	50	50	50	50	50	50	50	50	50	50	50
**antibody+2 peps/ 27+**	5555	64	134	138	149	151	139	131	135	137	125	132
**antibody+2 peps/ 26+**	5771	249	537	529	594	619	592	573	609	577	518	556
**antibody+2 peps/ 25+**	6007	485	1044	1079	1240	1236	1191	1237	1208	1186	1118	1133
**antibody+2 peps/ 24+**	6261	493	1082	1145	1237	1273	1209	1305	1277	1258	1241	1189
**antibody+2 peps/ 23+**	6532	229	492	580	587	592	584	627	620	618	622	585
**antibody+2 peps/ 22+**	6834	61	151	174	176	185	196	199	208	213	210	216
**antibody+2 peps/ 21+**	7151	50	50	50	50	50	50	50	50	50	50	50

**antibody fragment**	1000	n.d.	n.d.	n.d.	n.d.	n.d.	n.d.	n.d.	20	20	20	20
**antibody fragment**	1200	n.d.	n.d.	n.d.	n.d.	n.d.	n.d.	n.d.	20	20	20	20
**antibody fragment**	1324	n.d.	n.d.	n.d.	n.d.	n.d.	n.d.	20	n.d.	n.d.	n.d.	n.d.
**antibody fragment**	1471	n.d.	n.d.	n.d.	n.d.	n.d.	n.d.	20	n.d.	48	151	407
**antibody fragment**	1554	n.d.	n.d.	n.d.	n.d.	n.d.	n.d.	37	46	63	n.d.	n.d.
**antibody fragment**	1655	n.d.	n.d.	n.d.	n.d.	n.d.	n.d.	n.d.	n.d.	92	363	852
**antibody fragment**	1748	n.d.	n.d.	n.d.	n.d.	n.d.	n.d.	88	116	170	n.d.	n.d.
**antibody fragment**	1851	n.d.	n.d.	n.d.	n.d.	n.d.	n.d.	n.d.	n.d.	n.d.	n.d.	n.d.
**antibody fragment**	1891	n.d.	n.d.	n.d.	n.d.	n.d.	n.d.	n.d.	n.d.	n.d.	n.d.	n.d.
**antibody fragment**	1998	n.d.	n.d.	n.d.	n.d.	n.d.	n.d.	72	139	253	266	474
**antibody fragment**	2006	n.d.	n.d.	n.d.	n.d.	n.d.	n.d.	n.d.	n.d.	n.d.	n.d.	n.d.
**antibody fragment**	2160	n.d.	n.d.	n.d.	n.d.	n.d.	n.d.	20	n.d.	n.d.	n.d.	n.d.
**antibody fragment**	2174	n.d.	n.d.	n.d.	n.d.	n.d.	n.d.	20	n.d.	n.d.	n.d.	n.d.
**antibody fragment**	2189	n.d.	n.d.	n.d.	n.d.	n.d.	n.d.	n.d.	n.d.	n.d.	n.d.	n.d.
**antibody fragment**	2207	n.d.	n.d.	n.d.	n.d.	n.d.	n.d.	n.d.	n.d.	n.d.	n.d.	n.d.
**antibody fragment**	2331	n.d.	n.d.	n.d.	n.d.	n.d.	n.d.	n.d.	46	93	106	185
**antibody fragment**	2392	n.d.	n.d.	n.d.	n.d.	n.d.	n.d.	n.d.	n.d.	n.d.	n.d.	n.d.
**antibody fragment**	2508	n.d.	n.d.	n.d.	n.d.	n.d.	n.d.	n.d.	20	20	20	20
**antibody fragment**	2591	n.d.	n.d.	n.d.	n.d.	n.d.	n.d.	n.d.	20	20	20	20

a) Imputed values necessary for optimized Gauss-fit are shown in red and are equal to the background intensity at the given m/z.
b) n.d.: value not determined / ion signal not present.

Table S3. continued

peptide 1, measurement 2

**ion / charge state**	**m/z**	**ΔCV**												
		**2**	**4**	**6**	**8**	**12**	**16**	**20**	**30**	**40**	**50**	**60**	**70**	**80**
**peptide dimer / 1+**	3628.90	10	10	10	10	10	10	10	10	10	10	10	10	10
**peptide 1 / 1+**	1814.95	10	10	10	20	10	10	9	18	37	44	70	98	104
**peptide 1 / 2+**	907.99	105	164	281	416	823	1269	1708	2840	5622	7735	8646	8983	8831
**peptide 1 / 3+**	605.71	32	72	93	139	274	403	527	834	1456	1920	1974	1923	1767
**peptide 1 / 4+**	454.49	10	10	10	10	10	10	10	10	10	10	10	10	10
**antibody+0 pep / 29+**	5049	50	50	50	50	50	50	50	50	50	50	50	50	50
**antibody+0 pep / 28+**	5228	712	757	1275	1481	1476	1642	1507	1004	1397	978	776	686	617
**antibody+0 pep / 27+**	5421	2428	2586	4148	4700	4850	5241	5162	3902	4774	4010	3402	3233	2966
**antibody+0 pep / 26+**	5631	4545	4632	6592	7214	7318	8247	8141	7229	8230	7717	7142	6719	6329
**antibody+0 pep / 25+**	5857	4113	4363	4767	5083	5217	5710	5927	6039	6557	6921	6909	6590	6453
**antibody+0 pep / 24+**	6101	2273	2459	2002	2072	2116	2238	2447	2827	3110	3607	3897	3868	3752
**antibody+0 pep / 23+**	6366	822	907	568	539	517	555	637	824	978	1299	1453	1479	1502
**antibody+0 pep / 22+**	6655	50	50	50	50	50	50	50	50	50	50	50	50	50
**antibody+1 pep / 29+**	5111	50	50	50	50	50	50	50	50	50	50	50	50	50
**antibody+1 pep / 28+**	5296	1334	1355	2283	2624	2570	2847	2590	1786	2166	1502	1209	1080	1027
**antibody+1 pep / 27+**	5488	4020	4056	6240	6899	7042	7619	7386	5842	6596	5537	4845	4499	4180
**antibody+1 pep / 26+**	5702	6064	6285	8246	8909	8867	9701	9631	8948	9331	9095	8585	7920	7471
**antibody+1 pep / 25+**	5930	4471	4683	4967	5135	5278	5686	5789	6031	6262	6772	6805	6426	6181
**antibody+1 pep / 24+**	6176	2073	2196	1826	1807	1944	1956	2044	2464	2607	2973	3316	3149	3045
**antibody+1 pep / 23+**	6443	601	652	425	389	394	410	449	574	657	846	1013	1029	1035
**antibody+1 pep / 22+**	6737	50	50	50	50	50	50	50	50	50	50	50	50	50
**antibody+2 pep / 29+**	5173	50	50	50	50	50	50	50	50	50	50	50	50	50
**antibody+2 pep / 28+**	5356	810	868	1146	1232	1235	1230	1115	758	849	580	483	440	382
**antibody+2 pep / 27+**	5556	2439	2615	2989	3050	3072	2843	2658	2156	2251	1966	1643	1502	1443
**antibody+2 pep / 26+**	5771	3590	3788	3754	3541	3563	3057	3022	2789	2883	2657	2534	2261	2139
**antibody+2 pep / 25+**	6000	2587	2796	2251	2064	2048	1711	1595	1620	1618	1671	1610	1488	1464
**antibody+2 pep / 24+**	6251	1171	1288	823	681	655	520	525	566	564	652	637	613	608
**antibody+2 pep / 23+**	6519	343	375	202	145	149	108	108	141	146	188	180	182	195
**antibody+2 pep / 22+**	6819	50	50	50	50	50	50	50	50	50	50	50	50	50

**antibody fragment**	1200	n.d.	n.d.	n.d.	n.d.	n.d.	n.d.	n.d.	n.d.	n.d.	n.d.	20	20	20
**antibody fragment**	1324	n.d.	n.d.	n.d.	n.d.	n.d.	n.d.	n.d.	20	20	20	29	64	153
**antibody fragment**	1471	n.d.	n.d.	n.d.	n.d.	n.d.	n.d.	n.d.	20	20	20	52	195	457
**antibody fragment**	1554	n.d.	n.d.	n.d.	n.d.	n.d.	n.d.	n.d.	32	43	69	96	n.d.	n.d
**antibody fragment**	1655	n.d.	n.d.	n.d.	n.d.	n.d.	n.d.	n.d.	n.d.	n.d.	n.d.	n.d	242	538
**antibody fragment**	1751	n.d.	n.d.	n.d.	n.d.	n.d.	n.d.	n.d.	38	80	110	171	n.d.	n.d
**antibody fragment**	1851	n.d.	n.d.	n.d.	n.d.	n.d.	n.d.	n.d.	n.d.	n.d	n.d.	n.d	n.d	181
**antibody fragment**	1891	n.d.	n.d.	n.d.	n.d.	n.d.	n.d.	n.d.	n.d.	n.d	n.d.	n.d	n.d	214
**antibody fragment**	1998	n.d.	n.d.	n.d.	n.d.	n.d.	n.d.	n.d.	24	53	99	158	228	20
**antibody fragment**	2006	n.d.	n.d.	n.d.	n.d.	n.d.	n.d.	n.d.	n.d.	n.d.	n.d.	n.d	206	235
**antibody fragment**	2160	n.d.	n.d.	n.d.	n.d.	n.d.	n.d.	n.d.	n.d.	n.d	n.d.	n.d	n.d	20
**antibody fragment**	2189	n.d.	n.d.	n.d.	n.d.	n.d.	n.d.	n.d.	n.d.	n.d.	n.d.	20	20	133
**antibody fragment**	2207	n.d.	n.d.	n.d.	n.d.	n.d.	n.d.	n.d.	n.d.	n.d.	n.d.	20	20	n.d
**antibody fragment**	2331	n.d.	n.d.	n.d.	n.d.	n.d.	n.d.	n.d.	n.d.	n.d	41	n.d.	n.d	141
**antibody fragment**	2508	20	20	20	20	20	20	20	20	20	40	85	150	179
**antibody fragment**	2819	20	20	20	20	20	20	21	20	20	20	n.d.	n.d	124
**antibody fragment**	3336	140	159	147	144	151	155	162	185	180	172	144	128	125
**antibody fragment**	3461	151	176	135	136	130	133	136	152	144	143	129	n.d	20
**antibody fragment**	3590	166	166	168	163	171	173	175	171	165	167	176	181	144
**antibody fragment**	3728	126	135	90	99	91	85	78	74	80	56	65	n.d.	n.d
**antibody fragment**	3886	123	132	84	78	73	64	68	51	62	53	58	59	68
**antibody fragment**	4000	20	20	20	20	20	20	20	20	20	20	20	20	20
**antibody fragment**	4100	20	20	20	20	20	20	20	20	20	20	20	20	20

a) Imputed values necessary for optimized Gauss-fit are shown in red and are equal to the background intensity at the given m/z.
b) n.d.: value not determined / ion signal not present.

Table S3. continued

peptide 1, measurement 3

ion / charge state	m/z	ΔCV												
		**2**	**4**	**6**	**8**	**12**	**16**	**20**	**30**	**40**	**50**	**60**	**70**	**80**
**peptide dimer / 1+**	3628.90	10	10	10	10	10	10	10	10	10	10	10	10	10
**peptide 1 / 1+**	1814.90	6	8	7	7	12	11	12	26	45	63	111	133	152
**peptide 1 / 2+**	907.99	118	187	256	352	692	1634	2155	4871	6635	9454	11135	10995	11326
**peptide 1 / 3+**	605.73	59	76	126	134	238	513	692	1254	1788	2324	2294	2234	2118
**peptide 1 / 4+**	454.49	10	10	10	10	10	10	10	10	10	10	10	10	10
**antibody+0 pep / 29+**	5049	50	50	50	50	50	50	50	50	50	50	50	50	50
**antibody+0 pep / 28+**	5231	506	582	542	400	330	773	652	643	588	587	581	513	507
**antibody+0 pep / 27+**	5424	1963	2302	2188	1773	1616	2834	2646	2631	2519	2622	2564	2315	2218
**antibody+0 pep / 26+**	5632	3689	4083	4114	3696	3484	5073	4974	5110	5317	5485	5376	5124	5021
**antibody+0 pep / 25+**	5860	2851	3293	3291	3274	3274	3893	3959	4544	4877	5184	5348	4993	5039
**antibody+0 pep / 24+**	6102	1315	1404	1517	1659	1685	1712	1835	2330	2667	2971	3142	3031	3131
**antibody+0 pep / 23+**	6365	381	363	404	494	551	496	582	811	1124	1175	1339	1303	1359
**antibody+0 pep / 22+**	6655	50	50	50	50	50	50	50	50	50	50	50	50	50
**antibody+1 pep / 29+**	5111	50	50	50	50	50	50	50	50	50	50	50	50	50
**antibody+1 pep / 28+**	5296	1185	1512	1400	1025	872	1888	1667	1564	1413	1381	1295	1102	1024
**antibody+1 pep / 27+**	5489	4192	4916	4812	4034	3773	6061	5581	5683	5456	5356	5065	4633	4530
**antibody+1 pep / 26+**	5701	6474	7368	7391	7017	6850	8697	8623	9133	9295	9384	9198	8567	8360
**antibody+1 pep / 25+**	5930	4458	4767	5074	5132	5196	5764	5828	6548	7029	7181	7353	6989	7002
**antibody+1 pep / 24+**	6179	1770	1816	2020	2231	2283	2206	2417	3059	3366	3591	3768	3550	3697
**antibody+1 pep / 23+**	6446	388	419	445	524	597	533	608	859	1062	1168	1314	1270	1312
**antibody+1 pep / 22+**	6737	50	50	50	50	50	50	50	50	50	50	50	50	50
**antibody+2 pep / 29+**	5173	50	50	50	50	50	50	50	50	50	50	50	50	50
**antibody+2 pep / 28+**	5359	726	901	842	632	528	1128	1022	968	789	741	669	603	533
**antibody+2 pep / 27+**	5558	2275	2588	2607	2238	2072	3214	2949	2894	2716	2540	2413	2204	2107
**antibody+2 pep / 26+**	5773	3056	3320	3392	3349	3322	3993	3846	3911	4042	3822	3681	3425	3421
**antibody+2 pep / 25+**	6003	1805	1916	2045	2150	2104	2302	2259	2484	2581	2505	2486	2306	2307
**antibody+2 pep / 24+**	6250	618	638	688	753	822	748	800	892	1021	957	1020	905	967
**antibody+2 pep / 23+**	6523	121	129	150	156	167	156	171	195	239	254	271	262	286
**antibody+2 pep / 22+**	6819	50	50	50	50	50	50	50	50	50	50	50	50	50

**antibody fragment**	1000	n.d.	n.d.	n.d.	n.d.	n.d.	n.d.	n.d.	20	20	20	20	20	20
**antibody fragment**	1200	n.d.	n.d.	n.d.	n.d.	n.d.	n.d.	n.d.	20	20	20	20	20	20
**antibody fragment**	1324	n.d.	n.d.	n.d.	n.d.	n.d.	n.d.	n.d.	n.d.	n.d.	n.d.	25	63	125
**antibody fragment**	1471	n.d.	n.d.	n.d.	n.d.	n.d.	n.d.	n.d.	n.d.	n.d.	n.d.	77	182	508
**antibody fragment**	1554	n.d.	n.d.	n.d.	n.d.	n.d.	n.d.	n.d.	46	52	64	n.d.	n.d.	n.d.
**antibody fragment**	1655	n.d.	n.d.	n.d.	n.d.	n.d.	n.d.	n.d.	n.d.	n.d.	n.d.	n.d.	255	576
**antibody fragment**	1751	n.d.	n.d.	n.d.	n.d.	n.d.	n.d.	n.d.	46	75	124	180	n.d.	n.d.
**antibody fragment**	1851	n.d.	n.d.	n.d.	n.d.	n.d.	n.d.	n.d.	n.d.	n.d.	n.d.	n.d.	n.d.	214
**antibody fragment**	1892	n.d.	n.d.	n.d.	n.d.	n.d.	n.d.	n.d.	n.d.	n.d.	n.d.	n.d.	n.d.	256
**antibody fragment**	1998	n.d.	n.d.	n.d.	n.d.	n.d.	n.d.	n.d.	n.d.	54	127	170	227	294
**antibody fragment**	2006	n.d.	n.d.	n.d.	n.d.	n.d.	n.d.	n.d.	n.d.	n.d.	n.d.	n.d.	n.d.	20
**antibody fragment**	2160	n.d.	n.d.	n.d.	n.d.	n.d.	n.d.	n.d.	n.d.	n.d.	n.d.	n.d.	20	20
**antibody fragment**	2189	n.d.	n.d.	n.d.	n.d.	n.d.	n.d.	n.d.	n.d.	n.d.	20	20	20	138
**antibody fragment**	2207	n.d.	n.d.	n.d.	n.d.	n.d.	n.d.	n.d.	n.d.	n.d.	20	20	20	n.d
**antibody fragment**	2331	n.d.	n.d.	n.d.	n.d.	n.d.	n.d.	n.d.	n.d.	n.d.	59	20	n.d	n.d
**antibody fragment**	2508	20	20	20	20	20	20	20	20	20	n.d	97	n.d	240
**antibody fragment**	2819	20	20	20	20	20	20	20	20	20	n.d	n.d	n.d	159
**antibody fragment**	3336	150	139	168	166	189	149	193	193	185	184	157	n.d	n.d
**antibody fragment**	3461	168	176	213	219	218	217	216	235	253	237	205	n.d	n.d
**antibody fragment**	3590	175	169	190	185	213	221	202	232	224	240	265	225	n.d
**antibody fragment**	3728	108	117	129	108	110	145	155	138	132	140	131	113	n.d
**antibody fragment**	3886	62	64	57	65	60	77	80	99	83	91	97	88	n.d
**antibody fragment**	4000	20	20	20	20	20	20	20	20	20	20	20	20	20
**antibody fragment**	4100	20	20	20	20	20	20	20	20	20	20	20	20	20

a) Imputed values necessary for optimized Gauss-fit are shown in red and are equal to the background intensity at the given m/z.
b) n.d.: value not determined / ion signal not present.

Table S3. continued

peptide 1, measurement 4

ion / charge state	m/z	ΔCV												
		**2**	**4**	**6**	**8**	**12**	**16**	**20**	**30**	**40**	**50**	**60**	**70**	**80**
**peptide dimer / 1+**	3628.90	10	10	10	10	10	10	10	10	10	10	10	10	10
**peptide 1 / 1+**	1814.90	5	5	8	8	7	16	8	18	29	58	94	135	156
**peptide 1 / 2+**	907.99	75	111	193	328	465	1052	1709	3463	6093	7789	10328	10711	10635
**peptide 1 / 3+**	605.73	31	52	91	121	153	289	448	799	1373	1513	1621	1573	1376
**peptide 1 / 4+**	454.49	10	10	10	10	10	10	10	10	10	10	10	10	10
**antibody+0 pep / 29+**	5049	50	50	50	50	50	50	50	50	50	50	50	50	50
**antibody+0 pep / 28+**	5244	278	252	294	367	276	377	469	542	636	434	530	522	484
**antibody+0 pep / 27+**	5434	1222	991	1211	1536	897	1356	1608	1870	2164	1790	2002	1957	1785
**antibody+0 pep / 26+**	5642	2507	2038	2481	3083	1928	2504	3042	3327	3720	3465	3906	3782	3400
**antibody+0 pep / 25+**	5866	2259	1846	2289	2727	2017	2299	2567	2826	3119	3222	3550	3472	3149
**antibody+0 pep / 24+**	6111	1122	920	1119	1337	1242	1267	1352	1441	1603	1783	2020	2024	1860
**antibody+0 pep / 23+**	6383	308	262	349	422	418	432	434	526	590	732	812	840	759
**antibody+0 pep / 22+**	6655	50	50	50	50	50	50	50	50	50	50	50	50	50
**antibody+1 pep / 29+**	5111	50	50	50	50	50	50	50	50	50	50	50	50	50
**antibody+1 pep / 28+**	5304	674	604	738	894	627	928	1165	1327	1550	1082	1212	1146	1018
**antibody+1 pep / 27+**	5501	2872	2334	2804	3500	1953	2980	3499	4040	4434	3693	3900	3787	3376
**antibody+1 pep / 26+**	5712	5092	3991	4896	6006	3268	4375	5216	5795	6203	5923	6374	6107	5356
**antibody+1 pep / 25+**	5939	3733	2981	3640	4547	2533	3097	3505	3807	4196	4523	4931	4606	4209
**antibody+1 pep / 24+**	6186	1559	1242	1547	1936	1205	1331	1496	1640	1857	2175	2405	2299	2129
**antibody+1 pep / 23+**	6451	364	310	400	471	341	382	410	492	566	710	807	790	754
**antibody+1 pep / 22+**	6737	50	50	50	50	50	50	50	50	50	50	50	50	50
**antibody+2 pep / 29+**	5173	50	50	50	50	50	50	50	50	50	50	50	50	50
**antibody+2 pep / 28+**	5368	422	445	518	578	559	736	878	934	956	689	671	707	581
**antibody+2 pep / 27+**	5567	1615	1565	1749	2001	1904	2238	2529	2630	2578	2074	2017	1906	1759
**antibody+2 pep / 26+**	5781	2469	2316	2578	3025	2951	3135	3353	3329	3259	2959	2859	2693	2570
**antibody+2 pep / 25+**	6006	1531	1501	1647	2011	2124	2114	2171	2136	2102	2029	1990	1825	1828
**antibody+2 pep / 24+**	6256	545	508	615	729	857	810	853	808	807	903	867	789	848
**antibody+2 pep / 23+**	6523	112	114	128	143	191	189	193	196	235	256	263	268	304
**antibody+2 pep / 22+**	6819	50	50	50	50	50	50	50	50	50	50	50	50	50

**antibody fragment**	1000	n.d.	n.d.	n.d.	n.d.	n.d.	n.d.	n.d.	n.d.	20	20	20	20	20
**antibody fragment**	1200	n.d.	n.d.	n.d.	n.d.	n.d.	n.d.	n.d.	n.d.	20	20	20	20	20
**antibody fragment**	1324	n.d.	n.d.	n.d.	n.d.	n.d.	n.d.	n.d.	n.d.	20	20	n.d.	n.d.	62
**antibody fragment**	1471	n.d.	n.d.	n.d.	n.d.	n.d.	n.d.	n.d.	n.d.	n.d.	n.d.	56	90	205
**antibody fragment**	1554	n.d.	n.d.	n.d.	n.d.	n.d.	n.d.	n.d.	n.d.	37	41	85	n.d.	n.d.
**antibody fragment**	1655	n.d.	n.d.	n.d.	n.d.	n.d.	n.d.	n.d.	n.d.	n.d.	n.d.	n.d.	n.d.	244
**antibody fragment**	1751	n.d.	n.d.	n.d.	n.d.	n.d.	n.d.	n.d.	n.d.	56	77	110	139	n.d.
**antibody fragment**	1892	n.d.	n.d.	n.d.	n.d.	n.d.	n.d.	n.d.	n.d.	n.d.	n.d.	n.d.	n.d.	139
**antibody fragment**	2006	n.d.	n.d.	n.d.	n.d.	n.d.	n.d.	n.d.	n.d.	42	68	102	145	n.d.
**antibody fragment**	2160	n.d.	n.d.	n.d.	n.d.	n.d.	n.d.	n.d.	n.d.	n.d.	n.d.	n.d.	n.d.	20
**antibody fragment**	2189	n.d.	n.d.	n.d.	n.d.	n.d.	n.d.	n.d.	n.d.	n.d.	n.d.	n.d.	n.d.	20
**antibody fragment**	2207	n.d.	n.d.	n.d.	n.d.	n.d.	n.d.	n.d.	n.d.	n.d.	n.d.	20	20	20
**antibody fragment**	2331	20	20	20	20	20	20	20	20	20	20	20	20	20
**antibody fragment**	2508	20	20	20	20	20	20	20	20	20	20	n.d.	n.d.	n.d.
**antibody fragment**	2819	20	20	20	20	20	20	20	20	20	20	n.d.	n.d.	n.d.
**antibody fragment**	3336	147	136	135	166	89	121	132	147	135	115	120	94	89
**antibody fragment**	3461	168	139	172	194	116	146	164	178	177	162	165	133	n.d.
**antibody fragment**	3590	145	131	150	159	121	141	156	168	166	192	187	174	144
**antibody fragment**	3728	88	83	100	96	108	116	125	121	125	125	130	144	122
**antibody fragment**	3886	68	49	53	64	78	99	91	88	90	95	100	97	n.d.
**antibody fragment**	4000	20	20	20	20	20	20	20	20	20	20	20	20	20
**antibody fragment**	4300	20	20	20	20	20	20	20	20	20	20	20	20	20

a) Imputed values necessary for optimized Gauss-fit are shown in red and are equal to the background intensity at the given m/z.
b) n.d.: value not determined / ion signal not present.

Table S3. continued

peptide 1, measurement 5

ion / charge state	m/z	ΔCV												
		**2**	**4**	**6**	**8**	**12**	**16**	**20**	**30**	**40**	**50**	**60**	**70**	**80**
**peptide dimer / 1+**	**3628.90**	10	10	10	10	10	10	10	10	10	10	10	10	10
**peptide 1 / 1+**	**1814.95**	9	12	16	20	20	21	23	70	107	166	285	448	562
**peptide 1 / 2+**	**907.99**	65	84	332	582	1324	2277	3856	7493	13930	23362	31831	35399	36484
**peptide 1 / 3+**	**605.71**	38	95	147	198	414	798	1118	1871	3557	5533	6536	6951	6554
**peptide 1 / 4+**	**454.49**	10	10	10	10	10	10	10	10	10	10	10	10	10
**antibody+0 pep / 28+**	**5244**	50	50	50	50	50	50	50	50	50	50	50	50	50
**antibody+0 pep / 27+**	**5421**	167	228	376	808	978	869	1073	941	1014	983	1062	1207	1113
**antibody+0 pep / 26+**	**5631**	853	1600	2521	4477	5006	4619	5327	5011	5530	5457	5696	6061	5847
**antibody+0 pep / 25+**	**5857**	1944	4170	6678	10015	11048	10343	11170	11011	12011	12296	12995	13408	13027
**antibody+0 pep / 24+**	**6101**	2017	4909	7992	10818	11877	11507	11870	11815	12787	13407	14537	14740	14163
**antibody+0 pep / 23+**	**6366**	1138	2841	4589	6159	6614	6565	6562	6950	7608	8090	9231	9269	9195
**antibody+0 pep / 22+**	**6654**	365	881	1404	1952	2035	2050	2042	2206	2486	3031	3517	3692	3761
**antibody+0 pep / 21+**	**6971**	50	50	50	50	50	50	50	50	50	50	50	50	50
**antibody+1 pep/ 28+**	**5304**	50	50	50	50	50	50	50	50	50	50	50	50	50
**antibody+1 pep/ 27+**	**5488**	480	785	1260	2543	2962	2658	3045	2805	2969	2824	2918	3092	2922
**antibody+1 pep/ 26+**	**5702**	2165	4403	7128	11261	12712	11659	12844	11965	13014	12669	12623	13011	12651
**antibody+1 pep/ 25+**	**5930**	3950	9233	14563	20205	22282	21015	22603	21946	22949	22984	23441	23247	22606
**antibody+1 pep/ 24+**	**6176**	3709	8972	14202	18526	19863	19474	19720	19958	20560	20967	21959	21510	21181
**antibody+1 pep/ 23+**	**6443**	1671	4151	6699	8619	9291	9179	8928	9307	9883	10566	11225	11140	10829
**antibody+1 pep/ 22+**	**6735**	469	1122	1818	2423	2531	2522	2437	2547	2738	3226	3734	3816	3735
**antibody+1 pep/ 21+**	**7057**	50	50	50	50	50	50	50	50	50	50	50	50	50
**antibody+2 peps/ 28+**	**5368**	50	50	50	50	50	50	50	50	50	50	50	50	50
**antibody+2 peps/ 27+**	**5556**	337	620	1052	1845	2064	1924	2130	1891	2053	1819	1804	1873	1803
**antibody+2 peps/ 26+**	**5771**	1311	3055	4735	6737	7430	6773	7498	6910	7281	6639	6484	6511	6102
**antibody+2 peps/ 25+**	**6000**	2125	5282	8138	10549	11626	10771	11179	10969	11033	10510	10345	10032	9511
**antibody+2 peps/ 24+**	**6251**	1646	4176	6437	8016	8631	8342	8249	8300	8236	7948	7790	7390	6981
**antibody+2 peps/ 23+**	**6519**	605	1528	2366	2996	3227	3237	3040	3120	3062	3119	2996	2835	2712
**antibody+2 peps/ 22+**	**6820**	144	363	527	715	775	805	697	744	716	780	849	803	815
**antibody+2 peps/ 21+**	**7143**	50	50	50	50	50	50	50	50	50	50	50	50	50

**antibody fragment**	**1000**	n.d.	n.d.	n.d.	n.d.	n.d.	n.d.	n.d.	n.d.	20	20	20	20	20
**antibody fragment**	**1200**	n.d.	n.d.	n.d.	n.d.	n.d.	n.d.	n.d.	n.d.	20	20	20	20	20
**antibody fragment**	**1471**	n.d.	n.d.	n.d.	n.d.	n.d.	n.d.	n.d.	n.d.	n.d.	53	86	304	640
**antibody fragment**	**1554**	n.d.	n.d.	n.d.	n.d.	n.d.	n.d.	n.d.	n.d.	87	69	111	225	n.d.
**antibody fragment**	**1655**	n.d.	n.d.	n.d.	n.d.	n.d.	n.d.	n.d.	n.d.	n.d.	104	231	728	1794
**antibody fragment**	**1751**	n.d.	n.d.	n.d.	n.d.	n.d.	n.d.	n.d.	n.d.	184	278	493	685	n.d.
**antibody fragment**	**1998**	n.d.	n.d.	n.d.	n.d.	n.d.	n.d.	n.d.	n.d.	186	345	523	834	1135
**antibody fragment**	**2189**	n.d.	n.d.	n.d.	n.d.	n.d.	n.d.	n.d.	n.d.	44	n.d.	n.d.	n.d.	n.d.
**antibody fragment**	**2331**	n.d.	n.d.	n.d.	n.d.	n.d.	n.d.	n.d.	n.d.	n.d.	129	195	351	485
**antibody fragment**	**2558**	n.d.	n.d.	n.d.	n.d.	n.d.	n.d.	n.d.	n.d.	20	20	20	20	20
**antibody fragment**	**2591**	n.d.	n.d.	n.d.	n.d.	n.d.	n.d.	n.d.	n.d.	20	20	20	20	20
**antibody fragment**	**2647**	n.d.	n.d.	n.d.	20	20	20	20	20	20	20	20	20	20
**antibody fragment**	**2819**	n.d.	n.d.	n.d.	20	20	20	20	20	20	n.d.	n.d.	n.d.	n.d.
**antibody fragment**	**3221**	n.d.	n.d.	n.d.	20	20	20	20	20	20	n.d.	n.d.	n.d.	n.d.
**antibody fragment**	**3336**	n.d.	n.d.	n.d.	79	137	164	169	215	186	191	179	151	138
**antibody fragment**	**3461**	n.d.	n.d.	n.d.	171	298	350	316	361	431	399	345	283	175
**antibody fragment**	**3590**	n.d.	n.d.	n.d.	242	345	386	421	450	522	531	501	461	429
**antibody fragment**	**3728**	n.d.	n.d.	n.d.	196	256	286	304	320	319	309	321	276	247
**antibody fragment**	**3886**	n.d.	n.d.	n.d.	131	173	175	192	196	208	226	244	224	247
**antibody fragment**	**4000**	n.d.	n.d.	n.d.	20	20	20	20	20	20	20	20	20	20
**antibody fragment**	**4300**	n.d.	n.d.	n.d.	20	20	20	20	20	20	20	20	20	20

a) Imputed values necessary for optimized Gauss-fit are shown in red and are equal to the background intensity at the given m/z.
b) n.d.: value not determined / ion signal not present.

Table S3. continued

peptide 1, measurement 6

ion / charge state	m/z	ΔCV												
		**2**	**4**	**6**	**8**	**12**	**16**	**20**	**30**	**40**	**50**	**60**	**70**	**80**
**peptide dimer / 1+**	**3628.90**	5	10	10	10	10	10	10	10	10	10	10	10	10
**peptide 1 / 1+**	**1814.95**	5	10	12	13	11	16	14	32	53	100	164	206	369
**peptide 1 / 2+**	**907.99**	50	87	263	384	754	1548	1988	4455	6759	12756	15931	16644	22661
**peptide 1 / 3+**	**605.71**	37	61	112	160	267	458	615	1209	1711	2989	3391	3097	3972
**peptide 1 / 4+**	**454.49**	10	10	10	10	10	10	10	10	10	10	10	10	10
**antibody+0 pep / 28+**	**5244**	50	50	50	50	50	50	50	50	50	50	50	50	50
**antibody+0 pep / 27+**	**5421**	158	188	436	644	570	628	590	662	517	648	657	603	1000
**antibody+0 pep / 26+**	**5631**	862	1266	2479	3400	3119	3236	3036	3319	2714	3502	3467	3276	4741
**antibody+0 pep / 25+**	**5857**	1986	3108	5438	6848	6828	7115	6377	7128	6089	7613	7838	7352	9602
**antibody+0 pep / 24+**	**6101**	2047	3574	5524	6704	6753	7138	6271	6869	6226	7781	8127	7808	9439
**antibody+0 pep / 23+**	**6366**	1069	1882	2778	3211	3579	3792	3185	3636	3585	4472	4989	4994	5535
**antibody+0 pep / 22+**	**6654**	266	512	729	843	925	988	868	1049	1167	1559	1849	1842	2179
**antibody+0 pep / 21+**	**6971**	50	50	50	50	50	50	50	50	50	50	50	50	50
**antibody+1 pep/ 28+**	**5304**	50	50	50	50	50	50	50	50	50	50	50	50	50
**antibody+1 pep/ 27+**	**5488**	471	609	1393	2049	1789	1872	1707	1951	1422	1895	1770	1645	2518
**antibody+1 pep/ 26+**	**5702**	2379	3427	6426	8329	8055	8254	7632	8370	6754	8500	8042	7483	9795
**antibody+1 pep/ 25+**	**5930**	4306	6973	11427	13951	13742	14296	12864	14211	11973	14326	14381	13274	16107
**antibody+1 pep/ 24+**	**6176**	3566	6277	9478	11374	11611	11948	10365	11515	10525	11770	12532	11794	13735
**antibody+1 pep/ 23+**	**6443**	1434	2620	3883	4512	4810	4960	4413	4794	4742	5537	6279	5961	6727
**antibody+1 pep/ 22+**	**6735**	338	604	832	943	1111	1182	1005	1214	1309	1631	1922	1966	2165
**antibody+1 pep/ 21+**	**7057**	50	50	50	50	50	50	50	50	50	50	50	50	50
**antibody+2 peps/ 28+**	**5368**	50	50	50	50	50	50	50	50	50	50	50	50	50
**antibody+2 peps/ 27+**	**5556**	389	497	1039	1418	1284	1309	1219	1345	1109	1211	1137	1069	1528
**antibody+2 peps/ 26+**	**5771**	1519	2295	4058	4960	4655	4774	4526	4892	3909	4477	4167	3839	4836
**antibody+2 peps/ 25+**	**6000**	2396	3925	5962	7129	7062	7162	6670	7001	6015	6304	6216	5700	6602
**antibody+2 peps/ 24+**	**6251**	1656	2764	4098	4734	4934	5046	4481	4918	4260	4429	4547	4049	4548
**antibody+2 peps/ 23+**	**6519**	518	906	1340	1489	1625	1710	1474	1540	1462	1613	1651	1506	1588
**antibody+2 peps/ 22+**	**6820**	108	186	250	286	351	329	307	317	324	391	433	425	474
**antibody+2 peps/ 21+**	**7143**	50	50	50	50	50	50	50	50	50	50	50	50	50

**antibody fragment**	**1000**	n.d.	n.d.	n.d.	n.d.	n.d.	n.d.	n.d.	n.d.	n.d.	20	20	20	20
**antibody fragment**	**1200**	n.d.	n.d.	n.d.	n.d.	n.d.	n.d.	n.d.	n.d.	n.d.	20	20	20	20
**antibody fragment**	**1471**	n.d.	n.d.	n.d.	n.d.	n.d.	n.d.	n.d.	n.d.	n.d.	32	52	120	438
**antibody fragment**	**1554**	n.d.	n.d.	n.d.	n.d.	n.d.	n.d.	n.d.	n.d.	n.d.	n.d.	75	n.d.	n.d.
**antibody fragment**	**1655**	n.d.	n.d.	n.d.	n.d.	n.d.	n.d.	n.d.	n.d.	n.d.	n.d.	151	362	1096
**antibody fragment**	**1751**	n.d.	n.d.	n.d.	n.d.	n.d.	n.d.	n.d.	n.d.	n.d.	168	265	n.d.	n.d.
**antibody fragment**	**1892**	n.d.	n.d.	n.d.	n.d.	n.d.	n.d.	n.d.	n.d.	n.d.	n.d.	n.d.	n.d.	787
**antibody fragment**	**1998**	n.d.	n.d.	n.d.	n.d.	n.d.	n.d.	n.d.	n.d.	n.d.	178	299	405	633
**antibody fragment**	**2189**	n.d.	n.d.	n.d.	n.d.	n.d.	n.d.	n.d.	n.d.	n.d.	n.d.	n.d.	n.d.	313
**antibody fragment**	**2207**	n.d.	n.d.	n.d.	n.d.	n.d.	n.d.	n.d.	n.d.	n.d.	n.d.	n.d.	n.d.	20
**antibody fragment**	**2331**	n.d.	n.d.	n.d.	n.d.	n.d.	n.d.	n.d.	n.d.	n.d.	71	n.d.	n.d.	299
**antibody fragment**	**2508**	n.d.	n.d.	n.d.	n.d.	n.d.	n.d.	n.d.	n.d.	n.d.	n.d.	248	403	515
**antibody fragment**	**2558**	n.d.	n.d.	n.d.	n.d.	n.d.	n.d.	n.d.	n.d.	n.d.	20	n.d.	n.d.	20
**antibody fragment**	**2591**	n.d.	n.d.	n.d.	n.d.	n.d.	n.d.	n.d.	n.d.	n.d.	20	n.d.	n.d.	20
**antibody fragment**	**2647**	n.d.	n.d.	20	20	20	20	20	20	20	20	n.d.	n.d.	20
**antibody fragment**	**2819**	n.d.	n.d.	20	20	20	20	20	20	20	99	133	329	n.d.
**antibody fragment**	**2900**	n.d.	n.d.	n.d.	n.d.	n.d.	n.d.	n.d.	n.d.	n.d.	n.d.	20	20	n.d.
**antibody fragment**	**3000**	n.d.	n.d.	n.d.	n.d.	n.d.	n.d.	n.d.	n.d.	n.d.	n.d.	20	20	n.d.
**antibody fragment**	**3100**	n.d.	n.d.	20	20	20	20	20	20	20	n.d.	20	20	n.d.
**antibody fragment**	**3336**	n.d.	n.d.	n.d.	119	150	141	169	176	192	197	147	137	127
**antibody fragment**	**3461**	n.d.	n.d.	53	235	258	276	305	379	334	333	296	213	175
**antibody fragment**	**3590**	n.d.	n.d.	83	301	359	409	333	406	430	406	439	376	400
**antibody fragment**	**3728**	n.d.	n.d.	80	218	225	259	231	266	273	263	274	236	222
**antibody fragment**	**3886**	n.d.	n.d.	60	148	132	164	135	154	172	186	170	195	n.d.
**antibody fragment**	**4000**	n.d.	n.d.	20	20	20	20	20	20	20	20	20	20	20
**antibody fragment**	**4300**	n.d.	n.d.	20	20	20	20	20	20	20	20	20	20	20

a) Imputed values necessary for optimized Gauss-fit are shown in red and are equal to the background intensity at the given m/z.
b) n.d.: value not determined / ion signal not present.

Table S3. continued

peptide 1, measurement 7

**ion / charge state**	**m/z**	**ΔCV**												
		**2**	**4**	**6**	**8**	**12**	**16**	**20**	**30**	**40**	**50**	**60**	**70**	**80**
**peptide dimer / 1+**	**3628.90**	5	5	5	5	10	10	10	10	10	10	10	10	10
**peptide 1 / 1+**	**1814.95**	6	8	9	9	11	12	10	17	31	65	98	124	163
**peptide 1 / 2+**	**907.99**	81	119	207	278	587	1064	1863	3971	7624	11970	14687	14962	16417
**peptide 1 / 3+**	**605.71**	44	71	116	121	300	477	741	1423	2617	3843	4233	4056	4233
**peptide 1 / 4+**	**454.49**	5	5	5	5	10	10	10	10	10	10	10	10	10
**antibody+0 pep / 28+**	**5244**	50	50	50	50	50	50	50	50	50	50	50	50	50
**antibody+0 pep / 27+**	**5421**	273	523	573	513	563	621	606	704	865	1046	1057	1009	1068
**antibody+0 pep / 26+**	**5631**	782	1491	1600	1495	1653	1689	1822	2285	2717	3165	3332	3165	3362
**antibody+0 pep / 25+**	**5857**	967	1743	1880	1877	2110	2156	2343	2848	3504	4165	4582	4309	4516
**antibody+0 pep / 24+**	**6101**	617	1046	1137	1182	1301	1419	1499	1877	2472	2976	3329	3332	3362
**antibody+0 pep / 23+**	**6366**	225	355	397	433	512	534	624	828	1155	1512	1693	1747	1854
**antibody+0 pep / 22+**	**6654**	52	78	83	86	112	124	164	239	338	494	598	612	664
**antibody+0 pep / 21+**	**6971**	50	50	50	50	50	50	50	50	50	50	50	50	50
**antibody+1 pep/ 28+**	**5304**	50	50	50	50	50	50	50	50	50	50	50	50	50
**antibody+1 pep/ 27+**	**5488**	974	1931	1995	1892	2065	2166	2318	2728	3296	3616	3810	3533	3610
**antibody+1 pep/ 26+**	**5702**	2380	4319	4739	4509	4900	5203	5577	6636	7969	9272	9370	8965	9301
**antibody+1 pep/ 25+**	**5930**	2284	4127	4366	4438	4987	4991	5423	6505	7984	9096	9692	9367	9575
**antibody+1 pep/ 24+**	**6176**	1138	2024	2216	2306	2565	2645	2967	3560	4504	5366	5895	5636	5806
**antibody+1 pep/ 23+**	**6443**	346	568	648	695	796	826	945	1227	1610	2123	2374	2398	2505
**antibody+1 pep/ 22+**	**6735**	65	101	123	131	157	153	190	291	399	591	648	688	762
**antibody+1 pep/ 21+**	**7057**	50	50	50	50	50	50	50	50	50	50	50	50	50
**antibody+2 peps/ 28+**	**5368**	20	50	50	50	50	50	50	50	50	50	50	50	50
**antibody+2 peps/ 27+**	**5556**	978	1753	1826	1723	1898	1956	2103	2423	2793	3052	3039	2890	2978
**antibody+2 peps/ 26+**	**5771**	1833	3273	3480	3357	3656	3803	4131	4774	5516	5913	6088	5718	5752
**antibody+2 peps/ 25+**	**6000**	1504	2424	2662	2680	2927	2944	3228	3811	4284	4665	4778	4453	4540
**antibody+2 peps/ 24+**	**6251**	618	1037	1167	1175	1283	1314	1432	1600	1972	2116	2116	2162	2032
**antibody+2 peps/ 23+**	**6519**	152	245	268	300	340	373	385	428	512	613	642	645	694
**antibody+2 peps/ 22+**	**6820**	31	51	47	55	64	65	72	89	109	142	191	194	215
**antibody+2 peps/ 21+**	**7143**	20	50	50	50	50	50	50	50	50	50	50	50	50

**antibody fragment**	**1000**	n.d.	n.d.	n.d.	n.d.	n.d.	n.d.	n.d.	n.d.	n.d.	n.d.	20	20	20
**antibody fragment**	**1200**	n.d.	n.d.	n.d.	n.d.	n.d.	n.d.	n.d.	n.d.	n.d.	n.d.	20	20	20
**antibody fragment**	**1324**	n.d.	n.d.	n.d.	n.d.	n.d.	n.d.	n.d.	n.d.	20	20	39	68	179
**antibody fragment**	**1471**	n.d.	n.d.	n.d.	n.d.	n.d.	n.d.	n.d.	n.d.	20	20	147	391	1199
**antibody fragment**	**1554**	n.d.	n.d.	n.d.	n.d.	n.d.	n.d.	n.d.	n.d.	90	99	n.d.	n.d.	n.d.
**antibody fragment**	**1655**	n.d.	n.d.	n.d.	n.d.	n.d.	n.d.	n.d.	n.d.	n.d.	n.d.	n.d.	573	1813
**antibody fragment**	**1751**	n.d.	n.d.	n.d.	n.d.	n.d.	n.d.	n.d.	n.d.	137	233	310	n.d.	n.d.
**antibody fragment**	**1892**	n.d.	n.d.	n.d.	n.d.	n.d.	n.d.	n.d.	n.d.	n.d.	n.d.	n.d.	n.d.	827
**antibody fragment**	**1998**	n.d.	n.d.	n.d.	n.d.	n.d.	n.d.	n.d.	n.d.	114	254	409	623	622
**antibody fragment**	**2189**	n.d.	n.d.	n.d.	n.d.	n.d.	n.d.	n.d.	n.d.	61	158	250	410	513
**antibody fragment**	**2331**	n.d.	n.d.	n.d.	n.d.	n.d.	n.d.	n.d.	n.d.	n.d.	84	n.d.	n.d.	n.d.
**antibody fragment**	**2508**	n.d.	n.d.	n.d.	n.d.	n.d.	n.d.	n.d.	n.d.	28	70	102	154	176
**antibody fragment**	**2558**	n.d.	n.d.	n.d.	n.d.	n.d.	n.d.	n.d.	n.d.	20	20	n.d.	n.d.	n.d.
**antibody fragment**	**2591**	n.d.	n.d.	n.d.	n.d.	n.d.	n.d.	n.d.	n.d.	20	20	n.d.	n.d.	n.d.
**antibody fragment**	**2819**	n.d.	n.d.	n.d.	n.d.	n.d.	n.d.	n.d.	n.d.	n.d.	n.d.	40	72	103
**antibody fragment**	**3220**	n.d.	n.d.	n.d.	n.d.	n.d.	n.d.	n.d.	n.d.	n.d.	n.d.	20	20	20
**antibody fragment**	**3336**	n.d.	n.d.	n.d.	n.d.	n.d.	n.d.	n.d.	n.d.	n.d.	n.d.	20	20	20

a) Imputed values necessary for optimized Gauss-fit are shown in red and are equal to the background intensity at the given m/z.
b) n.d.: value not determined / ion signal not present.

Table S4. Ion species, charge states, m/z values and intensities for anti-Troponin I antibody complexed with Troponin I peptide 2 at measured collision cell voltage differences. [a,b)]

peptide 2, measurement 1

ion / charge state	m/z	ΔCV										
		4	8	12	16	20	30	40	50	60	70	80
**peptide dimer / 1+**	3572.80	5	10	10	10	10	10	10	10	10	10	10
**peptide 2 / 1+**	1786.89	8	18	37	74	128	315	544	796	1169	1426	1554
**peptide 2 / 2+**	893.98	73	282	759	1412	2423	5041	8050	11677	16517	17284	17168
**Peptide 2 / 3+**	596.37	8	23	43	63	101	143	212	253	338	277	211
**peptide 2 / 4+**	447.48	5	10	10	10	10	10	10	10	10	10	10
**antibody+0 pep / 27+**	5436	50	50	50	50	50	50	50	50	50	50	50
**antibody+0 pep / 26+**	5643	153	328	432	395	445	421	481	506	554	535	527
**antibody+0 pep / 25+**	5869	478	996	1296	1256	1374	1349	1512	1693	1894	1778	1866
**antibody+0 pep / 24+**	6113	739	1433	1810	1814	1896	2036	2296	2573	2804	2810	2787
**antibody+0 pep / 23+**	6379	521	955	1201	1257	1282	1456	1674	1898	2194	2314	2207
**antibody+0 pep / 22+**	6679	210	321	407	433	433	536	640	726	944	1014	1005
**antibody+0 pep / 21+**	6988	50	50	50	50	50	50	50	50	50	50	50
**antibody+1 pep/ 27+**	5503	50	50	50	50	50	50	50	50	50	50	50
**antibody+1 pep/ 26+**	5712	832	1776	2363	2223	2488	2229	2483	2690	2754	2665	2568
**antibody+1 pep/ 25+**	5940	2081	4230	5694	5571	5981	5806	6410	7072	7353	7177	7080
**antibody+1 pep/ 24+**	6189	2665	5039	6661	6865	7097	7346	8028	8650	9515	9397	9231
**antibody+1 pep/ 23+**	6463	1537	2694	3437	3665	3656	4095	4495	4868	5569	5913	5607
**antibody+1 pep/ 22+**	6760	496	821	954	1071	990	1181	1330	1523	1780	2013	2034
**antibody+1 pep/ 21+**	7075	50	50	50	50	50	50	50	50	50	50	50
**antibody+2 peps/ 27+**	5567	50	50	50	50	50	50	50	50	50	50	50
**antibody+2 peps/ 26+**	5778	1072	2230	3078	2988	3233	2799	2883	2944	2997	2829	2746
**antibody+2 peps/ 25+**	6011	2338	4835	6364	6330	6538	6266	6475	6738	6798	6404	6305
**antibody+2 peps/ 24+**	6261	2604	4868	6394	6533	6565	6573	6781	6770	7148	6889	6658
**antibody+2 peps/ 23+**	6539	1254	2153	2715	2819	2714	2811	2776	2843	2925	3010	2852
**antibody+2 peps/ 22+**	6836	342	550	650	692	625	698	658	652	691	795	737
**antibody+2 peps/ 21+**	7158	50	50	50	50	50	50	50	50	50	50	50

**antibody fragment**	1200	n.d.	n.d.	n.d.	n.d.	n.d.	n.d.	20	20	20	20	20
**antibody fragment**	1324	n.d.	n.d.	n.d.	n.d.	n.d.	n.d.	20	20	20	20	20
**antibody fragment**	1471	n.d.	n.d.	n.d.	n.d.	n.d.	n.d.	n.d.	29	97	292	725
**antibody fragment**	1554	n.d.	n.d.	n.d.	n.d.	n.d.	n.d.	65	70	n.d.	n.d.	n.d.
**antibody fragment**	1655	n.d.	n.d.	n.d.	n.d.	n.d.	n.d.	n.d.	n.d.	180	517	1334
**antibody fragment**	1748	n.d.	n.d.	n.d.	n.d.	n.d.	n.d.	117	220	322	n.d.	n.d.
**antibody fragment**	1851	n.d.	n.d.	n.d.	n.d.	n.d.	n.d.	n.d.	n.d.	n.d.	n.d.	n.d.
**antibody fragment**	1892	n.d.	n.d.	n.d.	n.d.	n.d.	n.d.	n.d.	n.d.	n.d.	n.d.	n.d.
**antibody fragment**	1998	n.d.	n.d.	n.d.	n.d.	n.d.	n.d.	84	195	336	521	621
**antibody fragment**	2160	n.d.	n.d.	n.d.	n.d.	n.d.	n.d.	20	n.d.	n.d.	n.d.	n.d.
**antibody fragment**	2174	n.d.	n.d.	n.d.	n.d.	n.d.	n.d.	20	n.d.	n.d.	n.d.	n.d.
**antibody fragment**	2189	n.d.	n.d.	n.d.	n.d.	n.d.	n.d.	n.d.	n.d.	n.d.	n.d.	197
**antibody fragment**	2331	n.d.	n.d.	n.d.	n.d.	n.d.	n.d.	n.d.	49	100	155	n.d.
**antibody fragment**	2508	n.d.	n.d.	n.d.	n.d.	n.d.	n.d.	n.d.	20	20	46	58
**antibody fragment**	2591	n.d.	n.d.	n.d.	n.d.	n.d.	n.d.	n.d.	20	20	20	20
**antibody fragment**	2819	n.d.	n.d.	n.d.	n.d.	n.d.	n.d.	n.d.	n.d.	n.d.	20	20

a) Imputed values necessary for optimized Gauss-fit are shown in red and are equal to the background intensity at the given m/z.
b) n.d.: value not determined / ion signal not present.

Table S4. continued

peptide 2, measurement 2

**ion / charge state**	**m/z**	**ΔCV**												
		**2**	**4**	**6**	**8**	**12**	**16**	**20**	**30**	**40**	**50**	**60**	**70**	**80**
**peptide dimer / 1+**	3572.80	5	5	5	10	10	10	10	10	10	10	10	10	10
**peptide 2 / 1+**	1786.88	6	7	8	17	27	45	72	149	251	426	161	679	767
**peptide 2 / 2+**	893.93	58	90	145	222	549	868	1277	2585	4382	6437	7312	7553	7546
**peptide 2 / 3+**	596.30	9	7	15	17	41	48	62	91	149	178	589	132	128
**peptide 2 / 4+**	447.48	5	5	5	10	10	10	10	10	10	10	10	10	10
**antibody+0 pep / 29+**	5061	50	50	50	50	50	50	50	50	50	50	50	50	50
**antibody+0 pep / 28+**	5235	129	127	154	179	247	239	228	229	239	249	263	237	229
**antibody+0 pep / 27+**	5427	461	457	461	585	762	781	818	753	868	794	811	785	813
**antibody+0 pep / 26+**	5635	763	779	792	952	1244	1293	1281	1299	1397	1515	1546	1535	1586
**antibody+0 pep / 25+**	5858	578	638	658	690	955	970	993	1052	1201	1409	1481	1528	1532
**antibody+0 pep / 24+**	6103	251	288	286	325	371	399	448	545	671	860	943	958	967
**antibody+0 pep / 23+**	6366	78	83	82	87	103	117	148	210	261	353	406	423	421
**antibody+0 pep / 22+**	6670	50	50	50	50	50	50	50	50	50	50	50	50	50
**antibody+1 pep/ 29+**	5120	50	50	50	50	50	50	50	50	50	50	50	50	50
**antibody+1 pep/ 28+**	5299	761	749	763	926	1272	1293	1308	1263	1271	1155	1113	1025	1072
**antibody+1 pep/ 27+**	5493	2220	2373	2518	2959	3739	3640	3770	3668	3787	3727	3625	3498	3457
**antibody+1 pep/ 26+**	5703	3233	3354	3483	3915	4653	4720	4981	5077	5329	5664	5610	5615	5530
**antibody+1 pep/ 25+**	5931	1987	2153	2251	2311	2629	2736	2919	3193	3559	3964	4224	4252	4261
**antibody+1 pep/ 24+**	6176	743	771	825	823	896	965	1145	1334	1590	1965	2166	2192	2188
**antibody+1 pep/ 23+**	6447	160	165	181	178	187	235	280	364	492	619	724	749	747
**antibody+1 pep/ 22+**	6748	50	50	50	50	50	50	50	50	50	50	50	50	50
**antibody+2 peps/ 29+**	5176	50	50	50	50	50	50	50	50	50	50	50	50	50
**antibody+2 peps/ 28+**	5360	991	1036	1052	1257	1761	1770	1675	1682	1571	1363	1252	1143	1154
**antibody+2 peps/ 27+**	5558	2767	2915	2998	3432	4397	4374	4404	4314	4183	3891	3604	3468	3315
**antibody+2 peps/ 26+**	5771	3409	3685	3687	4053	4807	4816	4936	4838	4804	4816	4696	4500	4369
**antibody+2 peps/ 25+**	5999	1841	1935	2010	2150	2311	2356	2533	2514	2618	2673	2737	2632	2510
**antibody+2 peps/ 24+**	6251	582	601	622	638	650	681	711	800	856	843	884	884	846
**antibody+2 peps/ 23+**	6519	100	111	118	107	107	124	121	143	172	194	200	215	247
**antibody+2 peps/ 22+**	6823	50	50	50	50	50	50	50	50	50	50	50	50	50

**antibody fragment**	1000	n.d.	n.d.	n.d.	n.d.	n.d.	n.d.	n.d.	n.d.	n.d.	20	20	20	20
**antibody fragment**	1324	n.d.	n.d.	n.d.	n.d.	n.d.	n.d.	n.d.	n.d.	n.d.	n.d.	33	44	118
**antibody fragment**	1471	n.d.	n.d.	n.d.	n.d.	n.d.	n.d.	n.d.	n.d.	n.d.	35	51	151	363
**antibody fragment**	1554	n.d.	n.d.	n.d.	n.d.	n.d.	n.d.	n.d.	n.d.	n.d.	54	79	n.d.	n.d.
**antibody fragment**	1655	n.d.	n.d.	n.d.	n.d.	n.d.	n.d.	n.d.	n.d.	n.d.	n.d.	n.d.	n.d.	365
**antibody fragment**	1751	n.d.	n.d.	n.d.	n.d.	n.d.	n.d.	n.d.	n.d.	n.d.	67	125	127	n.d.
**antibody fragment**	1892	n.d.	n.d.	n.d.	n.d.	n.d.	n.d.	n.d.	n.d.	n.d.	n.d.	n.d.	73	172
**antibody fragment**	1998	n.d.	n.d.	n.d.	n.d.	n.d.	n.d.	n.d.	n.d.	n.d.	n.d.	n.d.	n.d.	181
**antibody fragment**	2006	n.d.	n.d.	n.d.	n.d.	n.d.	n.d.	n.d.	n.d.	n.d.	59	105	n.d.	n.d.
**antibody fragment**	2160	n.d.	n.d.	n.d.	n.d.	n.d.	n.d.	n.d.	n.d.	n.d.	20	20	20	20
**antibody fragment**	2207	n.d.	n.d.	n.d.	n.d.	n.d.	n.d.	n.d.	n.d.	n.d.	20	20	20	20
**antibody fragment**	2508	20	20	20	20	20	20	20	20	20	20	66	n.d.	n.d.
**antibody fragment**	2819	20	20	20	20	20	20	20	20	20	20	n.d.	53	103
**antibody fragment**	3336	69	82	102	72	90	83	92	94	100	104	n.d.	n.d.	n.d.
**antibody fragment**	3461	176	164	171	181	188	207	215	209	227	204	176	146	n.d.
**antibody fragment**	3590	n.d.	n.d.	n.d.	n.d.	n.d.	n.d.	n.d.	n.d.	n.d.	n.d.	n.d.	162	144
**antibody fragment**	3728	89	86	93	90	112	117	125	132	119	139	147	111	87
**antibody fragment**	3886	44	44	44	46	68	67	68	73	80	75	68	72	82
**antibody fragment**	4200	20	20	20	20	20	20	20	20	20	20	20	20	20
**antibody fragment**	4400	20	20	20	20	20	20	20	20	20	20	20	20	20

a) Imputed values necessary for optimized Gauss-fit are shown in red and are equal to the background intensity at the given m/z.
b) n.d.: value not determined / ion signal not present.

Table S4. continued

peptide 2, measurement 3

ion / charge state	m/z	ΔCV												
		**2**	**4**	**6**	**8**	**12**	**16**	**20**	**30**	**40**	**50**	**60**	**70**	**80**
**peptide dimer / 1+**	3572.80	5	10	10	10	10	10	10	10	10	10	10	10	10
**peptide 2 / 1+**	1786.90	5	11	19	26	74	143	238	493	701	1352	1862	1944	2023
**peptide 2 / 2+**	893.95	80	131	381	688	1366	2225	3671	5541	7831	13860	15749	14776	14302
**peptide 2 / 3+**	596.30	11	27	29	39	70	94	137	163	207	320	336	239	201
**peptide 2 / 4+**	447.48	5	10	10	10	10	10	10	10	10	10	10	10	10
**antibody+0 pep / 28+**	5235	50	50	50	50	50	50	50	50	50	50	50	50	50
**antibody+0 pep / 27+**	5437	157	317	452	808	901	825	934	739	599	813	809	636	600
**antibody+0 pep / 26+**	5647	700	1269	1855	2888	3158	2980	3205	2703	2314	3161	2958	2488	2322
**antibody+0 pep / 25+**	5868	1168	2206	3109	4355	4561	4355	4704	4354	3912	5118	5049	4524	4278
**antibody+0 pep / 24+**	6115	950	1688	2513	3355	3383	3276	3468	3580	3573	4489	4560	4379	4087
**antibody+0 pep / 23+**	6373	417	725	1031	1347	1397	1388	1464	1654	1811	2332	2622	2443	2326
**antibody+0 pep / 22+**	6654	103	175	252	311	339	353	380	490	585	830	899	942	886
**antibody+0 pep / 21+**	6984	50	50	50	50	50	50	50	50	50	50	50	50	50
**antibody+1 pep/ 28+**	5299	50	50	50	50	50	50	50	50	50	50	50	50	50
**antibody+1 pep/ 27+**	5504	823	1396	2129	3804	4071	3770	4165	3194	2660	3647	3199	2573	2355
**antibody+1 pep/ 26+**	5712	2575	4841	7106	10342	10903	10664	11269	9644	8621	10628	10095	8604	8102
**antibody+1 pep/ 25+**	5938	3433	6573	9293	12789	13266	12888	13550	12690	11832	14229	13710	12540	11888
**antibody+1 pep/ 24+**	6180	2367	4411	6277	8211	8285	8309	8337	8524	8699	10190	10530	9974	9335
**antibody+1 pep/ 23+**	6449	810	1540	2156	2765	2743	2767	2805	3177	3506	4141	4713	4568	4427
**antibody+1 pep/ 22+**	6737	175	310	433	556	556	557	588	747	944	1142	1307	1417	1339
**antibody+1 pep/ 21+**	7066	50	50	50	50	50	50	50	50	50	50	50	50	50
**antibody+2 peps/ 28+**	5360	50	50	50	50	50	50	50	50	50	50	50	50	50
**antibody+2 peps/ 27+**	5566	923	1713	2498	3872	4215	3858	4253	3305	2848	3393	3008	2392	2220
**antibody+2 peps/ 26+**	5774	2411	4633	6678	9027	9535	9079	9527	8345	7563	8498	7887	6804	6327
**antibody+2 peps/ 25+**	5999	2779	5338	7468	9519	9733	9588	9750	9069	8832	9437	9113	8395	7612
**antibody+2 peps/ 24+**	6246	1572	2915	4076	5117	5260	5127	5019	4998	5081	5260	5248	4969	4664
**antibody+2 peps/ 23+**	6521	456	813	1149	1432	1431	1394	1367	1424	1540	1619	1641	1636	1524
**antibody+2 peps/ 22+**	6817	83	147	192	239	259	254	239	294	314	375	415	453	447
**antibody+2 peps/ 21+**	7145	50	50	50	50	50	50	50	50	50	50	50	50	50

**antibody fragment**	1000	n.d.	n.d.	n.d.	n.d.	n.d.	n.d.	n.d.	n.d.	n.d.	n.d.	n.d.	20	20
**antibody fragment**	1200	n.d.	n.d.	n.d.	n.d.	n.d.	n.d.	n.d.	n.d.	n.d.	n.d.	n.d.	20	20
**antibody fragment**	1324	n.d.	n.d.	n.d.	n.d.	n.d.	n.d.	n.d.	n.d.	20	20	20	68	91
**antibody fragment**	1440	n.d.	n.d.	n.d.	n.d.	n.d.	n.d.	n.d.	n.d.	20	20	20	n.d.	n.d.
**antibody fragment**	1471	n.d.	n.d.	n.d.	n.d.	n.d.	n.d.	n.d.	n.d.	n.d.	56	154	291	637
**antibody fragment**	1554	n.d.	n.d.	n.d.	n.d.	n.d.	n.d.	n.d.	n.d.	72	83	118	n.d.	n.d.
**antibody fragment**	1655	n.d.	n.d.	n.d.	n.d.	n.d.	n.d.	n.d.	n.d.	n.d.	87	211	580	1475
**antibody fragment**	1751	n.d.	n.d.	n.d.	n.d.	n.d.	n.d.	n.d.	n.d.	135	218	288	n.d.	n.d.
**antibody fragment**	1892	n.d.	n.d.	n.d.	n.d.	n.d.	n.d.	n.d.	n.d.	n.d.	n.d.	n.d.	n.d.	805
**antibody fragment**	1998	n.d.	n.d.	n.d.	n.d.	n.d.	n.d.	n.d.	n.d.	132	260	388	469	538
**antibody fragment**	2160	n.d.	n.d.	n.d.	n.d.	n.d.	n.d.	n.d.	n.d.	43	n.d.	n.d.	181	230
**antibody fragment**	2189	n.d.	n.d.	n.d.	n.d.	n.d.	n.d.	n.d.	n.d.	n.d.	92	n.d.	n.d.	289
**antibody fragment**	2207	n.d.	n.d.	n.d.	n.d.	n.d.	n.d.	n.d.	n.d.	20	20	n.d.	n.d.	186
**antibody fragment**	2331	n.d.	n.d.	n.d.	n.d.	n.d.	n.d.	n.d.	n.d.	20	20	170	n.d.	248
**antibody fragment**	2559	n.d.	n.d.	n.d.	n.d.	n.d.	n.d.	n.d.	n.d.	n.d.	n.d.	20	20	20
**antibody fragment**	2591	n.d.	n.d.	n.d.	n.d.	n.d.	n.d.	n.d.	n.d.	n.d.	n.d.	20	20	20
**antibody fragment**	2647	n.d.	n.d.	n.d.	20	20	20	20	20	20	20	20	20	20
**antibody fragment**	2819	n.d.	n.d.	n.d.	20	20	20	20	20	20	20	n.d.	n.d.	n.d.
**antibody fragment**	3336	n.d.	n.d.	n.d.	96	132	132	167	161	165	172	148	110	128
**antibody fragment**	3461	n.d.	n.d.	n.d.	254	315	339	333	387	396	464	383	232	n.d.
**antibody fragment**	3590	n.d.	n.d.	n.d.	n.d.	n.d.	n.d.	n.d.	n.d.	n.d.	n.d.	n.d.	379	387
**antibody fragment**	3728	n.d.	n.d.	n.d.	210	241	233	282	252	309	297	325	268	n.d.
**antibody fragment**	3886	n.d.	n.d.	n.d.	103	109	135	130	138	141	161	189	179	224
**antibody fragment**	4000	n.d.	n.d.	n.d.	20	20	20	20	20	20	20	20	20	20
**antibody fragment**	4200	n.d.	n.d.	n.d.	20	20	20	20	20	20	20	20	20	20

a) Imputed values necessary for optimized Gauss-fit are shown in red and are equal to the background intensity at the given m/z.
b) n.d.: value not determined / ion signal not present.

Table S4. continued

peptide 2, measurement 4

ion / charge state	m/z	ΔCV												
		**2**	**4**	**6**	**8**	**12**	**16**	**20**	**30**	**40**	**50**	**60**	**70**	**80**
**peptide dimer / 1+**	3572.80	5	10	10	10	10	10	10	10	10	10	10	10	10
**peptide 2 / 1+**	1786.90	7	14	13	17	30	55	118	224	363	635	762	1029	1055
**peptide 2 / 2+**	893.95	123	272	406	650	1219	2121	3215	5193	7238	11203	13397	15021	14756
**peptide 2 / 3+**	596.30	15	32	33	55	95	140	185	235	312	405	363	356	272
**peptide 2 / 4+**	447.48	5	10	10	10	10	10	10	10	10	10	10	10	10
**antibody+0 pep / 29+**	5058	50	50	50	50	50	50	50	50	50	50	50	50	50
**antibody+0 pep / 28+**	5235	66	110	153	170	188	192	205	195	178	208	219	251	250
**antibody+0 pep / 27+**	5437	426	714	886	1039	1147	1088	1133	1099	1007	1190	1278	1407	1362
**antibody+0 pep / 26+**	5647	1158	2012	2300	2554	2768	2773	2909	2903	2714	3235	3623	4003	3784
**antibody+0 pep / 25+**	5868	1338	2256	2502	2827	3059	3142	3164	3258	3260	4058	4446	4842	4763
**antibody+0 pep / 24+**	6115	745	1304	1400	1534	1660	1748	1817	2014	2150	2752	3071	3375	3294
**antibody+0 pep / 23+**	6373	246	408	441	500	551	598	667	780	965	1269	1478	1577	1564
**antibody+0 pep / 22+**	6668	50	50	50	50	50	50	50	50	50	50	50	50	50
**antibody+1 pep/ 29+**	5117	50	50	50	50	50	50	50	50	50	50	50	50	50
**antibody+1 pep/ 28+**	5292	264	514	630	729	815	832	842	772	657	750	836	882	826
**antibody+1 pep/ 27+**	5504	1760	3097	3623	4164	4364	4256	4497	4184	3777	4339	4706	4931	4769
**antibody+1 pep/ 26+**	5712	3888	6770	7695	8525	8939	8992	9252	8973	8511	9964	10642	11293	10839
**antibody+1 pep/ 25+**	5938	3472	5967	6625	7683	7710	7924	8009	8099	8122	9556	10284	10999	10726
**antibody+1 pep/ 24+**	6180	1635	2669	3003	3344	3590	3677	3781	3975	4271	5244	5739	6214	6041
**antibody+1 pep/ 23+**	6449	426	669	766	887	897	979	1006	1197	1324	1824	2048	2305	2224
**antibody+1 pep/ 22+**	6745	50	50	50	50	50	50	50	50	50	50	50	50	50
**antibody+2 peps/ 29+**	5173	50	50	50	50	50	50	50	50	50	50	50	50	50
**antibody+2 peps/ 28+**	5355	317	624	759	808	868	882	865	809	710	766	782	858	796
**antibody+2 peps/ 27+**	5566	1759	3098	3609	4003	4115	4116	4147	3849	3376	3856	3866	4267	3972
**antibody+2 peps/ 26+**	5774	3256	5645	6344	7109	7069	7048	7227	6781	6343	6980	7169	7690	7266
**antibody+2 peps/ 25+**	5999	2286	3837	4249	4852	4901	4982	4930	4878	4552	5129	5288	5553	5194
**antibody+2 peps/ 24+**	6246	878	1408	1604	1732	1780	1860	1827	1820	1804	2054	2177	2264	2123
**antibody+2 peps/ 23+**	6521	184	289	305	363	363	418	386	411	427	510	567	603	584

**antibody+2 peps/ 22+**	6820	50	50	50	50	50	50	50	50	50	50	50	50	50
**antibody fragment**	1000	n.d.	n.d.	n.d.	n.d.	n.d.	n.d.	n.d.	n.d.	n.d.	n.d.	n.d	20	20
**antibody fragment**	1200	n.d.	n.d.	n.d.	n.d.	n.d.	n.d.	n.d.	n.d.	n.d.	n.d.	20	20	20
**antibody fragment**	1324	n.d.	n.d.	n.d.	n.d.	n.d.	n.d.	n.d.	n.d.	n.d.	n.d.	20	75	145
**antibody fragment**	1440	n.d.	n.d.	n.d.	n.d.	n.d.	n.d.	n.d.	n.d.	20	20	n.d.	n.d.	n.d.
**antibody fragment**	1471	n.d.	n.d.	n.d.	n.d.	n.d.	n.d.	n.d.	n.d.	20	20	112	454	1137
**antibody fragment**	1554	n.d.	n.d.	n.d.	n.d.	n.d.	n.d.	n.d.	n.d.	79	85	n.d.	n.d.	n.d.
**antibody fragment**	1655	n.d.	n.d.	n.d.	n.d.	n.d.	n.d.	n.d.	n.d.	n.d.	n.d.	165	704	1784
**antibody fragment**	1751	n.d.	n.d.	n.d.	n.d.	n.d.	n.d.	n.d.	n.d.	141	194	268	n.d.	n.d.
**antibody fragment**	1892	n.d.	n.d.	n.d.	n.d.	n.d.	n.d.	n.d.	n.d.	n.d.	n.d.	n.d.	n.d.	808
**antibody fragment**	1998	n.d.	n.d.	n.d.	n.d.	n.d.	n.d.	n.d.	n.d.	109	281	353	547	554
**antibody fragment**	2189	n.d.	n.d.	n.d.	n.d.	n.d.	n.d.	n.d.	n.d.	50	81	142	230	315
**antibody fragment**	2207	n.d.	n.d.	n.d.	n.d.	n.d.	n.d.	n.d.	n.d.	20	n.d.	n.d.	n.d.	n.d.
**antibody fragment**	2331	n.d.	n.d.	n.d.	n.d.	n.d.	n.d.	n.d.	n.d.	20	n.d.	n.d.	n.d.	n.d.
**antibody fragment**	2508	n.d.	n.d.	n.d.	n.d.	n.d.	n.d.	n.d.	n.d.	n.d.	55	97	176	203
**antibody fragment**	2559	n.d.	n.d.	n.d.	n.d.	n.d.	n.d.	n.d.	n.d.	n.d.	20	20	n.d.	n.d.
**antibody fragment**	2591	n.d.	n.d.	n.d.	n.d.	n.d.	n.d.	n.d.	n.d.	n.d.	20	20	n.d.	n.d.
**antibody fragment**	2819	n.d.	n.d.	n.d.	n.d.	n.d.	n.d.	n.d.	n.d.	n.d.	n.d.	n.d.	77	131
**antibody fragment**	3336	n.d.	n.d.	n.d.	n.d.	n.d.	n.d.	n.d.	n.d.	n.d.	n.d.	n.d.	20	20
**antibody fragment**	3461	n.d.	n.d.	n.d.	n.d.	n.d.	n.d.	n.d.	n.d.	n.d.	n.d.	n.d.	20	20

a) Imputed values necessary for optimized Gauss-fit are shown in red and are equal to the background intensity at the given m/z.
b) n.d.: value not determined / ion signal not present.

Table S4. continued

peptide 2, measurement 5

ion / charge state	m/z	ΔCV												
		**2**	**4**	**6**	**8**	**12**	**16**	**20**	**30**	**40**	**50**	**60**	**70**	**80**
**peptide dimer / 1+**	3572.80	5	10	10	10	10	10	10	10	10	10	10	10	10
**peptide 2 / 1+**	1786.90	6	16	19	17	45	78	150	326	515	689	911	1030	1107
**peptide 2 / 2+**	893.95	114	274	570	750	1467	2563	3902	7204	10707	13126	15382	15624	15892
**peptide 2 / 3+**	596.30	20	42	47	60	99	165	216	309	418	455	438	366	308
**peptide 2 / 4+**	447.48	5	10	10	10	10	10	10	10	10	10	10	10	10
**antibody+0 pep / 29+**	5058	50	50	50	50	50	50	50	50	50	50	50	50	50
**antibody+0 pep / 28+**	5235	65	121	172	183	213	210	233	249	244	251	246	243	254
**antibody+0 pep / 27+**	5437	407	774	920	1041	1154	1272	1323	1374	1378	1430	1456	1423	1381
**antibody+0 pep / 26+**	5647	1164	2143	2417	2675	2927	3112	3223	3528	3572	3840	3925	3955	3935
**antibody+0 pep / 25+**	5868	1450	2334	2769	3030	3164	3382	3516	3984	4341	4702	4908	4923	4893
**antibody+0 pep / 24+**	6115	812	1291	1497	1649	1736	1924	1978	2455	2749	3064	3318	3439	3411
**antibody+0 pep / 23+**	6373	263	414	477	510	570	643	709	942	1152	1420	1576	1592	1605
**antibody+0 pep / 22+**	6668	50	50	50	50	50	50	50	50	50	50	50	50	50
**antibody+1 pep/ 29+**	5117	50	50	50	50	50	50	50	50	50	50	50	50	50
**antibody+1 pep/ 28+**	5292	295	515	716	783	891	934	943	1012	946	958	945	910	909
**antibody+1 pep/ 27+**	5504	1755	3213	3865	4194	4589	4859	4900	5192	5120	5187	5105	5020	4901
**antibody+1 pep/ 26+**	5712	4122	6995	8168	8866	9568	10034	10257	10947	11083	11385	11484	11495	11383
**antibody+1 pep/ 25+**	5938	3706	6228	7289	7662	8251	8700	8862	9633	10285	10803	11242	11192	11117
**antibody+1 pep/ 24+**	6180	1775	2866	3235	3604	3821	3992	4120	4650	5305	5862	6178	6254	6367
**antibody+1 pep/ 23+**	6449	462	729	808	877	969	1112	1139	1368	1651	1951	2148	2201	2217
**antibody+1 pep/ 22+**	6745	50	50	50	50	50	50	50	50	50	50	50	50	50
**antibody+2 peps/ 29+**	5173	50	50	50	50	50	50	50	50	50	50	50	50	50
**antibody+2 peps/ 28+**	5355	327	630	756	854	933	1014	974	1024	975	877	871	875	815
**antibody+2 peps/ 27+**	5566	1844	3265	3705	4020	4375	4612	4587	4695	4548	4337	4333	4106	4076
**antibody+2 peps/ 26+**	5774	3391	5881	6669	7243	7650	7953	7890	8164	8094	7912	7804	7626	7592
**antibody+2 peps/ 25+**	5999	2537	4146	4691	5065	5305	5424	5391	5593	5670	5615	5643	5516	5354
**antibody+2 peps/ 24+**	6246	964	1529	1750	1865	2031	2009	2053	2073	2155	2154	2273	2169	2254
**antibody+2 peps/ 23+**	6521	204	292	326	385	404	430	433	451	482	521	562	582	579

**antibody+2 peps/ 22+**	6820	50	50	50	50	50	50	50	50	50	50	50	50	50
**antibody fragment**	1000	n.d.	n.d.	n.d.	n.d.	n.d.	n.d.	n.d.	n.d.	n.d.	n.d.	n.d.	20	20
**antibody fragment**	1200	n.d.	n.d.	n.d.	n.d.	n.d.	n.d.	n.d.	n.d.	n.d.	n.d.	n.d.	20	20
**antibody fragment**	1324	n.d.	n.d.	n.d.	n.d.	n.d.	n.d.	n.d.	n.d.	n.d.	20	20	73	154
**antibody fragment**	1440	n.d.	n.d.	n.d.	n.d.	n.d.	n.d.	n.d.	n.d.	20	20	20	n.d.	n.d.
**antibody fragment**	1471	n.d.	n.d.	n.d.	n.d.	n.d.	n.d.	n.d.	n.d.	20	64	163	365	1112
**antibody fragment**	1554	n.d.	n.d.	n.d.	n.d.	n.d.	n.d.	n.d.	n.d.	106	n.d.	n.d.	n.d.	n.d.
**antibody fragment**	1655	n.d.	n.d.	n.d.	n.d.	n.d.	n.d.	n.d.	n.d.	n.d.	n.d.	n.d.	688	1821
**antibody fragment**	1751	n.d.	n.d.	n.d.	n.d.	n.d.	n.d.	n.d.	n.d.	183	207	274	n.d.	n.d.
**antibody fragment**	1892	n.d.	n.d.	n.d.	n.d.	n.d.	n.d.	n.d.	n.d.	n.d.	n.d.	n.d.	n.d.	799
**antibody fragment**	1998	n.d.	n.d.	n.d.	n.d.	n.d.	n.d.	n.d.	n.d.	144	255	365	521	496
**antibody fragment**	2189	n.d.	n.d.	n.d.	n.d.	n.d.	n.d.	n.d.	n.d.	43	n.d.	n.d.	206	289
**antibody fragment**	2207	n.d.	n.d.	n.d.	n.d.	n.d.	n.d.	n.d.	n.d.	20	n.d.	n.d.	n.d.	n.d.
**antibody fragment**	2331	n.d.	n.d.	n.d.	n.d.	n.d.	n.d.	n.d.	n.d.	20	100	130	n.d.	n.d.
**antibody fragment**	2508	n.d.	n.d.	n.d.	n.d.	n.d.	n.d.	n.d.	n.d.	n.d.	46	116	185	240
**antibody fragment**	2819	n.d.	n.d.	n.d.	n.d.	n.d.	n.d.	n.d.	n.d.	n.d.	34	51	71	121
**antibody fragment**	3336	n.d.	n.d.	n.d.	n.d.	n.d.	n.d.	n.d.	n.d.	n.d.	20	20	20	20
**antibody fragment**	3461	n.d.	n.d.	n.d.	n.d.	n.d.	n.d.	n.d.	n.d.	n.d.	20	20	20	20

a) Imputed values necessary for optimized Gauss-fit are shown in red and are equal to the background intensity at the given m/z.
b) n.d.: value not determined / ion signal not present.

Table S5. Ion species, charge states, m/z values and intensities for anti-Troponin I antibody complexed with Troponin I peptide 3 at measured collision cell voltage differences. [a,b)]

peptide 3, measurement 1

**ion / charge state**	**m/z**	**ΔCV**												
		**2**	**4**	**6**	**8**	**12**	**16**	**20**	**30**	**40**	**50**	**60**	**70**	**80**
**peptide dimer / 1+**	3590.78	10	10	10	10	10	10	10	10	10	10	10	10	10
**peptide 4 / 1+**	1795.89	10	11	11	18	13	10	10	17	25	51	61	84	86
**peptide 4 / 2+**	898.47	136	222	478	612	772	740	827	1375	1784	3386	3942	3972	3655
**peptide 4 / 3+**	599.34	19	39	54	79	92	78	92	112	149	266	276	230	177
**peptide 4 / 4+**	449.72	10	10	10	10	10	10	10	10	10	10	10	10	10
**antibody+0 pep / 29+**	5055	50	50	50	50	50	50	50	50	50	50	50	50	50
**antibody+0 pep / 28+**	5234	176	234	465	419	451	291	253	241	203	252	264	242	215
**antibody+0 pep / 27+**	5430	1346	1883	2820	2804	2587	1835	1475	1386	1284	1706	1711	1647	1401
**antibody+0 pep / 26+**	5638	4288	6003	7545	7853	6437	4890	3966	3591	3609	4756	4999	4899	4521
**antibody+0 pep / 25+**	5864	5466	7679	8600	9428	7191	5621	4432	3961	4169	5997	6240	6168	5846
**antibody+0 pep / 24+**	6108	3413	4643	4898	5579	3889	3231	2552	2279	2597	3865	4195	4240	3851
**antibody+0 pep / 23+**	6378	1187	1557	1604	1867	1252	1019	867	774	943	1508	1613	1709	1632
**antibody+0 pep / 22+**	6664	50	50	50	50	50	50	50	50	50	50	50	50	50
**antibody+1 pep/ 29+**	5119	50	50	50	50	50	50	50	50	50	50	50	50	50
**antibody+1 pep/ 28+**	5300	255	308	596	513	534	355	279	295	237	288	300	294	265
**antibody+1 pep/ 27+**	5497	1577	2218	3049	2977	2731	1978	1537	1374	1336	1666	1658	1664	1440
**antibody+1 pep/ 26+**	5708	4174	5908	7131	7382	5882	4315	3477	3009	3026	4068	4035	4168	3616
**antibody+1 pep/ 25+**	5937	4511	6242	6823	7387	5269	4107	3241	2775	2900	4098	4212	4289	3935
**antibody+1 pep/ 24+**	6185	2386	3255	3409	3758	2574	2094	1605	1441	1567	2284	2322	2393	2262
**antibody+1 pep/ 23+**	6459	704	894	923	1038	712	578	454	411	487	734	825	845	776
**antibody+1 pep/ 22+**	6747	50	50	50	50	50	50	50	50	50	50	50	50	50
**antibody+2 peps/ 29+**	5178	50	50	50	50	50	50	50	50	50	50	50	50	50
**antibody+2 peps/ 28+**	5364	97	114	214	187	194	116	118	102	86	101	96	104	92
**antibody+2 peps/ 27+**	5555	462	659	909	891	772	575	432	360	361	445	416	437	364
**antibody+2 peps/ 26+**	5778	1118	1473	1789	1860	1416	1001	797	729	685	887	852	858	798
**antibody+2 peps/ 25+**	6009	988	1337	1520	1595	1164	850	682	594	591	761	771	745	667
**antibody+2 peps/ 24+**	6256	476	621	657	737	518	389	322	301	292	378	429	394	336

**antibody+2 peps/ 23+**	6530	130	158	157	188	130	111	89	93	97	142	140	153	133
**antibody+2 peps/ 22+**	6825	50	50	50	50	50	50	50	50	50	50	50	50	50
**antibody fragment**	1000	n.d.	n.d.	n.d.	n.d.	n.d.	n.d.	n.d.	n.d.	n.d.	n.d.	n.d.	20	20
**antibody fragment**	1200	n.d.	n.d.	n.d.	n.d.	n.d.	n.d.	n.d.	n.d.	n.d.	n.d.	20	20	20
**antibody fragment**	1324	n.d.	n.d.	n.d.	n.d.	n.d.	n.d.	n.d.	n.d.	20	20	20	27	28
**antibody fragment**	1471	n.d.	n.d.	n.d.	n.d.	n.d.	n.d.	n.d.	n.d.	n.d.	20	43	106	190
**antibody fragment**	1554	n.d.	n.d.	n.d.	n.d.	n.d.	n.d.	n.d.	n.d.	39	n.d.	n.d.	n.d.	n.d.
**antibody fragment**	1655	n.d.	n.d.	n.d.	n.d.	n.d.	n.d.	n.d.	n.d.	n.d.	n.d.	75	147	363
**antibody fragment**	1751	n.d.	n.d.	n.d.	n.d.	n.d.	n.d.	n.d.	n.d.	48	76	n.d.	n.d.	n.d.
**antibody fragment**	1892	n.d.	n.d.	n.d.	n.d.	n.d.	n.d.	n.d.	n.d.	n.d.	n.d.	n.d.	n.d.	262
**antibody fragment**	1998	n.d.	n.d.	n.d.	n.d.	n.d.	n.d.	n.d.	n.d.	42	112	149	262	189
**antibody fragment**	2006	n.d.	n.d.	n.d.	n.d.	n.d.	n.d.	n.d.	n.d.	n.d.	n.d.	n.d.	n.d.	n.d.
**antibody fragment**	2152	n.d.	n.d.	n.d.	n.d.	n.d.	n.d.	n.d.	n.d.	20	n.d.	n.d.	n.d.	n.d.
**antibody fragment**	2331	n.d.	n.d.	n.d.	n.d.	n.d.	n.d.	n.d.	n.d.	n.d.	45	n.d.	88	n.d.
**antibody fragment**	2508	n.d.	n.d.	n.d.	n.d.	n.d.	n.d.	n.d.	n.d.	n.d.	20	70	66	94
**antibody fragment**	2819	n.d.	n.d.	n.d.	n.d.	n.d.	n.d.	n.d.	n.d.	n.d.	20	33	56	56
**antibody fragment**	3336	n.d.	n.d.	n.d.	n.d.	n.d.	n.d.	n.d.	n.d.	n.d.	n.d.	20	20	20
**antibody fragment**	3461	n.d.	n.d.	n.d.	n.d.	n.d.	n.d.	n.d.	n.d.	n.d.	n.d.	20	20	20

a) Imputed values necessary for optimized Gauss-fit are shown in red and are equal to the background intensity at the given m/z.
b) n.d.: value not determined / ion signal not present.

Table S5. continued

peptide 3, measurement 2

ion / charge state	m/z	ΔCV												
		2	4	6	8	12	16	20	30	40	50	60	70	80
**peptide dimer / 1+**	3590.78	5	5	5	5	10	10	10	10	10	10	10	10	10
**peptide 4 / 1+**	1795.89	5	8	7	7	11	8	9	19	38	62	74	89	137
**peptide 4 / 2+**	898.47	45	75	135	185	377	732	1147	2019	3338	4612	5142	5472	5429
**peptide 4 / 3+**	599.34	6	14	15	25	43	87	118	198	250	342	356	349	316
**peptide 4 / 4+**	449.72	5	5	5	5	10	10	10	10	10	10	10	10	10
**antibody+0 pep / 29+**	5055	50	50	50	50	50	50	50	50	50	50	50	50	50
**antibody+0 pep / 28+**	5234	76	171	174	192	215	218	223	236	264	268	283	271	266
**antibody+0 pep / 27+**	5430	545	1165	1290	1246	1422	1530	1572	1649	1801	1933	1961	1943	1883
**antibody+0 pep / 26+**	5638	1618	3480	3637	3875	4171	4428	4714	4963	5319	5840	6036	5971	5947
**antibody+0 pep / 25+**	5864	2175	4328	4653	4758	5145	5645	5757	6377	6945	7690	8225	8215	8136
**antibody+0 pep / 24+**	6108	1330	2696	2940	3096	3359	3468	3719	4034	4666	5335	5481	5702	5879
**antibody+0 pep / 23+**	6378	468	933	1013	1096	1147	1243	1313	1547	1849	2104	2315	2414	2514
**antibody+0 pep / 22+**	6664	50	50	50	50	50	50	50	50	50	50	50	50	50
**antibody+1 pep/ 29+**	5119	50	50	50	50	50	50	50	50	50	50	50	50	50
**antibody+1 pep/ 28+**	5300	97	219	245	254	274	313	298	302	303	341	334	322	323
**antibody+1 pep/ 27+**	5497	621	1309	1401	1409	1611	1698	1798	1783	2003	1974	2016	1920	1902
**antibody+1 pep/ 26+**	5708	1581	3249	3564	3604	3944	4115	4260	4404	4846	5019	5006	5090	4979
**antibody+1 pep/ 25+**	5937	1683	3432	3747	3790	4052	4261	4474	4622	5016	5463	5508	5621	5576
**antibody+1 pep/ 24+**	6185	916	1838	2047	2080	2253	2294	2368	2564	2881	3073	3268	3303	3386
**antibody+1 pep/ 23+**	6459	264	505	563	635	651	680	724	835	915	1042	1110	1227	1170
**antibody+1 pep/ 22+**	6747	50	50	50	50	50	50	50	50	50	50	50	50	50
**antibody+2 peps/ 29+**	5178	50	50	50	50	50	50	50	50	50	50	50	50	50
**antibody+2 peps/ 28+**	5364	41	85	81	94	116	101	111	110	113	117	114	129	102
**antibody+2 peps/ 27+**	5555	193	398	451	420	473	469	516	514	543	527	550	507	539
**antibody+2 peps/ 26+**	5778	424	850	885	866	952	1040	1013	1056	1098	1145	1096	1073	1087
**antibody+2 peps/ 25+**	6009	417	737	802	833	877	929	944	956	992	1010	1004	1008	1006
**antibody+2 peps/ 24+**	6256	181	343	368	402	403	444	451	515	520	547	546	546	544
**antibody+2 peps/ 23+**	6530	42	94	106	113	108	123	132	139	153	183	193	195	189

**antibody+2 peps/ 22+**	6825	50	50	50	50	50	50	50	50	50	50	50	50	50
**antibody fragment**	1000	n.d.	n.d.	n.d.	n.d.	n.d.	n.d.	n.d.	n.d.	n.d.	n.d.	n.d.	n.d.	20
**antibody fragment**	1200	n.d.	n.d.	n.d.	n.d.	n.d.	n.d.	n.d.	n.d.	n.d.	n.d.	20	20	20
**antibody fragment**	1324	n.d.	n.d.	n.d.	n.d.	n.d.	n.d.	n.d.	n.d.	20	20	20	20	37
**antibody fragment**	1471	n.d.	n.d.	n.d.	n.d.	n.d.	n.d.	n.d.	n.d.	20	20	50	101	336
**antibody fragment**	1554	n.d.	n.d.	n.d.	n.d.	n.d.	n.d.	n.d.	n.d.	45	48	n.d.	n.d.	n.d.
**antibody fragment**	1655	n.d.	n.d.	n.d.	n.d.	n.d.	n.d.	n.d.	n.d.	n.d.	n.d.	n.d.	272	679
**antibody fragment**	1751	n.d.	n.d.	n.d.	n.d.	n.d.	n.d.	n.d.	n.d.	90	128	136	n.d.	n.d.
**antibody fragment**	1892	n.d.	n.d.	n.d.	n.d.	n.d.	n.d.	n.d.	n.d.	n.d.	n.d.	n.d.	n.d.	403
**antibody fragment**	1998	n.d.	n.d.	n.d.	n.d.	n.d.	n.d.	n.d.	n.d.	n.d.	142	195	246	281
**antibody fragment**	2006	n.d.	n.d.	n.d.	n.d.	n.d.	n.d.	n.d.	n.d.	58	n.d.	n.d.	n.d.	n.d.
**antibody fragment**	2152	n.d.	n.d.	n.d.	n.d.	n.d.	n.d.	n.d.	n.d.	20	n.d.	n.d.	n.d.	n.d.
**antibody fragment**	2189	n.d.	n.d.	n.d.	n.d.	n.d.	n.d.	n.d.	n.d.	20	n.d.	n.d.	n.d.	165
**antibody fragment**	2331	n.d.	n.d.	n.d.	n.d.	n.d.	n.d.	n.d.	n.d.	n.d.	53	n.d.	n.d.	153
**antibody fragment**	2508	n.d.	n.d.	n.d.	n.d.	n.d.	n.d.	n.d.	n.d.	n.d.	29	91	123	120
**antibody fragment**	2819	n.d.	n.d.	n.d.	n.d.	n.d.	n.d.	n.d.	n.d.	n.d.	20	28	63	69
**antibody fragment**	3336	n.d.	n.d.	n.d.	n.d.	n.d.	n.d.	n.d.	n.d.	n.d.	20	20	20	20
**antibody fragment**	3461	n.d.	n.d.	n.d.	n.d.	n.d.	n.d.	n.d.	n.d.	n.d.	n.d.	20	20	20

a) Imputed values necessary for optimized Gauss-fit are shown in red and are equal to the background intensity at the given m/z.
b) n.d.: value not determined / ion signal not present.

Table S6. Ion species, charge states, m/z values and intensities for anti-Troponin I antibody complexed with Troponin I peptide 4 at measured collision cell voltage differences. [a,b)]

peptide 4, measurement 1

ion / charge state	m/z	ΔCV										
		4	8	12	16	20	30	40	50	60	70	80
**peptide dimer / 1+**	3541.90	2	2	5	5	5	10	10	10	10	10	10
**peptide 4 / 1+**	1771.89	2	4	7	8	8	15	23	42	69	123	139
**peptide 4 / 2+**	886.47	14	73	274	512	771	1049	1403	2237	3390	4339	4191
**peptide 4 / 3+**	591.65	2	4	6	6	5	7	7	8	11	15	14
**peptide 4 / 4+**	443.73	2	2	5	5	5	10	10	10	10	10	10
**antibody+0 pep / 28+**	5233	20	20	20	20	20	20	20	20	20	20	20
**antibody+0 pep / 27+**	5424	39	115	180	164	153	105,5	88	108	124	173	126
**antibody+0 pep / 26+**	5631	229	679	1051	1146	997	750	681	769	908	1137	960
**antibody+0 pep / 25+**	5861	575	1879	2695	2909	2691	2108	1949	2224	2661	3236	2878
**antibody+0 pep / 24+**	6102	688	2318	3307	3649	3561	2684	2686	2940	3682	4286	3892
**antibody+0 pep / 23+**	6372	414	1524	2068	2365	2363	1802	1974	2081	2550	3063	2914
**antibody+0 pep / 22+**	6660	140	536	746	851	876	704	751	856	1042	1200	1246
**antibody+0 pep / 21+**	6984	34	124	155	197	193	155	193	207	271	305	354
**antibody+0 pep / 20+**	7326	20	20	20	20	20	20	20	20	20	20	20
**antibody+1 pep/ 28+**	5296	20	20	20	20	20	20	20	20	20	20	20
**antibody+1 pep/ 27+**	5491	35	102	165	171	152	110	84	112	112	138	109
**antibody+1 pep/ 26+**	5700	187	631	957	983	890	707	535	646	679	830	711
**antibody+1 pep/ 25+**	5931	422	1508	2171	2290	2144	1712	1500	1579	1793	2144	1886
**antibody+1 pep/ 24+**	6178	478	1781	2581	2781	2647	2137	1898	2086	2360	2618	2577
**antibody+1 pep/ 23+**	6448	261	1027	1429	1632	1640	1253	1231	1314	1462	1755	1677
**antibody+1 pep/ 22+**	6744	93	333	474	544	560	450	459	484	587	663	694
**antibody+1 pep/ 21+**	7064	17	67	93	119	122	101	113	116	144	167	188
**antibody+1 pep/ 20+**	7414	20	20	20	20	20	20	20	20	20	20	20
**antibody+2 peps/ 28+**	5358	10	20	20	20	20	20	20	20	20	20	20
**antibody+2 peps/ 27+**	5557	13	41	66	66	56	47	42	44	42	54	44
**antibody+2 peps/ 26+**	5772	44	170	226	230	213	188	122	145	151	164	145
**antibody+2 peps/ 25+**	6000	93	353	489	539	496	424	327	338	344	386	356
**antibody+2 peps/ 24+**	6248	102	398	574	620	630	503	406	413	432	492	447
**antibody+2 peps/ 23+**	6522	54	207	288	338	340	266	248	271	260	278	270
**antibody+2 peps/ 22+**	6823	18	68	90	111	113	93	87	88	115	116	116

**antibody+2 peps/ 21+**	7142	7	16	24	25	25	25	26	27	41	43	54
**antibody+2 peps/ 20+**	7501	10	20	20	20	20	20	20	20	20	20	20
**antibody fragment**	1000	n.d.	n.d.	n.d.	n.d.	n.d.	n.d.	n.d.	n.d.	n.d.	n.d.	10
**antibody fragment**	1204	n.d.	n.d.	n.d.	n.d.	n.d.	n.d.	n.d.	n.d.	10	10	10
**antibody fragment**	1324	n.d.	n.d.	n.d.	n.d.	n.d.	n.d.	n.d.	n.d.	10	10	17
**antibody fragment**	1471	n.d.	n.d.	n.d.	n.d.	n.d.	n.d.	n.d.	n.d.	n.d.	73	186
**antibody fragment**	1554	n.d.	n.d.	n.d.	n.d.	n.d.	n.d.	n.d.	n.d.	14	n.d.	n.d.
**antibody fragment**	1655	n.d.	n.d.	n.d.	n.d.	n.d.	n.d.	n.d.	n.d.	n.d.	181	395
**antibody fragment**	1751	n.d.	n.d.	n.d.	n.d.	n.d.	n.d.	n.d.	n.d.	55	n.d.	n.d.
**antibody fragment**	1998	n.d.	n.d.	n.d.	n.d.	n.d.	n.d.	n.d.	n.d.	66	192	233
**antibody fragment**	2189	n.d.	n.d.	n.d.	n.d.	n.d.	n.d.	n.d.	n.d.	n.d.	64	n.d.
**antibody fragment**	2331	n.d.	n.d.	n.d.	n.d.	n.d.	n.d.	n.d.	n.d.	13	56	113
**antibody fragment**	2508	n.d.	n.d.	n.d.	n.d.	n.d.	n.d.	n.d.	n.d.	n.d.	n.d.	24
**antibody fragment**	2819	n.d.	n.d.	n.d.	n.d.	n.d.	n.d.	n.d.	n.d.	10	10	10
**antibody fragment**	3336	n.d.	n.d.	n.d.	n.d.	n.d.	n.d.	n.d.	n.d.	10	10	10

a) Imputed values necessary for optimized Gauss-fit are shown in red and are equal to the background intensity at the given m/z.
b) n.d.: value not determined / ion signal not present.

Table S6. continued

peptide 4, measurement 2

ion / charge state	m/z	ΔCV												
		**2**	**4**	**6**	**8**	**12**	**16**	**20**	**30**	**40**	**50**	**60**	**70**	**80**
**peptide dimer / 1+**	3541.90	5	5	5	10	10	10	10	10	10	10	10	10	10
**peptide 4 / 1+**	1771.89	5	8	6	13	11	21	25	81	150	171	229	380	373
**peptide 4 / 2+**	886.47	47	119	344	461	869	1224	1673	3246	5051	4432	4853	6434	5407
**peptide 4 / 3+**	591.65	3	4	5	7	5	6	9	9	12	7	12	12	7
**peptide 4 / 4+**	443.73	5	5	5	5	10	10	10	10	10	10	10	10	10
**antibody+0 pep / 28+**	5226	50	50	50	50	50	50	50	50	50	50	50	50	50
**antibody+0 pep / 27+**	5418	436	676	1395	1467	1478	1349	1240	1647	1898	1122	1247	1810	1310
**antibody+0 pep / 26+**	5628	2170	3177	5486	5741	5461	5097	4941	6298	7239	4685	5168	7059	5432
**antibody+0 pep / 25+**	5851	4016	5655	8707	8696	8594	7984	7973	9810	10794	8099	8887	11322	9130
**antibody+0 pep / 24+**	6095	3360	4622	6669	6735	6614	6347	6138	7767	8497	6909	7655	9273	7894
**antibody+0 pep / 23+**	6364	1492	2087	2815	2837	2909	2727	2770	3633	4026	3598	3897	4640	4214
**antibody+0 pep / 22+**	6654	367	544	708	722	744	737	787	1049	1233	1220	1318	1615	1465
**antibody+0 pep / 21+**	6968	50	50	50	50	50	50	50	50	50	50	50	50	50
**antibody+1 pep/ 28+**	5290	50	50	50	50	50	50	50	50	50	50	50	50	50
**antibody+1 pep/ 27+**	5483	410	578	1145	1142	1077	1046	1069	1278	1425	910	1023	1285	949
**antibody+1 pep/ 26+**	5699	1677	2420	3779	3894	3797	3546	3622	4253	4774	3328	3509	4354	3504
**antibody+1 pep/ 25+**	5921	2684	3793	5324	5418	5163	4860	4947	5936	6230	4860	5134	6313	5213
**antibody+1 pep/ 24+**	6173	2121	2851	3926	3814	3723	3550	3603	4274	4643	3838	4094	4936	4170
**antibody+1 pep/ 23+**	6442	851	1117	1522	1548	1506	1445	1474	1714	1930	1787	1937	2247	2100
**antibody+1 pep/ 22+**	6730	196	282	388	366	359	402	405	468	584	595	638	807	728
**antibody+1 pep/ 21+**	7052	50	50	50	50	50	50	50	50	50	50	50	50	50
**antibody+2 peps/ 28+**	5353	50	50	50	50	50	50	50	50	50	50	50	50	50
**antibody+2 peps/ 27+**	5555	116	166	271	280	258	241	283	301	340	245	265	309	263
**antibody+2 peps/ 26+**	5762	354	489	715	681	676	641	688	748	824	647	677	744	612
**antibody+2 peps/ 25+**	5997	533	703	962	919	892	850	924	968	1058	843	885	995	854
**antibody+2 peps/ 24+**	6246	379	528	714	685	624	613	675	726	795	642	668	728	682
**antibody+2 peps/ 23+**	6515	141	193	267	241	257	255	257	279	310	289	279	350	322
**antibody+2 peps/ 22+**	6808	34	54	66	68	68	68	75	91	118	118	129	158	141
**antibody+2 peps/ 21+**	7137	50	50	50	50	50	50	50	50	50	50	50	50	50

**antibody fragment**	1000	n.d.	n.d.	n.d.	n.d.	n.d.	n.d.	n.d.	20	20	20	20	20	20
**antibody fragment**	1200	n.d.	n.d.	n.d.	n.d.	n.d.	n.d.	n.d.	20	20	20	20	20	20
**antibody fragment**	1324	n.d.	n.d.	n.d.	n.d.	n.d.	n.d.	n.d.	20	20	20	20	20	20
**antibody fragment**	1471	n.d.	n.d.	n.d.	n.d.	n.d.	n.d.	n.d.	n.d.	n.d.	17	41	103	201
**antibody fragment**	1554	n.d.	n.d.	n.d.	n.d.	n.d.	n.d.	n.d.	31	43	n.d.	n.d.	n.d.	n.d.
**antibody fragment**	1655	n.d.	n.d.	n.d.	n.d.	n.d.	n.d.	n.d.	n.d.	n.d.	37	75	290	n.d.
**antibody fragment**	1751	n.d.	n.d.	n.d.	n.d.	n.d.	n.d.	n.d.	49	110	91	95	n.d.	n.d.
**antibody fragment**	1892	n.d.	n.d.	n.d.	n.d.	n.d.	n.d.	n.d.	n.d.	n.d.	n.d.	n.d.	n.d.	441
**antibody fragment**	1998	n.d.	n.d.	n.d.	n.d.	n.d.	n.d.	n.d.	40	n.d.	153	225	n.d.	n.d.
**antibody fragment**	2006	n.d.	n.d.	n.d.	n.d.	n.d.	n.d.	n.d.	n.d.	104	n.d.	n.d.	n.d.	n.d.
**antibody fragment**	2189	n.d.	n.d.	n.d.	n.d.	n.d.	n.d.	n.d.	20	n.d.	n.d.	n.d.	n.d.	n.d.
**antibody fragment**	2331	n.d.	n.d.	n.d.	n.d.	n.d.	n.d.	n.d.	20	62	96	n.d.	n.d.	n.d.
**antibody fragment**	2392	n.d.	n.d.	n.d.	n.d.	n.d.	n.d.	n.d.	20	n.d.	n.d.	n.d.	n.d.	n.d.
**antibody fragment**	2508	n.d.	n.d.	n.d.	n.d.	n.d.	n.d.	n.d.	n.d.	50	n.d.	284	535	484
**antibody fragment**	2592	n.d.	n.d.	n.d.	n.d.	n.d.	n.d.	n.d.	n.d.	20	20	n.d.	n.d.	n.d.
**antibody fragment**	2648	n.d.	n.d.	n.d.	n.d.	n.d.	n.d.	n.d.	n.d.	20	20	n.d.	n.d.	n.d.
**antibody fragment**	2819	n.d.	n.d.	n.d.	n.d.	n.d.	n.d.	n.d.	n.d.	64	88	144	485	n.d.
**antibody fragment**	2985	n.d.	20	20	20	20	20	20	20	n.d.	n.d.	20	20	20
**antibody fragment**	3222	n.d.	20	20	20	20	20	20	20	n.d.	n.d.	20	20	20
**antibody fragment**	3336	n.d.	24	81	166	192	202	250	315	297	246	225	204	168
**antibody fragment**	3590	n.d.	38	237	452	489	464	525	662	679	622	580	646	507
**antibody fragment**	3886	n.d.	35	123	156	175	154	202	206	221	220	213	259	259
**antibody fragment**	4000	n.d.	20	20	20	20	20	20	20	20	20	20	20	20
**antibody fragment**	4200	n.d.	20	20	20	20	20	20	20	20	20	20	20	20

a) Imputed values necessary for optimized Gauss-fit are shown in red and are equal to the background intensity at the given m/z.
b) n.d.: value not determined / ion signal not present.

Table S6. continued

peptide 4, measurement 3

ion / charge state	m/z	ΔCV												
		2	4	6	8	12	16	20	30	40	50	60	70	80
peptide dimer / 1+	3541.90	5	5	5	5	10	10	10	10	10	10	10	10	10
peptide 4 / 1+	1771.89	5	7	9	7	9	19	25	31	138	200	265	353	442
peptide 4 / 2+	886.47	43	104	206	351	686	1244	1716	1926	4558	5464	5629	5844	5629
peptide 4 / 3+	591.65	3	3	3	3	3	5	6	6	7	9	7	9	7
peptide 4 / 4+	443.73	5	5	5	5	10	10	10	10	10	10	10	10	10
antibody+0 pep / 28+	5226	50	50	50	50	50	50	50	50	50	50	50	50	50
antibody+0 pep / 27+	5418	342	568	875	987	1051	1311	1272	1201	1612	1720	1602	1666	1579
antibody+0 pep / 26+	5628	1783	2597	3344	3712	4007	4827	4633	4590	5719	6236	6056	6185	6034
antibody+0 pep / 25+	5851	3190	4278	5493	5749	6319	7271	7397	7160	8888	9536	9443	9634	9325
antibody+0 pep / 24+	6095	2649	3497	4320	4429	4933	5552	5877	5662	7166	8005	7891	7885	7678
antibody+0 pep / 23+	6364	1262	1633	1898	2005	2308	2510	2724	2574	3632	4025	4099	4062	4172
antibody+0 pep / 22+	6654	327	455	538	550	610	677	815	773	1177	1300	1472	1418	1401
antibody+0 pep / 21+	6968	50	50	50	50	50	50	50	50	50	50	50	50	50
antibody+1 pep/ 28+	5290	50	50	50	50	50	50	50	50	50	50	50	50	50
antibody+1 pep/ 27+	5483	332	510	695	802	917	1056	979	983	1205	1287	1164	1189	1111
antibody+1 pep/ 26+	5699	1436	2035	2532	2873	3068	3551	3432	3337	3883	4156	3948	3939	3765
antibody+1 pep/ 25+	5921	2226	2971	3704	3887	4230	4816	4794	4632	5500	5744	5582	5638	5444
antibody+1 pep/ 24+	6173	1862	2330	2751	2890	3118	3415	3517	3405	4133	4351	4449	4476	4270
antibody+1 pep/ 23+	6442	743	900	1143	1223	1291	1379	1514	1436	1897	1998	2123	2033	1991
antibody+1 pep/ 22+	6730	183	239	309	306	338	366	427	380	592	679	725	672	731
antibody+1 pep/ 21+	7052	50	50	50	50	50	50	50	50	50	50	50	50	50
antibody+2 peps/ 28+	5353	50	50	50	50	50	50	50	50	50	50	50	50	50
antibody+2 peps/ 27+	5555	97	133	198	216	212	257	245	262	269	303	292	300	269
antibody+2 peps/ 26+	5762	314	430	510	591	603	660	671	632	708	744	677	712	664
antibody+2 peps/ 25+	5997	448	617	718	767	847	898	909	835	956	1010	949	926	913
antibody+2 peps/ 24+	6246	347	430	510	566	561	626	671	615	717	712	700	669	687
antibody+2 peps/ 23+	6515	132	165	201	207	228	249	278	249	299	325	338	306	307
antibody+2 peps/ 22+	6808	34	41	49	54	61	68	76	76	115	136	129	132	134
antibody+2 peps/ 21+	7137	50	50	50	50	50	50	50	50	50	50	50	50	50

**antibody fragment**	1000	n.d.	n.d.	n.d.	n.d.	n.d.	n.d.	n.d.	n.d.	20	20	20	20	20
**antibody fragment**	1200	n.d.	n.d.	n.d.	n.d.	n.d.	n.d.	n.d.	n.d.	20	20	20	20	20
**antibody fragment**	1324	n.d.	n.d.	n.d.	n.d.	n.d.	n.d.	n.d.	n.d.	n.d.	n.d.	n.d.	n.d.	36
**antibody fragment**	1471	n.d.	n.d.	n.d.	n.d.	n.d.	n.d.	n.d.	n.d.	n.d.	n.d.	53	94	275
**antibody fragment**	1554	n.d.	n.d.	n.d.	n.d.	n.d.	n.d.	n.d.	n.d.	27	27	n.d.	n.d.	n.d.
**antibody fragment**	1655	n.d.	n.d.	n.d.	n.d.	n.d.	n.d.	n.d.	n.d.	n.d.	54	128	224	626
**antibody fragment**	1751	n.d.	n.d.	n.d.	n.d.	n.d.	n.d.	n.d.	n.d.	113	90	n.d.	n.d.	n.d.
**antibody fragment**	1892	n.d.	n.d.	n.d.	n.d.	n.d.	n.d.	n.d.	n.d.	n.d.	n.d.	n.d.	n.d.	487
**antibody fragment**	1998	n.d.	n.d.	n.d.	n.d.	n.d.	n.d.	n.d.	n.d.	89	169	225	231	244
**antibody fragment**	2189	n.d.	n.d.	n.d.	n.d.	n.d.	n.d.	n.d.	n.d.	n.d.	n.d.	123	177	n.d.
**antibody fragment**	2331	n.d.	n.d.	n.d.	n.d.	n.d.	n.d.	n.d.	n.d.	37	82	100	183	n.d.
**antibody fragment**	2592	n.d.	n.d.	n.d.	n.d.	n.d.	n.d.	n.d.	n.d.	20	20	20	20	20
**antibody fragment**	2648	n.d.	n.d.	n.d.	n.d.	n.d.	n.d.	n.d.	n.d.	20	20	20	20	20
**antibody fragment**	2819	n.d.	n.d.	n.d.	n.d.	n.d.	n.d.	n.d.	n.d.	46	75	n.d.	n.d.	n.d.
**antibody fragment**	2985	n.d.	20	20	20	20	20	20	20	n.d.	n.d.	n.d.	n.d.	n.d.
**antibody fragment**	3222	n.d.	20	20	20	20	20	20	20	n.d.	n.d.	n.d.	n.d.	n.d.
**antibody fragment**	3336	n.d.	18	78	132	137	223	279	283	294	267	232	190	189
**antibody fragment**	3590	n.d.	44	227	401	419	462	564	564	671	704	697	607	517
**antibody fragment**	3886	n.d.	33	108	137	140	162	172	181	216	240	255	249	281
**antibody fragment**	4000	n.d.	20	20	20	20	20	20	20	20	20	20	20	20
**antibody fragment**	4200	n.d.	20	20	20	20	20	20	20	20	20	20	20	20

a) Imputed values necessary for optimized Gauss-fit are shown in red and are equal to the background intensity at the given m/z.
b) n.d.: value not determined / ion signal not present.

Table S6. continued

peptide 4, measurement 4

ion / charge state	m/z	ΔCV												
		**2**	**4**	**6**	**8**	**12**	**16**	**20**	**30**	**40**	**50**	**60**	**70**	**80**
**peptide dimer / 1+**	3541.90	2	3	5	5	5	5	5	10	10	10	10	10	10
**peptide 4 / 1+**	1771.89	3	3	6	7	8	10	12	35	73	143	209	230	281
**peptide 4 / 2+**	886.47	11	28	57	78	247	496	738	2309	4426	6054	6553	6740	7153
**peptide 4 / 3+**	591.65	2	4	4	5	5	7	8	15	20	26	32	28	34
**peptide 4 / 4+**	443.73	2	3	5	5	5	5	5	10	10	10	10	10	10
**antibody+0 pep / 29+**	5050	50	50	50	50	50	50	50	50	50	50	50	50	50
**antibody+0 pep / 28+**	5230	82	114	208	253	291	344	376	611	940	1125	1161	1172	1385
**antibody+0 pep / 27+**	5424	507	726	1257	1549	1770	2246	2318	3562	5382	6152	6397	6508	7402
**antibody+0 pep / 26+**	5631	1257	1924	2930	3670	4408	5637	6085	8857	12636	14361	14940	15162	16834
**antibody+0 pep / 25+**	5857	1446	2222	3188	4167	4971	6444	6969	9993	13686	15557	16407	16550	17684
**antibody+0 pep / 24+**	6103	802	1248	1811	2385	2963	3860	4216	5722	7815	8839	9404	9419	9836
**antibody+0 pep / 23+**	6368	261	403	606	791	997	1342	1487	1984	2667	3037	3290	3377	3498
**antibody+0 pep / 22+**	6656	51	67	98	132	168	235	246	371	491	600	706	756	802
**antibody+0 pep / 21+**	6973	50	50	50	50	50	50	50	50	50	50	50	50	50
**antibody+1 pep/ 29+**	5110	20	50	50	50	50	50	50	50	50	50	50	50	50
**antibody+1 pep/ 28+**	5294	75	103	160	172	214	248	257	340	498	547	544	546	595
**antibody+1 pep/ 27+**	5486	300	451	705	847	974	1186	1233	1747	2375	2654	2602	2555	2759
**antibody+1 pep/ 26+**	5701	664	998	1476	1817	2083	2575	2790	3749	4980	5406	5521	5459	5743
**antibody+1 pep/ 25+**	5928	641	1029	1414	1747	2072	2545	2758	3670	4612	4995	5111	5075	5293
**antibody+1 pep/ 24+**	6175	326	520	726	994	1091	1389	1510	1865	2466	2545	2593	2546	2701
**antibody+1 pep/ 23+**	6443	101	152	203	277	309	395	470	573	752	799	860	925	932
**antibody+1 pep/ 22+**	6737	26	27	34	48	51	67	85	117	179	204	268	292	314
**antibody+1 pep/ 21+**	7057	20	50	50	50	50	50	50	50	50	50	50	50	50
**antibody+2 peps/ 29+**	5170	10	20	20	20	20	20	20	50	50	50	50	50	50
**antibody+2 peps/ 28+**	5355	27	43	54	61	59	64	71	79	126	133	122	123	124
**antibody+2 peps/ 27+**	5555	78	110	154	182	203	226	226	286	383	407	388	399	395
**antibody+2 peps/ 26+**	5767	129	222	288	344	369	457	476	558	694	705	655	626	678
**antibody+2 peps/ 25+**	5997	127	191	290	325	353	417	431	517	616	620	600	591	577
**antibody+2 peps/ 24+**	6245	71	135	150	193	208	246	247	308	332	370	356	316	326
**antibody+2 peps/ 23+**	6519	20	35	55	57	73	77	90	124	162	162	167	168	164
**antibody+2 peps/ 22+**	6815	8	16	14	14	21	27	33	49	59	84	90	106	101

**antibody+2 peps/ 21+**	7139	10	20	20	20	20	20	20	50	50	50	50	50	50
**antibody fragment**	1000	n.d.	n.d.	n.d.	n.d.	n.d.	n.d.	n.d.	n.d.	n.d.	n.d.	n.d.	n.d.	20
**antibody fragment**	1200	n.d.	n.d.	n.d.	n.d.	n.d.	n.d.	n.d.	n.d.	n.d.	n.d.	20	20	20
**antibody fragment**	1324	n.d.	n.d.	n.d.	n.d.	n.d.	n.d.	n.d.	n.d.	20	20	20	20	97
**antibody fragment**	1471	n.d.	n.d.	n.d.	n.d.	n.d.	n.d.	n.d.	n.d.	20	20	101	252	804
**antibody fragment**	1554	n.d.	n.d.	n.d.	n.d.	n.d.	n.d.	n.d.	n.d.	59	95	n.d.	n.d.	n.d.
**antibody fragment**	1655	n.d.	n.d.	n.d.	n.d.	n.d.	n.d.	n.d.	n.d.	n.d.	n.d.	n.d.	439	1449
**antibody fragment**	1751	n.d.	n.d.	n.d.	n.d.	n.d.	n.d.	n.d.	n.d.	108	169	220	n.d.	n.d.
**antibody fragment**	1998	n.d.	n.d.	n.d.	n.d.	n.d.	n.d.	n.d.	n.d.	133	308	500	771	780
**antibody fragment**	2189	n.d.	n.d.	n.d.	n.d.	n.d.	n.d.	n.d.	n.d.	64	177	369	486	643
**antibody fragment**	2331	n.d.	n.d.	n.d.	n.d.	n.d.	n.d.	n.d.	n.d.	n.d.	n.d.	131	n.d.	n.d.
**antibody fragment**	2392	n.d.	n.d.	n.d.	n.d.	n.d.	n.d.	n.d.	n.d.	30	82	n.d.	218	n.d.
**antibody fragment**	2508	n.d.	n.d.	n.d.	n.d.	n.d.	n.d.	n.d.	n.d.	20	55	64	121	149
**antibody fragment**	2592	n.d.	n.d.	n.d.	n.d.	n.d.	n.d.	n.d.	n.d.	20	20	20	n.d.	n.d.
**antibody fragment**	2648	n.d.	n.d.	n.d.	n.d.	n.d.	n.d.	n.d.	n.d.	n.d.	20	20	n.d.	n.d.
**antibody fragment**	2819	n.d.	n.d.	n.d.	n.d.	n.d.	n.d.	n.d.	n.d.	n.d.	n.d.	n.d.	76	87
**antibody fragment**	2985	n.d.	n.d.	n.d.	n.d.	n.d.	n.d.	n.d.	n.d.	n.d.	n.d.	n.d.	20	20
**antibody fragment**	3222	n.d.	n.d.	n.d.	n.d.	n.d.	n.d.	n.d.	n.d.	n.d.	n.d.	n.d.	20	20

a) Imputed values necessary for optimized Gauss-fit are shown in red and are equal to the background intensity at the given m/z.
b) n.d.: value not determined / ion signal not present.

Table S6. continued

peptide 4, measurement 5

ion / charge state	m/z	ΔCV												
		**2**	**4**	**6**	**8**	**12**	**16**	**20**	**30**	**40**	**50**	**60**	**70**	**80**
**peptide dimer / 1+**	3541.90	5	5	5	5	10	10	10	10	10	10	10	10	10
**peptide 4 / 1+**	1771.89	8	10	14	18	19	21	28	75	134	232	350	395	471
**peptide 4 / 2+**	886.47	81	247	558	799	1701	2985	4266	7815	10853	14127	14777	14247	14950
**peptide 4 / 3+**	591.65	4	5	6	10	14	12	24	29	42	68	61	53	53
**peptide 4 / 4+**	443.73	5	5	5	5	10	10	10	10	10	10	10	10	10
**antibody+0 pep / 29+**	5050	50	50	50	50	50	50	50	50	50	50	50	50	50
**antibody+0 pep / 28+**	5230	417	813	1168	1445	2162	2934	3420	3998	4555	5050	4948	4751	4629
**antibody+0 pep / 27+**	5424	2400	4332	6040	6994	9394	11765	12993	15148	17003	18373	18245	17878	17806
**antibody+0 pep / 26+**	5631	5354	9386	12572	14143	17404	20462	22001	24892	27777	30107	30001	29598	29543
**antibody+0 pep / 25+**	5857	5318	8686	11170	12652	14169	16255	16969	18990	20993	22614	23174	22611	22651
**antibody+0 pep / 24+**	6103	2633	4035	5089	5725	5981	6810	6976	7805	8733	9285	9526	9675	9689
**antibody+0 pep / 23+**	6368	782	1116	1347	1574	1539	1723	1817	2116	2439	2764	2801	2891	2935
**antibody+0 pep / 22+**	6656	104	145	169	201	205	234	269	359	483	567	668	707	724
**antibody+0 pep / 21+**	6973	50	50	50	50	50	50	50	50	50	50	50	50	50
**antibody+1 pep/ 29+**	5110	20	50	50	50	50	50	50	50	50	50	50	50	50
**antibody+1 pep/ 28+**	5294	234	448	637	737	1083	1277	1479	1684	1697	1768	1729	1647	1582
**antibody+1 pep/ 27+**	5486	1114	2072	2766	3157	3968	4733	5058	5511	5738	5961	5701	5474	5321
**antibody+1 pep/ 26+**	5701	2349	3887	5042	5670	6587	7469	7834	8183	8714	9131	8761	8180	8256
**antibody+1 pep/ 25+**	5928	1926	3118	3996	4455	4878	5298	5422	5778	5972	6232	6152	5946	5929
**antibody+1 pep/ 24+**	6175	883	1330	1672	1833	1904	2060	2074	2208	2328	2524	2490	2493	2503
**antibody+1 pep/ 23+**	6443	236	324	410	404	471	480	498	570	641	763	810	810	885
**antibody+1 pep/ 22+**	6737	36	53	55	55	65	76	90	141	195	266	329	346	416
**antibody+1 pep/ 21+**	7057	20	50	50	50	50	50	50	50	50	50	50	50	50
**antibody+2 peps/ 29+**	5170	10	10	20	20	20	20	20	50	50	50	50	50	50
**antibody+2 peps/ 28+**	5355	50	110	129	172	245	323	312	323	351	341	332	279	315
**antibody+2 peps/ 27+**	5555	202	342	438	469	622	723	737	783	755	799	760	675	692
**antibody+2 peps/ 26+**	5767	321	568	713	779	915	983	1012	1096	1021	1111	1052	945	952
**antibody+2 peps/ 25+**	5997	284	410	504	590	625	694	746	781	770	802	780	665	700
**antibody+2 peps/ 24+**	6245	124	186	214	287	255	321	355	383	389	452	404	344	377
**antibody+2 peps/ 23+**	6519	44	61	62	67	77	99	107	149	154	215	207	190	208
**antibody+2 peps/ 22+**	6815	11	16	23	24	27	35	40	67	85	105	161	125	124

**antibody+2 peps/ 21+**	7139	10	10	20	20	20	20	20	50	50	50	50	50	50
**antibody fragment**	1000	n.d.	n.d.	n.d.	n.d.	n.d.	n.d.	n.d.	n.d.	n.d.	n.d.	20	20	20
**antibody fragment**	1200	n.d.	n.d.	n.d.	n.d.	n.d.	n.d.	n.d.	n.d.	n.d.	n.d.	20	20	20
**antibody fragment**	1324	n.d.	n.d.	n.d.	n.d.	n.d.	n.d.	n.d.	20	20	20	42	104	224
**antibody fragment**	1471	n.d.	n.d.	n.d.	n.d.	n.d.	n.d.	n.d.	20	20	20	210	623	1656
**antibody fragment**	1554	n.d.	n.d.	n.d.	n.d.	n.d.	n.d.	n.d.	68	101	n.d.	n.d.	n.d.	n.d.
**antibody fragment**	1655	n.d.	n.d.	n.d.	n.d.	n.d.	n.d.	n.d.	n.d.	n.d.	154	396	1208	2774
**antibody fragment**	1751	n.d.	n.d.	n.d.	n.d.	n.d.	n.d.	n.d.	126	323	413	n.d.	n.d.	n.d.
**antibody fragment**	1851	n.d.	n.d.	n.d.	n.d.	n.d.	n.d.	n.d.	n.d.	n.d.	n.d.	n.d.	n.d.	1891
**antibody fragment**	1998	n.d.	n.d.	n.d.	n.d.	n.d.	n.d.	n.d.	84	270	485	658	778	786
**antibody fragment**	2006	n.d.	n.d.	n.d.	n.d.	n.d.	n.d.	n.d.	20	n.d.	n.d.	n.d.	n.d.	n.d.
**antibody fragment**	2069	n.d.	n.d.	n.d.	n.d.	n.d.	n.d.	n.d.	20	n.d.	n.d.	n.d.	n.d.	n.d.
**antibody fragment**	2331	n.d.	n.d.	n.d.	n.d.	n.d.	n.d.	n.d.	n.d.	92	199	324	375	480
**antibody fragment**	2392	n.d.	n.d.	n.d.	n.d.	n.d.	n.d.	n.d.	n.d.	n.d.	n.d.	n.d.	n.d.	n.d.
**antibody fragment**	2508	n.d.	n.d.	n.d.	n.d.	n.d.	n.d.	n.d.	n.d.	49	89	161	203	249
**antibody fragment**	2592	n.d.	n.d.	n.d.	n.d.	n.d.	n.d.	n.d.	n.d.	20	20	n.d.	n.d.	n.d.
**antibody fragment**	2648	n.d.	n.d.	n.d.	n.d.	n.d.	n.d.	n.d.	n.d.	20	20	n.d.	n.d.	n.d.
**antibody fragment**	2819	n.d.	n.d.	n.d.	n.d.	n.d.	n.d.	n.d.	n.d.	n.d.	n.d.	70	128	n.d.
**antibody fragment**	2985	n.d.	n.d.	n.d.	n.d.	n.d.	n.d.	n.d.	n.d.	n.d.	n.d.	20	20	20
**antibody fragment**	3222	n.d.	n.d.	n.d.	n.d.	n.d.	n.d.	n.d.	n.d.	n.d.	n.d.	20	20	20

a) Imputed values necessary for optimized Gauss-fit are shown in red and are equal to the background intensity at the given m/z.
b) n.d.: value not determined / ion signal not present.

Table S7. Ion species, charge states, m/z values and intensities for anti-Troponin I antibody complexed with Troponin I peptide 5 at measured collision cell voltage differences. [a,b)]

peptide 5, measurement 1

ion / charge state	m/z	ΔCV												
		2	4	6	8	12	16	20	30	40	50	60	70	80
**peptide dimer / 1+**	3522.74	10	10	10	10	10	10	10	10	10	10	10	10	10
**peptide 5 / 1+**	1761.87	11	10	18	11	12	18	40	73	136	211	338	475	557
**peptide 5 / 2+**	881.45	180	223	575	586	958	1509	2090	4087	7348	11742	12777	13021	12868
**peptide 5 +Na / 3+**	595.39	13	10	15	37	39	50	68	148	239	377	312	322	271
**peptide 5 / 4+**	441.22	10	10	10	10	10	10	10	10	10	10	10	10	10
**antibody+0 pep / 29+**	5052	50	50	50	50	50	50	50	50	50	50	50	50	50
**antibody+0 pep / 28+**	5233	770	919	774	1008	855	795	804	967	975	1065	963	912	823
**antibody+0 pep / 27+**	5424	3299	3986	3179	4085	3848	3720	3456	4254	4242	4630	4471	4330	4039
**antibody+0 pep / 26+**	5634	6506	7832	6460	8330	8075	8057	7310	8575	8963	9811	9470	9472	9305
**antibody+0 pep / 25+**	5860	5871	7004	6031	7671	7886	7734	7121	8241	8770	9646	9718	9859	9898
**antibody+0 pep / 24+**	6103	2849	3464	3077	3934	4240	4334	3983	4528	5155	5785	6007	6199	6211
**antibody+0 pep / 23+**	6371	797	942	944	1183	1332	1368	1262	1460	1889	2238	2557	2703	2712
**antibody+0 pep / 22+**	6659	50	50	50	50	50	50	50	50	50	50	50	50	50
**antibody+1 pep/ 29+**	5112	50	50	50	50	50	50	50	50	50	50	50	50	50
**antibody+1 pep/ 28+**	5296	968	1133	1001	1197	1012	986	1025	1090	1088	1131	1069	968	875
**antibody+1 pep/ 27+**	5491	4180	4834	4187	4923	4435	4229	4213	4681	4779	4943	4715	4539	4111
**antibody+1 pep/ 26+**	5701	7757	8785	7731	9131	8945	8649	8367	9055	9009	9560	9069	9119	8505
**antibody+1 pep/ 25+**	5930	6205	7065	6673	7702	7734	7771	7235	7859	8131	8481	8383	8412	8281
**antibody+1 pep/ 24+**	6177	2810	3196	3142	3752	3922	3966	3689	3899	4294	4581	4706	4779	4867
**antibody+1 pep/ 23+**	6445	684	847	831	945	1055	1089	1062	1087	1334	1526	1690	1748	1810
**antibody+1 pep/ 22+**	6739	50	50	50	50	50	50	50	50	50	50	50	50	50
**antibody+2 peps/ 29+**	5173	50	50	50	50	50	50	50	50	50	50	50	50	50
**antibody+2 peps/ 28+**	5358	380	403	391	401	359	321	349	373	354	389	335	298	293
**antibody+2 peps/ 27+**	5554	1473	1552	1597	1579	1365	1363	1446	1451	1403	1435	1382	1289	1167
**antibody+2 peps/ 26+**	5768	2643	2774	2915	2819	2639	2558	2676	2692	2611	2610	2436	2381	2214
**antibody+2 peps/ 25+**	6000	1999	2042	2434	2216	2321	2208	2198	2172	2188	2054	2063	1945	1946
**antibody+2 peps/ 24+**	6250	771	824	1008	923	1020	986	949	953	993	995	986	983	990
**antibody+2 peps/ 23+**	6524	185	181	239	215	239	243	234	238	269	311	323	331	351
**antibody+2 peps/ 22+**	6818	50	50	50	50	50	50	50	50	50	50	50	50	50

**antibody fragment**	1000	n.d.	n.d.	n.d.	n.d.	n.d.	n.d.	n.d.	n.d.	n.d.	n.d.	n.d.	n.d.	20
**antibody fragment**	1324	n.d.	n.d.	n.d.	n.d.	n.d.	n.d.	n.d.	n.d.	n.d.	n.d.	n.d.	n.d.	20
**antibody fragment**	1471	n.d.	n.d.	n.d.	n.d.	n.d.	n.d.	n.d.	n.d.	n.d.	n.d.	n.d.	n.d.	287
**antibody fragment**	1554	n.d.	n.d.	n.d.	n.d.	n.d.	n.d.	n.d.	n.d.	n.d.	20	20	20	n.d.
**antibody fragment**	1655	n.d.	n.d.	n.d.	n.d.	n.d.	n.d.	n.d.	n.d.	n.d.	20	20	20	408
**antibody fragment**	1751	n.d.	n.d.	n.d.	n.d.	n.d.	n.d.	n.d.	n.d.	n.d.	20	20	20	n.d.
**antibody fragment**	1851	n.d.	n.d.	n.d.	n.d.	n.d.	n.d.	n.d.	n.d.	n.d.	n.d.	160	475	n.d.
**antibody fragment**	1998	n.d.	n.d.	n.d.	n.d.	n.d.	n.d.	n.d.	n.d.	n.d.	n.d.	243	450	605
**antibody fragment**	2189	n.d.	n.d.	n.d.	n.d.	n.d.	n.d.	n.d.	n.d.	n.d.	57	131	256	299
**antibody fragment**	2508	20	20	20	20	20	20	20	20	20	n.d.	20	20	127
**antibody fragment**	2819	20	20	20	20	20	20	20	20	20	n.d.	20	20	20
**antibody fragment**	3336	167	171	151	182	191	174	205	212	220	175	148	123	118
**antibody fragment**	3590	138	154	163	156	192	202	188	198	194	172	180	149	160
**antibody fragment**	3886	61	77	79	80	90	83	82	95	87	93	80	83	80
**antibody fragment**	4200	20	20	20	20	20	20	20	20	20	20	20	20	20
**antibody fragment**	4400	20	20	20	20	20	20	20	20	20	20	20	20	20

a) Imputed values necessary for optimized Gauss-fit are shown in red and are equal to the background intensity at the given m/z.
b) n.d.: value not determined / ion signal not present.

Table S7. continued

peptide 5, measurement 2

ion / charge state	m/z	ΔCV												
		**2**	**4**	**6**	**8**	**12**	**16**	**20**	**30**	**40**	**50**	**60**	**70**	**80**
**peptide dimer / 1+**	3522.74	10	10	10	10	10	10	10	10	10	10	10	10	10
**peptide 5 / 1+**	1761.87	16	16	16	15	26	29	49	131	229	453	633	889	1034
**peptide 5 / 2+**	881.45	282	459	661	1042	1943	3278	4289	7363	13246	20645	23458	23539	22850
**peptide 5 +Na / 3+**	595.39	25	42	35	56	98	167	129	234	403	629	674	646	603
**peptide 5 / 4+**	441.22	10	10	10	10	10	10	10	10	10	10	10	10	10
**antibody+0 pep / 29+**	5052	50	50	50	50	50	50	50	50	50	50	50	50	50
**antibody+0 pep / 28+**	5233	1101	1292	1341	1129	1182	1230	1047	1077	1001	1112	1048	1005	936
**antibody+0 pep / 27+**	5424	4832	5740	5657	5043	5265	5535	4980	5240	5151	5484	5125	5024	4712
**antibody+0 pep / 26+**	5634	9934	11478	11772	11194	11507	12212	11312	12011	12153	12957	12631	12246	11626
**antibody+0 pep / 25+**	5860	9483	10844	11257	11280	11566	11835	11741	12509	13149	14158	14648	13567	13313
**antibody+0 pep / 24+**	6103	5055	5523	5968	6269	6410	6641	6840	7217	8301	9110	9997	9361	9084
**antibody+0 pep / 23+**	6371	1503	1644	1868	2083	2204	2177	2396	2604	3265	4004	4485	4220	4197
**antibody+0 pep / 22+**	6659	50	50	50	50	50	50	50	50	50	50	50	50	50
**antibody+1 pep/ 29+**	5112	50	50	50	50	50	50	50	50	50	50	50	50	50
**antibody+1 pep/ 28+**	5296	1517	1653	1584	1405	1439	1498	1295	1349	1218	1263	1127	1194	1088
**antibody+1 pep/ 27+**	5491	6629	7102	6922	6375	6674	6825	6191	6480	6115	6374	5813	5746	5341
**antibody+1 pep/ 26+**	5701	12833	13630	13505	13111	13729	13851	13466	13638	13576	13796	13324	12893	12374
**antibody+1 pep/ 25+**	5930	10919	11416	11924	11933	12324	12433	12606	12753	13382	13541	13913	13067	12573
**antibody+1 pep/ 24+**	6177	5164	5403	5746	6352	6463	6592	6719	6902	7663	8079	8542	8085	7949
**antibody+1 pep/ 23+**	6445	1350	1380	1559	1814	1851	1917	2077	2124	2558	2962	3256	3120	3133
**antibody+1 pep/ 22+**	6739	50	50	50	50	50	50	50	50	50	50	50	50	50
**antibody+2 peps/ 29+**	5173	50	50	50	50	50	50	50	50	50	50	50	50	50
**antibody+2 peps/ 28+**	5358	600	635	602	569	528	561	516	532	489	475	411	426	423
**antibody+2 peps/ 27+**	5554	2437	2442	2270	2247	2270	2366	2199	2206	2149	2113	1886	1832	1720
**antibody+2 peps/ 26+**	5768	4496	4449	4192	4308	4437	4542	4449	4401	4236	4094	3919	3726	3632
**antibody+2 peps/ 25+**	6000	3749	3572	3564	3800	3875	3933	3864	3924	3937	3694	3706	3401	3409
**antibody+2 peps/ 24+**	6250	1514	1479	1626	1762	1842	1860	1924	1901	1997	1924	2004	1843	1782
**antibody+2 peps/ 23+**	6524	341	341	379	436	464	453	488	509	588	626	613	591	596
**antibody+2 peps/ 22+**	6818	50	50	50	50	50	50	50	50	50	50	50	50	50

**antibody fragment**	1000	n.d.	n.d.	n.d.	n.d.	n.d.	n.d.	n.d.	20	20	20	20	20	20
**antibody fragment**	1175	n.d.	n.d.	n.d.	n.d.	n.d.	n.d.	n.d.	65	103	175	n.d.	n.d.	n.d.
**antibody fragment**	1324	n.d.	n.d.	n.d.	n.d.	n.d.	n.d.	n.d.	n.d.	n.d.	n.d.	n.d.	n.d.	134
**antibody fragment**	1471	n.d.	n.d.	n.d.	n.d.	n.d.	n.d.	n.d.	n.d.	n.d.	n.d.	n.d.	143	316
**antibody fragment**	1554	n.d.	n.d.	n.d.	n.d.	n.d.	n.d.	n.d.	45	65	89	148	177	n.d.
**antibody fragment**	1655	n.d.	n.d.	n.d.	n.d.	n.d.	n.d.	n.d.	n.d.	n.d.	n.d.	n.d.	n.d.	454
**antibody fragment**	1751	n.d.	n.d.	n.d.	n.d.	n.d.	n.d.	n.d.	n.d.	n.d.	128	197	304	n.d.
**antibody fragment**	1851	n.d.	n.d.	n.d.	n.d.	n.d.	n.d.	n.d.	20	n.d.	n.d.	n.d.	n.d.	n.d.
**antibody fragment**	1892	n.d.	n.d.	n.d.	n.d.	n.d.	n.d.	n.d.	20	n.d.	n.d.	n.d.	n.d.	n.d.
**antibody fragment**	1998	n.d.	n.d.	n.d.	n.d.	n.d.	n.d.	n.d.	n.d.	86	129	194	n.d.	n.d.
**antibody fragment**	2006	n.d.	n.d.	n.d.	n.d.	n.d.	n.d.	n.d.	n.d.	n.d.	n.d.	n.d.	353	436
**antibody fragment**	2160	n.d.	n.d.	n.d.	n.d.	n.d.	n.d.	n.d.	n.d.	20	20	n.d.	n.d.	n.d.
**antibody fragment**	2189	n.d.	n.d.	n.d.	n.d.	n.d.	n.d.	n.d.	n.d.	20	20	n.d.	n.d.	n.d.
**antibody fragment**	2331	n.d.	n.d.	n.d.	n.d.	n.d.	n.d.	n.d.	n.d.	n.d.	n.d.	85	n.d.	157
**antibody fragment**	2508	20	20	20	20	20	20	20	20	20	20	20	138	n.d.
**antibody fragment**	2819	20	20	20	20	20	20	20	20	20	20	20	20	20
**antibody fragment**	3336	167	176	175	189	216	193	219	230	228	192	164	125	116
**antibody fragment**	3590	219	201	225	239	272	268	268	261	259	241	241	226	190
**antibody fragment**	3886	99	97	98	113	112	112	112	112	130	117	113	126	123
**antibody fragment**	4200	20	20	20	20	20	20	20	20	20	20	20	20	20
**antibody fragment**	4400	20	20	20	20	20	20	20	20	20	20	20	20	20

a) Imputed values necessary for optimized Gauss-fit are shown in red and are equal to the background intensity at the given m/z.
b) n.d.: value not determined / ion signal not present.

Table S7. continued

peptide 5, measurement 3

ion / charge state	m/z	ΔCV												
		**2**	**4**	**6**	**8**	**12**	**16**	**20**	**30**	**40**	**50**	**60**	**70**	**80**
**peptide dimer / 1+**	3522.74	10	10	10	10	10	10	10	10	10	10	10	10	10
**peptide 5 / 1+**	1761.87	11	14	13	13	20	29	54	148	219	412	617	924	1021
**peptide 5 / 2+**	881.45	256	314	554	826	1567	2717	4012	6704	12217	19274	22342	22683	22872
**peptide 5 +Na/ 3+**	595.39	15	17	37	38	74	131	167	247	456	688	666	681	597
**peptide 5 / 4+**	441.22	10	10	10	10	10	10	10	10	10	10	10	10	10
**antibody+0 pep / 29+**	5052	50	50	50	50	50	50	50	50	50	50	50	50	50
**antibody+0 pep / 28+**	5233	740	705	897	819	829	1062	1174	1069	1097	997	1082	1092	1108
**antibody+0 pep / 27+**	5424	3344	3399	4178	3921	3978	4802	4981	5070	5080	5082	5369	5339	5442
**antibody+0 pep / 26+**	5634	7454	7858	9070	8872	9076	10150	10723	10931	11298	12005	12579	12628	12848
**antibody+0 pep / 25+**	5860	7549	8235	9033	9119	9329	10021	10534	11128	11856	13331	14188	14161	14426
**antibody+0 pep / 24+**	6103	4359	4716	4997	5161	5389	5517	6011	6490	7434	8663	9453	9557	9730
**antibody+0 pep / 23+**	6371	1364	1498	1570	1684	1771	1742	1896	2242	2902	3728	4082	4351	4298
**antibody+0 pep / 22+**	6659	50	50	50	50	50	50	50	50	50	50	50	50	50
**antibody+1 pep/ 29+**	5112	50	50	50	50	50	50	50	50	50	50	50	50	50
**antibody+1 pep/ 28+**	5296	989	992	1192	1139	1085	1350	1359	1331	1272	1146	1199	1186	1268
**antibody+1 pep/ 27+**	5491	4728	4798	5491	5325	5279	6054	6070	6217	5969	5560	5725	5678	5804
**antibody+1 pep/ 26+**	5701	9821	10257	11217	11330	11253	11999	12550	12623	12760	12840	12953	12826	12824
**antibody+1 pep/ 25+**	5930	9144	9581	10214	10556	10686	10751	11147	11569	11997	12609	12815	12800	12932
**antibody+1 pep/ 24+**	6177	4785	5129	5134	5417	5724	5350	5607	6043	6761	7471	7756	7768	7818
**antibody+1 pep/ 23+**	6445	1278	1383	1398	1514	1624	1493	1528	1749	2210	2692	2839	2962	2989
**antibody+1 pep/ 22+**	6739	50	50	50	50	50	50	50	50	50	50	50	50	50
**antibody+2 peps/ 29+**	5173	50	50	50	50	50	50	50	50	50	50	50	50	50
**antibody+2 peps/ 28+**	5358	433	444	484	503	459	526	507	503	489	416	438	397	418
**antibody+2 peps/ 27+**	5554	1873	1870	2039	2072	1993	2066	2072	2021	1994	1766	1766	1708	1768
**antibody+2 peps/ 26+**	5768	3683	3792	3910	4072	3975	3988	4007	4071	3997	3678	3594	3436	3424
**antibody+2 peps/ 25+**	6000	3138	3348	3350	3534	3611	3392	3457	3526	3564	3418	3293	3214	3252
**antibody+2 peps/ 24+**	6250	1439	1557	1479	1602	1664	1557	1500	1569	1742	1827	1695	1676	1655
**antibody+2 peps/ 23+**	6524	343	384	337	365	390	350	376	387	450	550	521	529	508
**antibody+2 peps/ 22+**	6818	50	50	50	50	50	50	50	50	50	50	50	50	50

**antibody fragment**	1000	n.d.	n.d.	n.d.	n.d.	n.d.	n.d.	n.d.	20	20	20	20	20	20
**antibody fragment**	1175	n.d.	n.d.	n.d.	n.d.	n.d.	n.d.	n.d.	n.d.	n.d.	n.d.	n.d.	n.d.	n.d.
**antibody fragment**	1324	n.d.	n.d.	n.d.	n.d.	n.d.	n.d.	n.d.	n.d.	n.d.	n.d.	n.d.	n.d.	103
**antibody fragment**	1471	n.d.	n.d.	n.d.	n.d.	n.d.	n.d.	n.d.	n.d.	n.d.	n.d.	n.d.	138	342
**antibody fragment**	1554	n.d.	n.d.	n.d.	n.d.	n.d.	n.d.	n.d.	n.d.	n.d.	n.d.	141	n.d.	n.d.
**antibody fragment**	1655	n.d.	n.d.	n.d.	n.d.	n.d.	n.d.	n.d.	n.d.	n.d.	n.d.	n.d.	n.d.	418
**antibody fragment**	1751	n.d.	n.d.	n.d.	n.d.	n.d.	n.d.	n.d.	56	79	123	212	301	n.d.
**antibody fragment**	1851	n.d.	n.d.	n.d.	n.d.	n.d.	n.d.	n.d.	n.d.	n.d.	n.d.	96	141	n.d.
**antibody fragment**	2006	n.d.	n.d.	n.d.	n.d.	n.d.	n.d.	n.d.	33	77	116	n.d.	n.d.	446
**antibody fragment**	2189	n.d.	n.d.	n.d.	n.d.	n.d.	n.d.	n.d.	20	20	20	20	20	n.d.
**antibody fragment**	2207	n.d.	n.d.	n.d.	n.d.	n.d.	n.d.	n.d.	20	20	20	20	20	n.d.
**antibody fragment**	2508	20	20	20	20	20	20	20	20	20	20	20	n.d.	220
**antibody fragment**	2819	20	20	20	20	20	20	20	20	20	20	20	n.d.	n.d.
**antibody fragment**	3336	140	157	155	174	187	163	176	180	192	190	187	151	20
**antibody fragment**	3590	173	176	190	202	201	213	210	216	235	250	248	237	214
**antibody fragment**	3728	n.d.	n.d.	n.d.	n.d.	n.d.	n.d.	n.d.	n.d.	n.d.	n.d.	n.d.	n.d.	214
**antibody fragment**	3886	81	79	91	85	95	89	85	102	110	108	120	115	137
**antibody fragment**	4200	20	20	20	20	20	20	20	20	20	20	20	20	20
**antibody fragment**	4400	20	20	20	20	20	20	20	20	20	20	20	20	20

a) Imputed values necessary for optimized Gauss-fit are shown in red and are equal to the background intensity at the given m/z.
b) n.d.: value not determined / ion signal not present.

Table S7. continued

peptide 5, measurement 4

ion / charge state	m/z	ΔCV 2	4	6	8	12	16	20	30	40	50	60	70	80
**peptide dimer / 1+**	3522.74	5	5	5	5	10	10	10	10	10	10	10	10	10
**peptide 5 / 1+**	1761.87	7	12	9	9	14	18	22	40	67	114	244	316	364
**peptide 5 / 2+**	881.45	95	330	586	953	1748	2953	2604	2853	4909	7857	11720	13183	13547
**peptide 5 / 3+**	588.26	6	8	7	13	17	36	25	27	37	67	69	67	69
**peptide 5 +Na / 3+**	595.39	n.d.	n.d.	n.d.	n.d.	n.d.	n.d.	n.d.	n.d.	n.d.	n.d.	37	26	47
**peptide 5 / 4+**	441.22	5	5	5	5	10	10	10	10	10	10	10	10	10
**antibody+0 pep / 29+**	5054	50	50	50	50	50	50	50	50	50	50	50	50	50
**antibody+0 pep / 28+**	5229	131	213	320	418	544	697	771	752	779	854	881	895	881
**antibody+0 pep / 27+**	5424	859	1334	2135	2534	3219	3858	3870	4147	4138	4636	4810	4880	4791
**antibody+0 pep / 26+**	5634	2125	3426	5179	6065	7548	8709	8853	9619	9754	10742	11006	11315	11204
**antibody+0 pep / 25+**	5860	2292	3875	5604	6622	8248	9187	9201	9816	10488	11490	12216	12578	12537
**antibody+0 pep / 24+**	6103	1282	2103	3076	3755	4478	5040	5044	5443	6201	6809	7279	7446	7293
**antibody+0 pep / 23+**	6371	417	658	946	1155	1416	1539	1664	1800	2141	2492	2699	2768	2824
**antibody+0 pep / 22+**	6655	60	93	135	159	201	242	265	365	427	566	647	679	722
**antibody+0 pep / 21+**	6975	50	50	50	50	50	50	50	50	50	50	50	50	50
**antibody+1 pep/ 29+**	5115	50	50	50	50	50	50	50	50	50	50	50	50	50
**antibody+1 pep/ 28+**	5289	185	290	418	521	652	801	828	841	858	886	892	975	867
**antibody+1 pep/ 27+**	5491	938	1535	2307	2645	3291	3819	3885	3987	3991	4260	4189	4272	4211
**antibody+1 pep/ 26+**	5701	1925	3174	4681	5429	6710	7485	7357	7566	7542	8108	8147	8360	8173
**antibody+1 pep/ 25+**	5930	1736	2828	4150	4869	5817	6498	6309	6657	6661	7126	7333	7169	7173
**antibody+1 pep/ 24+**	6177	872	1330	1969	2328	2782	3039	3017	3049	3331	3554	3722	3670	3658
**antibody+1 pep/ 23+**	6445	232	354	500	606	733	786	805	844	953	1089	1166	1218	1225
**antibody+1 pep/ 22+**	6734	37	50	77	83	95	117	127	163	188	242	314	333	367
**antibody+1 pep/ 21+**	7059	50	50	50	50	50	50	50	50	50	50	50	50	50
**antibody+2 peps/ 29+**	5176	25	50	50	50	50	50	50	50	50	50	50	50	50
**antibody+2 peps/ 28+**	5356	63	114	159	185	241	274	263	269	260	261	278	301	255
**antibody+2 peps/ 27+**	5554	273	439	642	754	912	1064	1015	1029	961	1004	1011	1008	962
**antibody+2 peps/ 26+**	5768	494	773	1157	1334	1558	1699	1692	1680	1637	1602	1610	1612	1607
**antibody+2 peps/ 25+**	6000	377	604	886	1050	1152	1309	1207	1218	1192	1212	1216	1162	1149

**antibody+2 peps/ 24+**	6250	170	230	345	424	488	587	538	525	529	535	565	589	529
**antibody+2 peps/ 23+**	6524	47	61	84	99	124	154	143	159	187	197	199	210	206
**antibody+2 peps/ 22+**	6810	19	17	20	24	27	35	38	44	78	93	116	138	122
**antibody+2 peps/ 21+**	7141	25	50	50	50	50	50	50	50	50	50	50	50	50
**antibody fragment**	1000	n.d.	n.d.	n.d.	n.d.	n.d.	n.d.	n.d.	n.d.	n.d.	n.d.	n.d.	20	20
**antibody fragment**	1175	n.d.	n.d.	n.d.	n.d.	n.d.	n.d.	n.d.	n.d.	n.d.	n.d.	n.d.	20	20
**antibody fragment**	1324	n.d.	n.d.	n.d.	n.d.	n.d.	n.d.	n.d.	n.d.	n.d.	20	20	40	96
**antibody fragment**	1440	n.d.	n.d.	n.d.	n.d.	n.d.	n.d.	n.d.	n.d.	20	20	20	n.d.	n.d.
**antibody fragment**	1471	n.d.	n.d.	n.d.	n.d.	n.d.	n.d.	n.d.	n.d.	20	54	97	393	788
**antibody fragment**	1554	n.d.	n.d.	n.d.	n.d.	n.d.	n.d.	n.d.	n.d.	70	75	n.d.	n.d.	n.d.
**antibody fragment**	1655	n.d.	n.d.	n.d.	n.d.	n.d.	n.d.	n.d.	n.d.	n.d.	n.d.	162	497	1426
**antibody fragment**	1751	n.d.	n.d.	n.d.	n.d.	n.d.	n.d.	n.d.	n.d.	143	195	271	n.d.	n.d.
**antibody fragment**	1892	n.d.	n.d.	n.d.	n.d.	n.d.	n.d.	n.d.	n.d.	n.d.	n.d.	n.d.	n.d.	686
**antibody fragment**	1998	n.d.	n.d.	n.d.	n.d.	n.d.	n.d.	n.d.	n.d.	141	255	352	507	527
**antibody fragment**	2331	n.d.	n.d.	n.d.	n.d.	n.d.	n.d.	n.d.	n.d.	42	82	110	159	245
**antibody fragment**	2403	n.d.	n.d.	n.d.	n.d.	n.d.	n.d.	n.d.	n.d.	20	n.d.	n.d.	n.d.	n.d.
**antibody fragment**	2508	n.d.	n.d.	n.d.	n.d.	n.d.	n.d.	n.d.	n.d.	20	50	82	149	173
**antibody fragment**	2591	n.d.	n.d.	n.d.	n.d.	n.d.	n.d.	n.d.	n.d.	n.d.	20	n.d.	n.d.	n.d.
**antibody fragment**	2647	n.d.	n.d.	n.d.	n.d.	n.d.	n.d.	n.d.	n.d.	n.d.	20	n.d.	n.d.	n.d.
**antibody fragment**	2819	n.d.	n.d.	n.d.	n.d.	n.d.	n.d.	n.d.	n.d.	n.d.	n.d.	54	82	86
**antibody fragment**	3336	n.d.	n.d.	n.d.	n.d.	n.d.	n.d.	n.d.	n.d.	n.d.	n.d.	20	20	20
**antibody fragment**	3461	n.d.	n.d.	n.d.	n.d.	n.d.	n.d.	n.d.	n.d.	n.d.	n.d.	20	20	20

a) Imputed values necessary for optimized Gauss-fit are shown in red and are equal to the background intensity at the given m/z.
b) n.d.: value not determined / ion signal not present.

Table S7. continued

peptide 5, measurement 5

ion / charge state	m/z	ΔCV												
		**2**	**4**	**6**	**8**	**12**	**16**	**20**	**30**	**40**	**50**	**60**	**70**	**80**
**peptide dimer / 1+**	3522.74	5	5	5	5	10	10	10	10	10	10	10	10	10
**peptide 5 / 1+**	1761.87	7	12	9	9	14	18	22	40	67	114	244	316	364
**peptide 5 / 2+**	881.45	95	330	586	953	1748	2953	2604	2853	4909	7857	11720	13183	13547
**peptide 5 / 3+**	588.26	6	8	7	13	17	36	25	27	37	67	69	67	69
**peptide 5 +Na / 3+**	595.39	n.d.	n.d.	n.d.	n.d.	n.d.	n.d.	n.d.	n.d.	n.d.	n.d.	37	26	47
**peptide 5 / 4+**	441.22	5	5	5	5	10	10	10	10	10	10	10	10	10
**antibody+0 pep / 29+**	5054	50	50	50	50	50	50	50	50	50	50	50	50	50
**antibody+0 pep / 28+**	5229	222	388	552	652	791	948	676	451	532	740	944	1050	1080
**antibody+0 pep / 27+**	5424	1359	2166	3024	3492	4224	4730	3669	2632	3131	3896	5295	5612	5807
**antibody+0 pep / 26+**	5634	3236	5211	7041	8091	9075	9968	7996	6697	7615	9554	11922	12868	13124
**antibody+0 pep / 25+**	5860	3476	5371	7179	7941	8839	9741	8012	7076	8347	10242	12871	13912	14085
**antibody+0 pep / 24+**	6103	1887	2792	3610	4034	4501	5009	4153	3978	4932	6058	7651	8235	8049
**antibody+0 pep / 23+**	6371	538	765	987	1138	1352	1438	1347	1426	1853	2372	2854	3150	2978
**antibody+0 pep / 22+**	6655	69	110	140	161	175	217	228	277	409	524	676	755	803
**antibody+0 pep / 21+**	6975	50	50	50	50	50	50	50	50	50	50	50	50	50
**antibody+1 pep/ 29+**	5115	50	50	50	50	50	50	50	50	50	50	50	50	50
**antibody+1 pep/ 28+**	5289	278	436	612	746	889	1009	777	568	629	777	971	1035	1039
**antibody+1 pep/ 27+**	5491	1469	2280	3107	3592	3963	4621	3482	2750	3081	3673	4539	4855	5003
**antibody+1 pep/ 26+**	5701	2865	4683	6269	6856	7517	8140	6645	5552	6137	7397	8911	9247	9318
**antibody+1 pep/ 25+**	5930	2461	3972	5168	5706	6311	6632	5428	5024	5570	6449	7623	8149	8164
**antibody+1 pep/ 24+**	6177	1096	1709	2272	2520	2783	2891	2483	2355	2835	3162	3853	4029	4002
**antibody+1 pep/ 23+**	6445	271	422	512	585	680	703	681	694	897	1022	1243	1392	1277
**antibody+1 pep/ 22+**	6734	39	51	63	81	90	92	117	134	185	245	324	370	395
**antibody+1 pep/ 21+**	7059	50	50	50	50	50	50	50	50	50	50	50	50	50
**antibody+2 peps/ 29+**	5176	50	50	50	50	50	50	50	50	50	50	50	50	50
**antibody+2 peps/ 28+**	5356	107	144	216	260	276	340	243	201	203	239	312	300	328
**antibody+2 peps/ 27+**	5554	421	681	848	966	1047	1202	890	705	762	884	1042	1117	1109
**antibody+2 peps/ 26+**	5768	714	1152	1451	1628	1723	1842	1517	1252	1318	1498	1686	1780	1794
**antibody+2 peps/ 25+**	6000	493	784	1035	1119	1220	1229	1099	935	1024	1078	1294	1288	1241

**antibody+2 peps/ 24+**	6250	188	332	384	428	483	495	444	418	487	519	619	625	588
**antibody+2 peps/ 23+**	6524	53	69	87	101	112	137	134	135	151	204	203	232	222
**antibody+2 peps/ 22+**	6810	14	17	24	25	29	28	37	48	63	76	105	130	132
**antibody+2 peps/ 21+**	7141	50	50	50	50	50	50	50	50	50	50	50	50	50
**antibody fragment**	1000	n.d.	n.d.	n.d.	n.d.	n.d.	n.d.	n.d.	n.d.	n.d.	n.d.	n.d.	20	20
**antibody fragment**	1175	n.d.	n.d.	n.d.	n.d.	n.d.	n.d.	n.d.	n.d.	n.d.	n.d.	n.d.	20	20
**antibody fragment**	1324	n.d.	n.d.	n.d.	n.d.	n.d.	n.d.	n.d.	n.d.	n.d.	n.d.	20	66	163
**antibody fragment**	1440	n.d.	n.d.	n.d.	n.d.	n.d.	n.d.	n.d.	n.d.	20	20	20	n.d.	n.d.
**antibody fragment**	1471	n.d.	n.d.	n.d.	n.d.	n.d.	n.d.	n.d.	n.d.	20	20	115	380	948
**antibody fragment**	1554	n.d.	n.d.	n.d.	n.d.	n.d.	n.d.	n.d.	n.d.	62	71	123	n.d.	n.d.
**antibody fragment**	1655	n.d.	n.d.	n.d.	n.d.	n.d.	n.d.	n.d.	n.d.	n.d.	n.d.	157	549	1794
**antibody fragment**	1751	n.d.	n.d.	n.d.	n.d.	n.d.	n.d.	n.d.	n.d.	116	139	300	n.d.	n.d.
**antibody fragment**	1892	n.d.	n.d.	n.d.	n.d.	n.d.	n.d.	n.d.	n.d.	n.d.	n.d.	n.d.	n.d.	772
**antibody fragment**	1998	n.d.	n.d.	n.d.	n.d.	n.d.	n.d.	n.d.	n.d.	94	210	379	526	580
**antibody fragment**	2189	n.d.	n.d.	n.d.	n.d.	n.d.	n.d.	n.d.	n.d.	44	85	158	265	n.d.
**antibody fragment**	2331	n.d.	n.d.	n.d.	n.d.	n.d.	n.d.	n.d.	n.d.	n.d.	78	135	231	247
**antibody fragment**	2508	n.d.	n.d.	n.d.	n.d.	n.d.	n.d.	n.d.	n.d.	33	46	140	192	214
**antibody fragment**	2558	n.d.	n.d.	n.d.	n.d.	n.d.	n.d.	n.d.	n.d.	20	20	n.d.	n.d.	n.d.
**antibody fragment**	2591	n.d.	n.d.	n.d.	n.d.	n.d.	n.d.	n.d.	n.d.	20	20	n.d.	n.d.	n.d.
**antibody fragment**	2647	n.d.	n.d.	n.d.	n.d.	n.d.	n.d.	n.d.	n.d.	20	20	n.d.	n.d.	n.d.
**antibody fragment**	2819	n.d.	n.d.	n.d.	n.d.	n.d.	n.d.	n.d.	n.d.	n.d.	n.d.	49	89	109
**antibody fragment**	3336	n.d.	n.d.	n.d.	n.d.	n.d.	n.d.	n.d.	n.d.	n.d.	n.d.	20	20	20
**antibody fragment**	3461	n.d.	n.d.	n.d.	n.d.	n.d.	n.d.	n.d.	n.d.	n.d.	n.d.	20	20	20

a) Imputed values necessary for optimized Gauss-fit are shown in red and are equal to the background intensity at the given m/z.
b) n.d.: value not determined / ion signal not present.

Table S8. Ion species, charge states, m/z values and intensities for anti-Troponin I antibody complexed with Troponin I peptide 6 at measured collision cell voltage differences. [a,b)]

peptide 6, measurement 1

ion / charge state	m/z	ΔCV										
		4	8	12	16	20	30	40	50	60	70	80
**peptide dimer / 1+**	3512.82	5	10	10	10	10	10	10	10	10	10	10
**peptide 6 / 1+**	1757.95	6	11	11	10	13	43	85	114	189	248	315
**peptide 6 / 2+**	878.94	155	345	714	1342	2466	4479	7350	9226	13505	15666	15398
**peptide 6 / 3+**	586.37	102	242	428	801	1374	2061	3425	4118	5442	5440	5034
**peptide 6 / 4+**	439.98	5	10	10	10	10	10	10	10	10	10	10
**antibody+0 pep / 28+**	5233	20	20	20	20	20	20	20	20	20	20	20
**antibody+0 pep / 27+**	5424	141	283	275	427	651	546	644	510	669	781	637
**antibody+0 pep / 26+**	5633	866	1811	1730	2432	3443	3025	3409	2888	3792	4194	3779
**antibody+0 pep / 25+**	5862	2046	4386	3977	5103	7108	6493	6976	6140	8039	8613	8197
**antibody+0 pep / 24+**	6103	2285	4894	4253	5115	6768	6533	6992	6193	8033	8205	8051
**antibody+0 pep / 23+**	6373	1308	2617	2339	2721	3430	3374	3604	3438	4455	4627	4491
**antibody+0 pep / 22+**	6664	384	779	688	772	960	987	1141	1200	1510	1636	1555
**antibody+0 pep / 21+**	6977	20	20	20	20	20	20	20	20	20	20	20
**antibody+1 pep/ 28+**	5295	20	20	20	20	20	20	20	20	20	20	20
**antibody+1 pep/ 27+**	5489	265	570	611	811	1099	903	1041	807	975	1078	905
**antibody+1 pep/ 26+**	5700	1340	2976	2962	3551	4656	4012	4196	3496	4185	4460	4000
**antibody+1 pep/ 25+**	5929	2534	5373	5416	6099	7440	6769	6825	6073	7040	7388	6800
**antibody+1 pep/ 24+**	6177	2481	5218	5106	5446	6258	5890	5914	5277	6127	6383	5974
**antibody+1 pep/ 23+**	6446	1173	2437	2380	2468	2684	2589	2690	2482	2935	3019	2899
**antibody+1 pep/ 22+**	6743	337	688	631	624	699	704	737	797	886	983	939
**antibody+1 pep/ 21+**	7059	20	20	20	20	20	20	20	20	20	20	20
**antibody+2 peps/ 28+**	5356	20	20	20	20	20	20	20	20	20	20	20
**antibody+2 peps/ 27+**	5553	139	319	327	391	429	368	375	323	336	357	307
**antibody+2 peps/ 26+**	5766	562	1163	1220	1336	1434	1285	1269	1103	1072	1140	1003
**antibody+2 peps/ 25+**	5997	925	1911	2070	2004	2101	1931	1831	1687	1564	1598	1513
**antibody+2 peps/ 24+**	6249	771	1689	1711	1621	1592	1492	1402	1273	1279	1280	1145
**antibody+2 peps/ 23+**	6522	312	642	662	657	570	567	546	518	509	518	482
**antibody+2 peps/ 22+**	6819	98	177	176	167	137	153	154	160	163	174	177
**antibody+2 peps/ 21+**	7141	20	20	20	20	20	20	20	20	20	20	20

**antibody fragment**	1000	n.d.	n.d.	n.d.	n.d.	n.d.	n.d.	n.d.	n.d.	n.d.	10	10
**antibody fragment**	1204	n.d.	n.d.	n.d.	n.d.	n.d.	n.d.	10	10	10	10	10
**antibody fragment**	1324	n.d.	n.d.	n.d.	n.d.	n.d.	n.d.	10	10	10	91	185
**antibody fragment**	1471	n.d.	n.d.	n.d.	n.d.	n.d.	n.d.	n.d.	34	164	602	1297
**antibody fragment**	1554	n.d.	n.d.	n.d.	n.d.	n.d.	n.d.	60	50	n.d.	n.d.	n.d.
**antibody fragment**	1655	n.d.	n.d.	n.d.	n.d.	n.d.	n.d.	n.d.	n.d.	260	963	2450
**antibody fragment**	1751	n.d.	n.d.	n.d.	n.d.	n.d.	n.d.	123	116	n.d.	n.d.	n.d.
**antibody fragment**	1892	n.d.	n.d.	n.d.	n.d.	n.d.	n.d.	n.d.	n.d.	n.d.	n.d.	1031
**antibody fragment**	1998	n.d.	n.d.	n.d.	n.d.	n.d.	n.d.	133	202	390	690	704
**antibody fragment**	2160	n.d.	n.d.	n.d.	n.d.	n.d.	n.d.	39	105	251	394	585
**antibody fragment**	2331	n.d.	n.d.	n.d.	n.d.	n.d.	n.d.	30	59	117	182	271
**antibody fragment**	2392	n.d.	n.d.	n.d.	n.d.	n.d.	n.d.	10	n.d.	n.d.	n.d.	n.d.
**antibody fragment**	2508	n.d.	n.d.	n.d.	n.d.	n.d.	n.d.	10	15	32	66	n.d.
**antibody fragment**	2819	n.d.	n.d.	n.d.	n.d.	n.d.	n.d.	n.d.	10	10	10	10
**antibody fragment**	3336	n.d.	n.d.	n.d.	n.d.	n.d.	n.d.	n.d.	10	10	10	10

a) Imputed values necessary for optimized Gauss-fit are shown in red and are equal to the background intensity at the given m/z.
b) n.d.: value not determined / ion signal not present.

Table S8. continued

peptide 6, measurement 2

ion / charge state	m/z	ΔCV										
		4	8	12	16	20	30	40	50	60	70	80
**peptide dimer / 1+**	3512.82	5	5	10	10	10	10	10	10	10	10	10
**peptide 6 / 1+**	1756.91	4	8	12	8	10	11	45	63	90	145	187
**peptide 6 / 2+**	878.97	35	149	342	716	1168	1573	3977	5988	8278	9190	9339
**peptide 6 / 3+**	586.39	21	75	169	342	476	630	1407	2227	2444	2430	2398
**peptide 6 / 4+**	439.98	5	10	10	10	10	10	10	10	10	10	10
**antibody+0 pep / 29+**	5052	50	50	50	50	50	50	50	50	50	50	50
**antibody+0 pep / 28+**	5236	162	326	412	459	449	473	627	621	574	535	502
**antibody+0 pep / 27+**	5430	539	1253	1453	1536	1467	1668	2114	2104	2050	1986	1898
**antibody+0 pep / 26+**	5637	1257	2812	2921	3068	2980	3339	4197	4340	4431	4344	4219
**antibody+0 pep / 25+**	5858	1395	3136	3184	3283	3327	3545	4160	4573	4927	5178	5134
**antibody+0 pep / 24+**	6102	924	2073	2021	2058	2036	2185	2571	2900	3321	3567	3478
**antibody+0 pep / 23+**	6366	372	853	814	755	815	870	1039	1226	1444	1594	1612
**antibody+0 pep / 22+**	6660	50	50	50	50	50	50	50	50	50	50	50
**antibody+1 pep/ 29+**	5112	50	50	50	50	50	50	50	50	50	50	50
**antibody+1 pep/ 28+**	5301	210	454	550	632	617	634	705	683	607	543	483
**antibody+1 pep/ 27+**	5494	727	1661	1923	2055	2023	2012	2425	2214	2099	1924	1794
**antibody+1 pep/ 26+**	5698	1479	3299	3476	3697	3838	3757	4094	4237	4149	4193	4006
**antibody+1 pep/ 25+**	5927	1352	3076	3170	3370	3455	3474	3678	3815	4067	4161	3978
**antibody+1 pep/ 24+**	6175	796	1724	1706	1745	1875	1916	2043	2148	2383	2457	2407
**antibody+1 pep/ 23+**	6443	279	615	557	545	578	633	688	781	886	955	1016
**antibody+1 pep/ 22+**	6738	50	50	50	50	50	50	50	50	50	50	50
**antibody+2 peps/ 29+**	5171	50	50	50	50	50	50	50	50	50	50	50
**antibody+2 peps/ 28+**	5365	121	255	298	329	353	260	250	247	195	176	180
**antibody+2 peps/ 27+**	5552	392	887	951	993	1236	892	778	797	692	677	595
**antibody+2 peps/ 26+**	5765	659	1501	1560	1686	2002	1445	1252	1264	1212	1143	1026
**antibody+2 peps/ 25+**	5995	564	1237	1173	1246	1512	1117	947	1002	973	970	905
**antibody+2 peps/ 24+**	6247	264	612	596	568	638	529	470	497	515	502	498
**antibody+2 peps/ 23+**	6517	94	197	149	153	158	162	144	177	189	199	212
**antibody+2 peps/ 22+**	6816	50	50	50	50	50	50	50	50	50	50	50

**antibody fragment**	1000	n.d.	n.d.	n.d.	n.d.	n.d.	n.d.	n.d.	20	20	20	20
**antibody fragment**	1324	n.d.	n.d.	n.d.	n.d.	n.d.	n.d.	n.d.	n.d.	54	127	303
**antibody fragment**	1471	n.d.	n.d.	n.d.	n.d.	n.d.	n.d.	n.d.	n.d.	97	226	550
**antibody fragment**	1554	n.d.	n.d.	n.d.	n.d.	n.d.	n.d.	n.d.	40	n.d.	n.d.	n.d.
**antibody fragment**	1655	n.d.	n.d.	n.d.	n.d.	n.d.	n.d.	n.d.	n.d.	n.d.	227	606
**antibody fragment**	1751	n.d.	n.d.	n.d.	n.d.	n.d.	n.d.	n.d.	68	120	187	n.d.
**antibody fragment**	1851	n.d.	n.d.	n.d.	n.d.	n.d.	n.d.	n.d.	n.d.	n.d.	96	n.d.
**antibody fragment**	1892	n.d.	n.d.	n.d.	n.d.	n.d.	n.d.	n.d.	n.d.	n.d.	86	219
**antibody fragment**	1998	n.d.	n.d.	n.d.	n.d.	n.d.	n.d.	n.d.	n.d.	95	n.d.	n.d.
**antibody fragment**	2160	n.d.	n.d.	n.d.	n.d.	n.d.	n.d.	n.d.	20	20	n.d.	n.d.
**antibody fragment**	2189	n.d.	n.d.	n.d.	n.d.	n.d.	n.d.	n.d.	20	20	n.d.	n.d.
**antibody fragment**	2207	n.d.	n.d.	n.d.	n.d.	n.d.	n.d.	n.d.	n.d.	20	20	n.d.
**antibody fragment**	2331	n.d.	n.d.	n.d.	n.d.	n.d.	n.d.	n.d.	n.d.	20	71	20
**antibody fragment**	2508	n.d.	20	20	20	20	20	20	20	n.d.	100	20
**antibody fragment**	2819	n.d.	20	20	20	20	20	20	20	n.d.	42	n.d.
**antibody fragment**	3336	n.d.	79	64	n.d.	n.d.	n.d.	n.d.	n.d.	63	20	n.d.
**antibody fragment**	3461	n.d.	n.d.	n.d.	n.d.	n.d.	n.d.	n.d.	n.d.	n.d.	41	n.d.
**antibody fragment**	3590	n.d.	65	57	67	65	65	68	68	76	63	n.d.
**antibody fragment**	3728	n.d.	n.d.	n.d.	n.d.	n.d.	n.d.	n.d.	n.d.	n.d.	54	n.d.
**antibody fragment**	3886	n.d.	n.d.	n.d.	n.d.	n.d.	n.d.	n.d.	n.d.	50	n.d.	n.d.
**antibody fragment**	4200	n.d.	20	20	20	20	20	20	20	20	20	n.d.
**antibody fragment**	4400	n.d.	20	20	20	20	20	20	20	20	20	n.d.

a) Imputed values necessary for optimized Gauss-fit are shown in red and are equal to the background intensity at the given m/z.
b) n.d.: value not determined / ion signal not present.

Table S8. continued

peptide 6, measurement 3

ion / charge state	m/z	ΔCV												
		**2**	**4**	**6**	**8**	**12**	**16**	**20**	**30**	**40**	**50**	**60**	**70**	**80**
**peptide dimer / 1+**	3512.82	5	5	10	10	10	10	10	10	10	10	10	10	10
**peptide 6 / 1+**	1756.91	7	10	11	12	15	20	21	77	191	390	441	559	485
**peptide 6 / 2+**	878.97	63	108	242	655	1573	3014	4865	8639	13170	29572	37478	44275	41327
**peptide 6 / 3+**	586.39	87	138	365	574	1202	2142	3205	5014	6824	13908	15507	16916	14287
**peptide 6 / 4+**	439.98	5	5	10	10	10	10	10	10	10	10	10	10	10
**antibody+0 pep / 29+**	5052	50	50	50	50	50	50	50	50	50	50	50	50	50
**antibody+0 pep / 28+**	5236	137	223	244	321	432	568	688	740	931	3002	3583	4019	3633
**antibody+0 pep / 27+**	5430	848	1567	1914	2421	3164	3897	4274	4786	5572	11255	12743	13825	12901
**antibody+0 pep / 26+**	5637	2372	4300	5519	6520	8318	9814	10633	11777	13052	19995	21793	21907	21646
**antibody+0 pep / 25+**	5858	2747	4854	6005	7396	9293	10885	11488	12767	14295	17772	18641	17956	17818
**antibody+0 pep / 24+**	6102	1739	2837	3515	4411	5432	6459	6756	7631	8478	9380	9630	8822	8983
**antibody+0 pep / 23+**	6366	621	1057	1235	1541	2001	2276	2553	2861	3494	3464	3601	3325	3430
**antibody+0 pep / 22+**	6660	50	50	50	50	50	50	50	50	50	50	50	50	50
**antibody+1 pep/ 29+**	5112	50	50	50	50	50	50	50	50	50	50	50	50	50
**antibody+1 pep/ 28+**	5301	209	379	446	590	749	936	1064	1260	1386	3334	3709	4051	3553
**antibody+1 pep/ 27+**	5494	1205	2298	2713	3425	4253	5036	5519	5989	6637	10273	10980	11211	10453
**antibody+1 pep/ 26+**	5698	2746	4857	6091	7305	9013	10655	11034	11850	12467	15205	15539	15003	14309
**antibody+1 pep/ 25+**	5927	2518	4573	5491	6689	8296	9547	9906	10309	10787	11164	11245	10055	10048
**antibody+1 pep/ 24+**	6175	1330	2233	2745	3364	4186	4807	5030	5270	5708	5181	5014	4491	4417
**antibody+1 pep/ 23+**	6443	415	709	823	1075	1289	1508	1551	1729	1963	1710	1727	1565	1562
**antibody+1 pep/ 22+**	6738	50	50	50	50	50	50	50	50	50	50	50	50	50
**antibody+2 peps/ 29+**	5171	50	50	50	50	50	50	50	50	50	50	50	50	50
**antibody+2 peps/ 28+**	5365	89	176	214	271	348	410	420	475	531	969	1003	1094	962
**antibody+2 peps/ 27+**	5552	459	760	970	1169	1440	1647	1763	1847	1872	2415	2353	2511	2161
**antibody+2 peps/ 26+**	5765	800	1449	1747	2085	2502	2944	2934	2931	2920	3013	2952	2732	2564
**antibody+2 peps/ 25+**	5995	652	1115	1342	1713	1929	2140	2185	2204	2225	1987	1812	1662	1565
**antibody+2 peps/ 24+**	6247	288	521	617	725	894	1043	1044	1052	1075	874	814	807	679
**antibody+2 peps/ 23+**	6517	94	144	170	205	252	303	306	339	369	328	339	362	336

**antibody+2 peps/ 22+**	6816	50	50	50	50	50	50	50	50	50	50	50	50	50
**antibody fragment**	1000	n.d.	n.d.	n.d.	n.d.	n.d.	n.d.	n.d.	n.d.	n.d.	20	20	20	20
**antibody fragment**	1324	n.d.	n.d.	n.d.	n.d.	n.d.	n.d.	n.d.	n.d.	20	20	20	166	338
**antibody fragment**	1471	n.d.	n.d.	n.d.	n.d.	n.d.	n.d.	n.d.	n.d.	20	124	269	981	2041
**antibody fragment**	1554	n.d.	n.d.	n.d.	n.d.	n.d.	n.d.	n.d.	n.d.	132	211	n.d.	n.d.	n.d.
**antibody fragment**	1655	n.d.	n.d.	n.d.	n.d.	n.d.	n.d.	n.d.	n.d.	n.d.	n.d.	437	1256	2979
**antibody fragment**	1751	n.d.	n.d.	n.d.	n.d.	n.d.	n.d.	n.d.	n.d.	235	414	542	n.d.	n.d.
**antibody fragment**	1892	n.d.	n.d.	n.d.	n.d.	n.d.	n.d.	n.d.	n.d.	n.d.	n.d.	n.d.	n.d.	1393
**antibody fragment**	1998	n.d.	n.d.	n.d.	n.d.	n.d.	n.d.	n.d.	n.d.	227	511	717	830	836
**antibody fragment**	2331	n.d.	n.d.	n.d.	n.d.	n.d.	n.d.	n.d.	n.d.	61	213	308	408	495
**antibody fragment**	2391	n.d.	n.d.	n.d.	n.d.	n.d.	n.d.	n.d.	n.d.	20	n.d.	n.d.	n.d.	n.d.
**antibody fragment**	2508	n.d.	n.d.	n.d.	n.d.	n.d.	n.d.	n.d.	n.d.	20	83	133	177	245
**antibody fragment**	2819	n.d.	n.d.	n.d.	n.d.	n.d.	n.d.	n.d.	n.d.	n.d.	20	20	20	20
**antibody fragment**	3336	n.d.	n.d.	n.d.	n.d.	n.d.	n.d.	n.d.	n.d.	n.d.	20	20	20	20

a) Imputed values necessary for optimized Gauss-fit are shown in red and are equal to the background intensity at the given m/z.
b) n.d.: value not determined / ion signal not present.

TableS 8. continued

peptide 6, measurement 4

ion / charge state	m/z	ΔCV												
		2	4	6	8	12	16	20	30	40	50	60	70	80
peptide dimer / 1+	3512.82	5	5	10	10	10	10	10	10	10	10	10	10	10
peptide 6 / 1+	1756.91	8	10	11	11	14	16	25	54	191	248	292	411	311
peptide 6 / 2+	878.97	106	171	585	1001	1915	3565	5325	10738	16102	20261	23692	25560	24250
peptide 6 / 3+	586.39	128	384	629	879	1466	2646	3823	6138	8564	9678	10013	9786	8539
peptide 6 / 4+	439.98	5	5	10	10	10	10	10	10	10	10	10	10	10
antibody+0 pep / 29+	5052	50	50	50	50	50	50	50	50	50	50	50	50	50
antibody+0 pep / 28+	5236	352	486	679	918	1246	1604	1886	2260	2615	2548	2794	2811	2299
antibody+0 pep / 27+	5430	1801	2591	3282	4313	5133	6183	6912	8064	9317	9577	10074	10267	9072
antibody+0 pep / 26+	5637	3534	4949	6588	8269	9526	11225	12044	13489	15618	16379	17428	17213	15852
antibody+0 pep / 25+	5858	3156	4281	5920	7177	8184	9196	9577	10966	12661	13665	14615	14513	13735
antibody+0 pep / 24+	6102	1473	1972	2701	3330	3634	4134	4331	4985	5890	6688	7412	7167	7225
antibody+0 pep / 23+	6366	407	499	749	842	1016	1114	1271	1659	2125	2484	2791	2747	2859
antibody+0 pep / 22+	6660	50	50	50	50	50	50	50	50	50	50	50	50	50
antibody+1 pep/ 29+	5112	50	50	50	50	50	50	50	50	50	50	50	50	50
antibody+1 pep/ 28+	5301	586	843	1059	1419	1762	2260	2469	2790	3102	2810	2938	2968	2368
antibody+1 pep/ 27+	5494	2286	3059	4059	5197	5973	7015	7404	8168	8749	8648	8746	8587	7644
antibody+1 pep/ 26+	5698	3510	4857	6636	8073	9072	10064	10603	11407	12061	12293	12435	12383	11262
antibody+1 pep/ 25+	5927	2554	3494	4798	5689	6541	6886	7025	7617	8195	8643	8558	8495	8078
antibody+1 pep/ 24+	6175	1022	1466	1917	2329	2509	2745	2796	3036	3412	3738	3850	3749	3819
antibody+1 pep/ 23+	6443	232	319	432	510	628	658	688	816	1007	1158	1292	1264	1308
antibody+1 pep/ 22+	6738	50	50	50	50	50	50	50	50	50	50	50	50	50
antibody+2 peps/ 29+	5171	50	50	50	50	50	50	50	50	50	50	50	50	50
antibody+2 peps/ 28+	5365	232	350	435	545	624	823	815	950	909	814	769	769	664
antibody+2 peps/ 27+	5552	738	1037	1301	1649	1815	2016	2159	2192	2127	2048	1941	1905	1699
antibody+2 peps/ 26+	5765	939	1383	1780	2119	2284	2487	2549	2611	2479	2456	2364	2194	2087
antibody+2 peps/ 25+	5995	573	800	1126	1308	1384	1483	1469	1485	1459	1401	1451	1343	1267
antibody+2 peps/ 24+	6247	204	284	379	448	521	547	532	569	565	612	622	584	565
antibody+2 peps/ 23+	6517	51	65	89	115	127	120	146	190	185	223	235	239	243

**antibody+2 peps/ 22+**	6816	50	50	50	50	50	50	50	50	50	50	50	50	50
**antibody fragment**	1000	n.d.	n.d.	n.d.	n.d.	n.d.	n.d.	n.d.	n.d.	n.d.	20	20	20	20
**antibody fragment**	1324	n.d.	n.d.	n.d.	n.d.	n.d.	n.d.	n.d.	n.d.	20	20	20	94	225
**antibody fragment**	1471	n.d.	n.d.	n.d.	n.d.	n.d.	n.d.	n.d.	n.d.	20	77	227	617	1383
**antibody fragment**	1554	n.d.	n.d.	n.d.	n.d.	n.d.	n.d.	n.d.	n.d.	109	148	n.d.	n.d.	n.d.
**antibody fragment**	1655	n.d.	n.d.	n.d.	n.d.	n.d.	n.d.	n.d.	n.d.	n.d.	n.d.	351	930	2082
**antibody fragment**	1751	n.d.	n.d.	n.d.	n.d.	n.d.	n.d.	n.d.	n.d.	231	289	398	n.d.	n.d.
**antibody fragment**	1892	n.d.	n.d.	n.d.	n.d.	n.d.	n.d.	n.d.	n.d.	n.d.	n.d.	n.d.	n.d.	985
**antibody fragment**	1998	n.d.	n.d.	n.d.	n.d.	n.d.	n.d.	n.d.	n.d.	196	360	554	649	755
**antibody fragment**	2174	n.d.	n.d.	n.d.	n.d.	n.d.	n.d.	n.d.	n.d.	40	n.d.	n.d.	n.d.	n.d.
**antibody fragment**	2207	n.d.	n.d.	n.d.	n.d.	n.d.	n.d.	n.d.	n.d.	20	n.d.	n.d.	n.d.	n.d.
**antibody fragment**	2331	n.d.	n.d.	n.d.	n.d.	n.d.	n.d.	n.d.	n.d.	20	142	219	338	344
**antibody fragment**	2508	n.d.	n.d.	n.d.	n.d.	n.d.	n.d.	n.d.	n.d.	n.d.	80	136	193	260
**antibody fragment**	2819	n.d.	n.d.	n.d.	n.d.	n.d.	n.d.	n.d.	n.d.	n.d.	20	20	20	20
**antibody fragment**	3336	n.d.	n.d.	n.d.	n.d.	n.d.	n.d.	n.d.	n.d.	n.d.	20	20	20	20

a) Imputed values necessary for optimized Gauss-fit are shown in red and are equal to the background intensity at the given m/z.
b) n.d.: value not determined / ion signal not present.

Table S9. Ion species, charge states, m/z values and intensities for anti-Troponin I antibody complexed with Troponin I peptide 7 at measured collision cell voltage differences. [a,b)]

peptide 7, measurement 1

ion / charge state	m/z	ΔCV												
		2	4	6	8	12	16	20	30	40	50	60	70	80
**peptide dimer / 1+**	3510.42	5	5	10	10	10	10	10	10	10	10	10	10	10
**peptide 6 / 1+**	1755.71	8	10	11	11	14	16	25	54	191	248	292	411	311
**peptide 6 / 2+**	878.47	106	171	585	1001	1915	3565	5325	10738	16102	20261	23692	25560	24250
**peptide 6 / 3+**	585.98	128	384	629	879	1466	2646	3823	6138	8564	9678	10013	9786	8539
**peptide 6 / 4+**	439.68	5	5	10	10	10	10	10	10	10	10	10	10	10
**antibody+0 pep / 29+**	5048	50	50	50	50	50	50	50	50	50	50	50	50	50
**antibody+0 pep / 28+**	5228	1277	2473	3461	4771	5381	5372	5798	5934	5740	5941	5671	5876	5606
**antibody+0 pep / 27+**	5421	4647	8452	11526	14457	15670	16272	17236	17359	17481	18093	18032	18696	17965
**antibody+0 pep / 26+**	5630	7117	12418	16519	19665	21288	21857	22960	23456	24013	25793	25641	26606	26086
**antibody+0 pep / 25+**	5855	4976	8279	11124	12907	13845	14353	14930	15047	16368	17720	18236	18558	18671
**antibody+0 pep / 24+**	6099	1781	2880	3977	4438	4710	5026	5115	5351	5868	6297	6670	6828	6939
**antibody+0 pep / 23+**	6365	353	551	733	826	870	952	992	987	1211	1329	1487	1554	1654
**antibody+0 pep / 22+**	6653	50	50	50	50	50	50	50	50	50	50	50	50	50
**antibody+1 pep/ 29+**	5107	20	50	50	50	50	50	50	50	50	50	50	50	50
**antibody+1 pep/ 28+**	5291	151	302	397	493	550	569	582	606	574	529	542	569	513
**antibody+1 pep/ 27+**	5485	459	855	1107	1403	1392	1419	1474	1463	1449	1440	1445	1456	1406
**antibody+1 pep/ 26+**	5698	622	1069	1408	1665	1686	1792	1808	1757	1779	1801	1839	1836	1833
**antibody+1 pep/ 25+**	5923	409	698	884	974	1019	1037	1078	1041	1130	1107	1159	1168	1184
**antibody+1 pep/ 24+**	6171	146	231	295	339	344	372	408	384	431	454	491	494	530
**antibody+1 pep/ 23+**	6439	39	51	74	75	86	93	109	110	142	210	215	240	256
**antibody+1 pep/ 22+**	6732	20	50	50	50	50	50	50	50	50	50	50	50	50
**antibody+2 peps/ 29+**	5175	20	20	20	20	20	50	50	50	50	50	50	50	50
**antibody+2 peps/ 28+**	5360	48	98	121	177	151	168	208	237	219	236	230	265	266
**antibody+2 peps/ 27+**	5559	78	126	177	214	262	270	321	288	313	357	276	360	283
**antibody+2 peps/ 26+**	5772	91	172	210	267	265	292	285	310	286	301	304	299	279
**antibody+2 peps/ 25+**	6003	74	104	150	183	195	234	229	242	259	270	265	218	213
**antibody+2 peps/ 24+**	6253	39	64	77	101	119	131	142	164	184	209	197	178	155

**antibody+2 peps/ 23+**	6525	16	22	34	40	45	51	72	83	95	106	117	102	91
**antibody+2 peps/ 22+**	6822	20	20	20	20	20	50	50	50	50	50	50	50	50
**antibody fragment**	1000	n.d.	n.d.	n.d.	n.d.	n.d.	n.d.	n.d.	n.d.	n.d.	n.d.	20	20	20
**antibody fragment**	1200	n.d.	n.d.	n.d.	n.d.	n.d.	n.d.	n.d.	n.d.	n.d.	20	20	20	20
**antibody fragment**	1324	n.d.	n.d.	n.d.	n.d.	n.d.	n.d.	n.d.	n.d.	20	20	29	72	132
**antibody fragment**	1471	n.d.	n.d.	n.d.	n.d.	n.d.	n.d.	n.d.	n.d.	20	55	121	383	1046
**antibody fragment**	1554	n.d.	n.d.	n.d.	n.d.	n.d.	n.d.	n.d.	n.d.	79	n.d.	n.d.	n.d.	n.d.
**antibody fragment**	1655	n.d.	n.d.	n.d.	n.d.	n.d.	n.d.	n.d.	n.d.	n.d.	n.d.	232	713	1812
**antibody fragment**	1751	n.d.	n.d.	n.d.	n.d.	n.d.	n.d.	n.d.	n.d.	176	203	259	n.d.	n.d.
**antibody fragment**	1892	n.d.	n.d.	n.d.	n.d.	n.d.	n.d.	n.d.	n.d.	n.d.	n.d.	n.d.	n.d.	875
**antibody fragment**	1998	n.d.	n.d.	n.d.	n.d.	n.d.	n.d.	n.d.	n.d.	166	257	414	492	571
**antibody fragment**	2331	n.d.	n.d.	n.d.	n.d.	n.d.	n.d.	n.d.	n.d.	53	104	166	261	247
**antibody fragment**	2391	n.d.	n.d.	n.d.	n.d.	n.d.	n.d.	n.d.	n.d.	20	n.d.	n.d.	n.d.	n.d.
**antibody fragment**	2508	n.d.	n.d.	n.d.	n.d.	n.d.	n.d.	n.d.	n.d.	20	70	146	180	218
**antibody fragment**	2819	n.d.	n.d.	n.d.	n.d.	n.d.	n.d.	n.d.	n.d.	n.d.	31	46	73	92
**antibody fragment**	3336	n.d.	n.d.	n.d.	n.d.	n.d.	n.d.	n.d.	n.d.	n.d.	20	20	20	20
**antibody fragment**	3461	n.d.	n.d.	n.d.	n.d.	n.d.	n.d.	n.d.	n.d.	n.d.	20	20	20	20

a) Imputed values necessary for optimized Gauss-fit are shown in red and are equal to the background intensity at the given m/z.
b) n.d.: value not determined / ion signal not present.

TableS 9. continued

peptide 7, measurement 2

ion / charge state	m/z	ΔCV												
		**2**	**4**	**6**	**8**	**12**	**16**	**20**	**30**	**40**	**50**	**60**	**70**	**80**
**peptide dimer / 1+**	3510.42	5	5	10	10	10	10	10	10	10	10	10	10	10
**peptide 6 / 1+**	1755.71	9	7	13	16	12	21	30	78	183	230	252	340	334
**peptide 6 / 2+**	878.47	54	78	168	225	297	423	714	928	1401	1471	1698	1956	2057
**peptide 6 / 3+**	585.98	5	10	14	11	11	9	10	11	20	20	18	15	15
**peptide 6 / 4+**	439.68	5	5	10	10	10	10	10	10	10	10	10	10	10
**antibody+0 pep / 29+**	5048	50	50	50	50	50	50	50	50	50	50	50	50	50
**antibody+0 pep / 28+**	5228	1774	2019	3007	3478	5023	5904	6948	6623	6763	6154	6758	7505	7708
**antibody+0 pep / 27+**	5421	6016	7481	10474	11689	15422	17376	19777	19437	19944	19329	20150	22291	22874
**antibody+0 pep / 26+**	5630	8754	11266	15336	16942	21160	23133	25513	26325	26873	27478	28226	30939	31509
**antibody+0 pep / 25+**	5855	5846	7859	10893	11947	14153	14825	16007	17247	18140	19100	19596	21391	21293
**antibody+0 pep / 24+**	6099	2123	2843	4021	4269	4966	5007	5363	5859	6507	7121	7202	7901	7696
**antibody+0 pep / 23+**	6365	433	574	767	856	901	974	1056	1201	1296	1598	1610	1788	1786
**antibody+0 pep / 22+**	6653	50	50	50	50	50	50	50	50	50	50	50	50	50
**antibody+1 pep/ 29+**	5107	50	50	50	50	50	50	50	50	50	50	50	50	50
**antibody+1 pep/ 28+**	5291	242	268	385	433	542	617	712	674	665	619	633	673	707
**antibody+1 pep/ 27+**	5485	690	792	1061	1183	1463	1564	1709	1629	1652	1594	1626	1715	1740
**antibody+1 pep/ 26+**	5698	807	996	1357	1459	1751	1864	2101	2020	2032	1981	1987	2108	2132
**antibody+1 pep/ 25+**	5923	491	619	860	935	1080	1091	1180	1226	1219	1257	1236	1356	1363
**antibody+1 pep/ 24+**	6171	191	248	347	356	385	403	440	465	465	506	526	573	567
**antibody+1 pep/ 23+**	6439	50	64	94	87	87	92	120	152	193	233	244	325	351
**antibody+1 pep/ 22+**	6732	50	50	50	50	50	50	50	50	50	50	50	50	50
**antibody+2 peps/ 29+**	5175	20	20	20	20	50	50	50	50	50	50	50	50	50
**antibody+2 peps/ 28+**	5360	92	122	119	149	195	239	276	291	236	248	254	342	294
**antibody+2 peps/ 27+**	5559	125	147	172	180	280	319	322	373	314	341	382	394	415
**antibody+2 peps/ 26+**	5772	137	169	201	230	279	303	355	363	329	331	323	323	296
**antibody+2 peps/ 25+**	6003	87	117	159	189	198	238	248	283	275	281	288	267	239
**antibody+2 peps/ 24+**	6253	53	67	85	100	108	138	149	181	190	213	224	203	190
**antibody+2 peps/ 23+**	6525	23	33	36	36	49	62	70	96	111	139	138	101	115

**antibody+2 peps/ 22+**	6822	20	20	20	20	50	50	50	50	50	50	50	50	50
**antibody fragment**	1000	n.d.	n.d.	n.d.	n.d.	n.d.	n.d.	n.d.	n.d.	n.d.	n.d.	20	20	20
**antibody fragment**	1200	n.d.	n.d.	n.d.	n.d.	n.d.	n.d.	n.d.	n.d.	n.d.	20	20	20	20
**antibody fragment**	1324	n.d.	n.d.	n.d.	n.d.	n.d.	n.d.	n.d.	n.d.	20	20	n.d.	83	306
**antibody fragment**	1471	n.d.	n.d.	n.d.	n.d.	n.d.	n.d.	n.d.	n.d.	20	62	167	599	1561
**antibody fragment**	1554	n.d.	n.d.	n.d.	n.d.	n.d.	n.d.	n.d.	n.d.	68	91	n.d.	n.d.	n.d.
**antibody fragment**	1655	n.d.	n.d.	n.d.	n.d.	n.d.	n.d.	n.d.	n.d.	n.d.	n.d.	264	969	2605
**antibody fragment**	1751	n.d.	n.d.	n.d.	n.d.	n.d.	n.d.	n.d.	n.d.	196	238	n.d.	n.d.	n.d.
**antibody fragment**	1892	n.d.	n.d.	n.d.	n.d.	n.d.	n.d.	n.d.	n.d.	n.d.	n.d.	n.d.	n.d.	1118
**antibody fragment**	1998	n.d.	n.d.	n.d.	n.d.	n.d.	n.d.	n.d.	n.d.	200	340	471	663	717
**antibody fragment**	2006	n.d.	n.d.	n.d.	n.d.	n.d.	n.d.	n.d.	n.d.	n.d.	n.d.	n.d.	n.d.	n.d.
**antibody fragment**	2160	n.d.	n.d.	n.d.	n.d.	n.d.	n.d.	n.d.	n.d.	n.d.	n.d.	n.d.	n.d.	n.d.
**antibody fragment**	2174	n.d.	n.d.	n.d.	n.d.	n.d.	n.d.	n.d.	n.d.	n.d.	n.d.	n.d.	n.d.	n.d.
**antibody fragment**	2331	n.d.	n.d.	n.d.	n.d.	n.d.	n.d.	n.d.	n.d.	53	127	206	348	393
**antibody fragment**	2391	n.d.	n.d.	n.d.	n.d.	n.d.	n.d.	n.d.	n.d.	20	n.d.	n.d.	n.d.	n.d.
**antibody fragment**	2508	n.d.	n.d.	n.d.	n.d.	n.d.	n.d.	n.d.	n.d.	20	65	142	208	268
**antibody fragment**	2819	n.d.	n.d.	n.d.	n.d.	n.d.	n.d.	n.d.	n.d.	n.d.	20	20	20	20
**antibody fragment**	3336	n.d.	n.d.	n.d.	n.d.	n.d.	n.d.	n.d.	n.d.	n.d.	20	20	20	20

a) Imputed values necessary for optimized Gauss-fit are shown in red and are equal to the background intensity at the given m/z.
b) n.d.: value not determined / ion signal not present.

TableS 10. Apex heights of Gaussian fits of educt (antibody + 1 pep, antibody + 2 peps) and product (peptide, antibody + 0 pep, antibody fragments) ion signals upon gas phase dissociation of anti-Troponin antibody complexed with Troponin I peptide 1.

peptide 1, measurement 1

ion	ΔCV										
	**4**	**8**	**12**	**16**	**20**	**30**	**40**	**50**	**60**	**70**	**80**
**peptide 1**	20.08	119.14	293.63	508.05	871.15	2001.75	3311.92	4630.81	6625.84	7596.81	7863.30
**antibody + 0 pep**	1560.65	3575.42	3758.80	4256.50	4473.02	4439.86	4974.13	5063.43	5422.00	5416.84	5446.53
**antibody + 1 pep**	1759.65	3916.50	4101.82	4618.64	4751.23	4662.70	5000.77	5063.99	5274.46	5150.44	5142.85
**antibody + 2 peps**	503.34	1128.70	1182.03	1319.88	1337.38	1274.14	1357.39	1323.64	1297.37	1255.40	1226.20
**antibody fragments**							84.43	129.34	238.72	386.95	892.07

peptide 1, measurement 2

ion	ΔCV												
	**2**	**4**	**6**	**8**	**12**	**16**	**20**	**30**	**40**	**50**	**60**	**70**	**80**
**peptide 1**	96.45	169.86	341.46	553.15	1127.69	1633.99	2119.96	3329.73	5913.23	7976.70	8716.03	9014.69	8888.83
**antibody + 0 pep**	4665.54	4874.27	6454.31	7084.26	7245.07	8076.08	8040.37	7291.05	8150.62	7928.75	7617.47	7248.19	6968.70
**antibody + 1 pep**	5960.01	6119.97	8196.73	8907.66	8937.94	9772.17	9650.46	8735.05	9185.97	8910.77	8505.97	7898.42	7476.38
**antibody + 2 peps**	3500.50	3725.69	3745.14	3639.83	3664.84	3237.09	3116.27	2760.29	2850.01	2601.03	2419.74	2163.80	2062.89
**antibody fragments**	155.98	171.79	143.63	143.08	144.31	146.45	149.64	183.11	218.19	241.83	348.54	502.61	878.74

peptide 1, measurement 3

ion	ΔCV												
	**2**	**4**	**6**	**8**	**12**	**16**	**20**	**30**	**40**	**50**	**60**	**70**	**80**
**peptide 1**	107.65	175.05	239.32	361.03	1185.51	2082.29	2871.58	5104.50	7176.28	9711.76	11191.91	11049.64	11500.75
**antibody + 0 pep**	3610.91	4106.76	4093.79	3793.54	3666.84	4989.85	4917.34	5237.32	5468.69	5774.38	5816.16	5484.61	5482.18
**antibody + 1 pep**	6369.89	7203.19	7271.43	6881.29	6755.91	8591.76	8401.60	8891.83	9092.09	9159.76	9057.49	8495.29	8388.70
**antibody + 2 peps**	3029.38	3322.47	3395.06	3287.11	3209.51	4041.04	3824.93	3885.75	3930.56	3721.37	3579.89	3323.28	3290.75
**antibody fragments**	163.30	160.88	195.73	193.71	209.48	204.66	211.02	249.91	283.28	320.68	402.37	485.29	817.47

peptide 1, measurement 4

ion	ΔCV												
	**2**	**4**	**6**	**8**	**12**	**16**	**20**	**30**	**40**	**50**	**60**	**70**	**80**
**peptide 1**	79.46	129.86	220.19	331.56	592.87	1126.51	1794.14	3494.39	6229.77	7906.88	10190.94	10785.02	10900.22
**antibody + 0 pep**	2577.39	2084.74	2562.67	3133.06	2126.27	2574.86	3013.99	3299.68	3686.08	3570.43	3983.55	3877.92	3508.22

**antibody + 1 pep**	4990.91	3920.54	4789.27	5935.94	3191.97	4279.29	5041.23	5604.53	6036.31	5795.18	6230.84	5920.06	5260.63
**antibody + 2 peps**	2390.10	2260.39	2502.04	2956.83	2890.77	3082.27	3309.74	3322.14	3234.71	2860.98	2772.84	2582.03	2460.22
**antibody fragments**	146.27	127.62	150.38	169.75	108.82	132.48	149.26	161.50	196.50	224.44	272.53	294.48	381.09

Table S10. continued

peptide 1, measurement 5

**ion**	**ΔCV**												
	**2**	**4**	**6**	**8**	**12**	**16**	**20**	**30**	**40**	**50**	**60**	**70**	**80**
**peptide 1**	59.92	71.24	365.99	500.76	1187.91	2346.90	3548.31	7733.72	14640.64	23804.88	32021.49	35687.87	37275.38
**antibody + 0 pep**	2153.10	5073.03	8253.94	11549.08	12682.79	12146.86	12729.48	12690.04	13795.51	14279.36	15455.43	15720.93	15269.13
**antibody + 1 pep**	4194.19	10020.47	15862.35	21383.36	23344.51	22355.04	23464.29	23114.91	24077.02	24183.56	24835.56	24503.12	23972.25
**antibody + 2 peps**	2130.05	5877.56	8298.35	10661.89	11678.62	10879.49	11300.24	11053.99	11149.52	10553.82	10340.26	10007.60	9432.65
**antibody fragments**				228.56	339.77	388.80	401.94	437.42	662.10	830.47	1016.15	1302.29	2405.83

peptide 1, measurement 6

**ion**	**ΔCV**												
	**2**	**4**	**6**	**8**	**12**	**16**	**20**	**30**	**40**	**50**	**60**	**70**	**80**
**peptide 1**	46.54	83.36	277.92	409.47	770.59	1810.89	2490.51	4835.50	7023.20	12953.05	15995.50	16925.50	23265.63
**antibody + 0 pep**	2204.52	3711.92	6040.69	7467.20	7493.89	7863.29	6952.00	7675.22	6773.48	8463.87	8826.01	8464.64	10398.83
**antibody + 1 pep**	4402.93	7355.58	11753.46	14327.94	14242.10	14720.60	13112.54	14465.88	12472.88	14530.21	14821.98	13758.94	16490.30
**antibody + 2 peps**	2376.03	3906.83	6013.78	7165.67	7083.56	7210.92	6679.75	7119.46	6011.32	6354.74	6248.86	5673.43	6630.92
**antibody fragments**			69.95	283.44	330.04	375.42	330.13	406.57	405.53	537.26	726.44	827.38	2138.44

peptide 1, measurement 7

**ion**	**ΔCV**												
	**2**	**4**	**6**	**8**	**12**	**16**	**20**	**30**	**40**	**50**	**60**	**70**	**80**
**peptide 1**	80.36	121.00	214.07	262.32	611.72	1019.39	1811.31	3889.30	7485.65	10715.76	13510.41	13915.33	15378.52
**antibody + 0 pep**	953.45	1773.11	1910.00	1875.47	2085.37	2148.65	2306.95	2828.24	3482.64	4095.57	4476.08	4291.64	4438.60
**antibody + 1 pep**	2536.28	4637.87	4981.30	4888.84	5406.17	5557.34	5986.57	7113.10	8626.34	9822.22	10248.62	9807.44	10059.00
**antibody + 2 peps**	1883.74	3252.71	3482.68	3387.91	3692.32	3788.77	4135.37	4835.10	5503.40	5916.61	6068.15	5637.94	5712.16
**antibody fragments**									130.36	259.06	371.75	686.85	1733.13

Table S11. Apex heights of Gaussian fits of educt (antibody + 1 pep, antibody + 2 peps) and product (peptide, antibody + 0 pep, antibody fragments) ion signals upon gas phase dissociation of anti-Troponin antibody complexed with Troponin I peptide 2.

peptide 2, measurement 1

ion	ΔCV									
	**4**	**8**	**12**	**16**	**20**	**30**	**40**	**50**	**60**	**70**	**80**
**peptide 2**	67.32	272.96	745.73	1388.48	2384.32	4942.70	7868.58	11421.31	16130.08	16754.08	16559.05
**antibody + 0 pep**	698.69	1414.01	1809.11	1821.20	1913.65	2047.82	2326.51	2628.22	2969.89	3012.22	2972.78
**antibody + 1 pep**	2691.94	5221.40	6974.64	7067.49	7404.73	7546.77	8270.46	8962.36	9745.20	9703.01	9440.08
**antibody + 2 peps**	2741.97	5408.89	7151.47	7204.99	7362.56	7190.17	7441.11	7577.66	7832.33	7433.50	7249.24
**antibody fragments**							111.78	243.59	354.66	625.68	1459.47

peptide 2, measurement 2

ion	ΔCV												
	**2**	**4**	**6**	**8**	**12**	**16**	**20**	**30**	**40**	**50**	**60**	**70**	**80**
**peptide 2**	61.42	85.74	140.29	212.03	539.03	851.38	1250.97	2538.86	4306.85	6296.18	7286.95	7279.45	7211.32
**antibody + 0 pep**	720.28	752.15	765.68	894.10	1208.53	1243.47	1240.94	1242.83	1384.99	1545.23	1611.08	1630.72	1662.12
**antibody + 1 pep**	3135.11	3308.49	3452.27	3889.31	4994.14	4690.22	4898.72	4941.70	5175.84	5450.45	5471.25	5460.67	5404.46
**antibody + 2 peps**	3447.11	3699.43	3736.50	4170.49	5101.90	5084.55	5188.74	5048.74	4962.00	4867.86	4685.46	4485.77	4311.65
**antibody fragments**	189.65	159.37	159.46	193.09	181.72	217.18	222.27	211.30	225.47	259.93	321.61	315.11	544.26

peptide 2, measurement 3

ion	ΔCV												
	**2**	**4**	**6**	**8**	**12**	**16**	**20**	**30**	**40**	**50**	**60**	**70**	**80**
**peptide 2**	75.36	119.78	373.02	683.26	1339.49	2171.34	3585.80	5329.01	7534.08	13267.31	14851.53	13811.11	13274.05
**antibody + 0 pep**	1163.20	2163.44	3149.98	4407.09	4584.88	4375.83	4693.75	4403.64	4091.68	5266.26	5258.53	4826.02	4532.47
**antibody + 1 pep**	3458.89	6614.82	9450.56	13064.70	13581.56	13273.45	13883.91	12759.23	11922.62	14281.29	13906.15	12650.65	11930.38
**antibody + 2 peps**	2867.80	5545.20	7849.59	10225.38	10598.65	10300.09	10615.36	9610.27	9118.95	9870.41	9398.33	8472.70	7748.95
**antibody fragments**				233.22	294.98	310.36	319.06	358.52	508.63	670.21	847.50	1029.82	1809.85

peptide 2, measurement 4

ion	ΔCV

	2	4	6	8	12	16	20	30	40	50	60	70	80
**peptide 2**	118.13	262.60	401.18	645.28	1215.13	2114.87	3188.48	5142.21	7135.78	11011.90	13193.46	14682.41	14409.24
**antibody + 0 pep**	1356.13	2347.45	2621.37	2938.35	3181.45	3238.23	3307.21	3391.51	3344.27	4148.28	4599.86	5046.02	4893.05
**antibody + 1 pep**	4030.57	7013.31	7886.38	8922.16	9165.77	9270.26	9476.87	9301.31	9052.46	10581.18	11341.01	12067.88	11698.66
**antibody + 2 peps**	3166.76	5484.63	6154.35	6942.65	6930.32	6913.07	7043.34	6663.19	6168.61	6844.71	7001.68	7489.76	7044.87
**antibody fragments**									150.50	286.41	333.68	780.56	1729.10

TableS 11. continued

peptide 2, measurement 5

ion	ΔCV												
	**2**	**4**	**6**	**8**	**12**	**16**	**20**	**30**	**40**	**50**	**60**	**70**	**80**
**peptide 2**	107.94	260.65	563.57	747.22	1457.09	2548.74	3867.28	7127.19	10581.41	12944.93	15118.79	15300.87	15535.78
**antibody + 0 pep**	1442.12	2444.43	2840.65	3130.95	3318.49	3554.78	3673.69	4145.85	4423.75	4799.65	5027.63	5096.31	5057.08
**antibody + 1 pep**	4274.93	7272.75	8503.97	9093.16	9796.27	10276.39	10482.53	11232.68	11625.11	12046.25	12331.09	12306.56	12220.36
**antibody + 2 peps**	3353.98	5772.09	6534.41	7077.62	7465.36	7747.91	7685.50	7954.52	7893.10	7718.89	7623.79	7422.97	7325.98
**antibody fragments**									207.87	246.89	334.07	765.22	1761.00

Table S12. Apex heights of Gaussian fits of educt (antibody + 1 pep, antibody + 2 peps) and product (peptide, antibody + 0 pep, antibody fragments) ion signals upon gas phase dissociation of anti-Troponin antibody complexed with Troponin I peptide 3.

peptide 3, measurement 1

ion	ΔCV												
	**2**	**4**	**6**	**8**	**12**	**16**	**20**	**30**	**40**	**50**	**60**	**70**	**80**
**peptide 3**	127.80	210.60	471.46	598.94	763.98	736.50	822.71	1379.36	1787.62	3396.89	3956.39	3982.14	3666.04
**antibody + 0 pep**	5572.53	7819.07	8968.55	9715.42	7514.15	5848.56	4630.12	4150.85	4336.47	6117.24	6443.00	6382.44	5939.89
**antibody + 1 pep**	4784.00	6722.80	7638.97	8115.69	6098.48	4603.77	3658.41	3133.00	3213.90	4456.48	4487.35	4603.09	4139.07
**antibody + 2 peps**	1126.65	1524.28	1810.22	1882.07	1406.97	999.21	781.77	689.62	664.45	862.53	848.91	832.21	758.47
**antibody fragments**									47.33	96.26	133.88	234.90	353.27

peptide 3, measurement 2

**ion**	**ΔCV**												
	**2**	**4**	**6**	**8**	**12**	**16**	**20**	**30**	**40**	**50**	**60**	**70**	**80**
**peptide 3**	40.86	68.14	130.53	179.51	369.97	727.05	1147.18	2021.24	3358.80	4636.93	5165.91	5497.45	5427.44
**antibody + 0 pep**	2154.08	4421.98	4730.43	4912.19	5314.68	5718.53	5956.36	6466.79	7088.81	7905.70	8288.73	8347.55	8373.59
**antibody + 1 pep**	1777.06	3671.93	4020.87	4049.20	4389.40	4585.24	4786.94	4943.51	5425.96	5760.50	5815.67	5898.36	5860.79
**antibody + 2 peps**	434.85	843.62	900.03	898.80	972.63	1050.19	1041.74	1072.43	1112.97	1137.51	1108.60	1091.00	1107.54
**antibody fragments**									82.17	136.60	185.13	270.57	637.54

Table S13. Apex heights of Gaussian fits of educt (antibody + 1 pep, antibody + 2 peps) and product (peptide, antibody + 0 pep, antibody fragments) ion signals upon gas phase dissociation of anti-Troponin antibody complexed with Troponin I peptide 4.

peptide 4, measurement 1

ion	ΔCV										
	**4**	**8**	**12**	**16**	**20**	**30**	**40**	**50**	**60**	**70**	**80**
**peptide 4**	12.35	71.32	275.47	519.89	786.78	1067.65	1427.71	2275.65	3438.57	4377.29	4214.36
**antibody + 0 pep**	689.27	2350.29	3336.85	3677.69	3553.81	2702.78	2703.53	2952.49	3649.82	4317.45	3925.67
**antibody + 1 pep**	481.20	1810.64	2610.34	2800.55	2663.44	2128.39	1900.37	2052.10	2315.07	2651.67	2515.57
**antibody + 2 peps**	99.35	397.24	567.26	619.98	611.17	493.80	392.68	404.33	407.44	462.70	420.41
**antibody fragments**									57.67	187.11	383.41

peptide 4, measurement 2

ion	ΔCV												
	**2**	**4**	**6**	**8**	**12**	**16**	**20**	**30**	**40**	**50**	**60**	**70**	**80**
**peptide 4**	43.85	116.14	348.54	461.60	885.50	1245.42	1704.68	3284.33	5095.10	4444.91	4837.05	6361.56	5310.18
**antibody + 0 pep**	4082.12	5704.96	8663.47	8779.23	8584.55	8058.98	7915.14	9845.72	10846.74	8203.83	9050.89	11352.63	9321.16
**antibody + 1 pep**	2710.41	3796.99	5367.58	5417.28	5214.46	4887.01	4975.12	5943.37	6337.56	4882.06	5166.05	6314.09	5222.44
**antibody + 2 peps**	500.54	680.05	950.63	905.33	864.99	825.93	901.95	955.85	1042.39	812.07	851.29	938.04	813.50
**antibody fragments**		21.26	234.71	462.01	503.62	476.97	547.74	724.05	758.85	744.90	915.57	1402.37	1168.85

peptide 4, measurement 3

ion	ΔCV												
	**2**	**4**	**6**	**8**	**12**	**16**	**20**	**30**	**40**	**50**	**60**	**70**	**80**
**peptide 4**	39.74	101.53	205.50	355.82	698.73	1267.38	1749.89	1962.95	4597.21	5487.23	5613.29	5773.48	5491.09
**antibody + 0 pep**	3221.50	4317.49	5471.52	5725.12	6308.73	7259.11	7385.92	7170.38	8893.43	9726.11	9541.32	9666.13	9406.18
**antibody + 1 pep**	2291.55	3023.79	3696.25	3935.78	4255.45	4827.89	4794.27	4649.74	5476.13	5737.68	5636.00	5692.21	5434.54
**antibody + 2 peps**	426.11	585.95	685.62	756.55	804.47	865.71	886.31	811.02	924.44	952.04	893.76	879.92	866.44
**antibody fragments**		41.83	224.23	399.90	418.18	471.03	577.63	579.57	745.81	839.11	903.54	882.63	1213.13

peptide 4, measurement 4

ion	ΔCV

	2	4	6	8	12	16	20	30	40	50	60	70	80
**peptide 4**	8.81	25.45	53.34	74.12	247.59	502.29	750.10	2352.37	4501.47	6128.32	6595.08	6774.57	7162.90
**antibody + 0 pep**	1467.98	2275.16	3331.92	4299.10	5156.87	6657.81	7202.47	10338.77	14380.17	16317.63	17098.91	17253.87	18696.39
**antibody + 1 pep**	694.89	1080.28	1550.16	1919.91	2240.81	2768.08	2988.82	4011.55	5176.95	5617.03	5711.35	5631.24	5908.71
**antibody + 2 peps**	131.95	209.02	296.38	348.73	374.88	456.51	468.15	537.92	654.67	658.83	617.85	592.17	615.62
**antibody fragments**									123.69	272.75	476.32	747.32	1496.83

TableS 13. continued

peptide 4, measurement 5

**ion**	**ΔCV**												
	**2**	**4**	**6**	**8**	**12**	**16**	**20**	**30**	**40**	**50**	**60**	**70**	**80**
**peptide 4**	77.06	246.54	564.32	809.08	1735.19	3056.89	4368.54	7996.18	11071.47	14368.06	14958.92	14384.83	15056.73
**antibody + 0 pep**	5820.1	9904.2	13066.72	14722.52	17593.71	20580.31	21959.07	24802.77	27558.79	29767.54	29863.47	29275.86	29247.61
**antibody + 1 pep**	2355.0	3899.03	5054.05	5693.11	6534.33	7393.57	7722.78	8113.89	8546.03	8884.76	8525.26	8013.85	7994.48
**antibody + 2 peps**	326.54	542.15	682.13	749.06	877.88	949.86	984.13	1022.67	956.12	1007.27	944.75	840.10	849.14
**antibody fragments**								120.81	283.42	517.38	643.54	1286.32	2759.89

Table S14. Apex heights of Gaussian fits of educt (antibody + 1 pep, antibody + 2 peps) and product (peptide, antibody + 0 pep, antibody fragments) ion signals upon gas phase dissociation of anti-Troponin antibody complexed with Troponin I peptide 5.

peptide 5, measurement 1

**ion**	**ΔCV**												
	**2**	**4**	**6**	**8**	**12**	**16**	**20**	**30**	**40**	**50**	**60**	**70**	**80**
**peptide 5**	173.60	219.33	575.94	587.69	969.99	1532.19	2115.3	4130.77	7429.81	11878.90	12870.10	13025.40	12820.70
**antibody + 0 pep**	6774.08	8138.97	6794.22	8716.95	8712.91	8624.20	7875.23	9182.34	9660.95	10600.30	10547.50	10658.10	10591.70
**antibody + 1 pep**	7774.92	8814.28	7944.37	9301.92	9151.57	8990.23	8528.78	9286.09	9383.04	9829.60	9500.34	9525.96	9160.70
**antibody + 2 peps**	2607.51	2715.78	2960.88	2805.92	2713.30	2614.45	2689.78	2680.87	2625.27	2540.91	2444.44	2340.81	2228.29
**antibody fragments**	152.37	162.16	156.93	169.41	195.33	195.14	201.33	210.55	213.33	155.50	408.77	644.78	746.06

peptide 5, measurement 2

ion	ΔCV												
	**2**	**4**	**6**	**8**	**12**	**16**	**20**	**30**	**40**	**50**	**60**	**70**	**80**
**peptide 5**	274.00	452.86	662.57	1051.60	1965.64	3326.04	4363.99	7449.64	13411.07	20837.00	23602.75	23515.07	22713.11
**antibody + 0 pep**	10628.19	12223.25	12568.23	12283.48	12592.41	13135.95	12658.92	13444.59	14063.35	15101.71	15532.94	14660.18	14180.30
**antibody + 1 pep**	13122.70	13862.76	14002.30	13744.45	14299.13	14423.02	14262.00	14461.96	14795.60	15011.87	15055.22	14305.58	13777.57
**antibody + 2 peps**	4591.07	4472.59	4277.14	4449.99	4565.32	4664.72	4554.31	4550.43	4455.35	4231.48	4149.56	3862.86	3797.89
**antibody fragments**	205.99	194.26	213.23	228.46	263.88	254.81	261.38	324.79	360.69	406.01	425.16	567.47	694.52

Table S14. continued

peptide 5, measurement 3

ion	ΔCV												
	**2**	**4**	**6**	**8**	**12**	**16**	**20**	**30**	**40**	**50**	**60**	**70**	**80**
**peptide 5**	251.26	308.70	553.58	833.65	1586.26	2755.06	4068.58	6756.54	12347.70	19441.40	22465.50	22602.10	22745.90
**antibody + 0 pep**	8233.91	8852.90	9913.13	9868.38	10109.40	11041.50	11641.30	12116.30	12807.00	14181.40	15107.40	15154.40	15429.50
**antibody + 1 pep**	10431	10908.40	11775.10	12005.60	12053.40	12500.90	13011.80	13278.60	13541.90	13931.60	14143.30	14059	14137.30
**antibody + 2 peps**	3754.60	3912.04	4009.24	4188.21	4166.93	4065.86	4107.91	4170.46	4131.57	3851.66	3729.81	3603.88	3625.49
**antibody fragments**	160.19	168.64	178.42	194.78	199.09	200.36	202.00	249.66	315.62	389.36	466.12	576.20	710.33

peptide 5, measurement 4

ion	ΔCV												
	**2**	**4**	**6**	**8**	**12**	**16**	**20**	**30**	**40**	**50**	**60**	**70**	**80**
**peptide 5**	16.47	59.32	158.35	419.73	623.67	1519.09	2668.73	3571.74	5936.14	8307.26	10383.00	11281.20	11709.41
**antibody + 0 pep**	862.83	2401.41	4010.64	5912.64	6965.60	8651.88	9776.39	9804.21	10539.09	11018.25	12046.10	12616.59	12974.22
**antibody + 1 pep**	759.32	1982.27	3279.28	4846.22	5639.33	6875.38	7684.97	7507.48	7800.15	7748.14	8289.69	8377.27	8382.97
**antibody + 2 peps**	178.93	474.09	752.97	1133.43	1319.68	1513.15	1672.59	1626.81	1621.45	1555.63	1540.89	1536.30	1500.03
**antibody fragments**									153.26	247.51	332.24	503.26	1367.15

peptide 5, measurement 5

ion	ΔCV												
	**2**	**4**	**6**	**8**	**12**	**16**	**20**	**30**	**40**	**50**	**60**	**70**	**80**
**peptide 5**	41.34	91.23	329.77	595.19	971.02	1784.46	3016.55	2658.60	2904.47	4995.02	7987.69	11850.28	13306.11
**antibody + 0 pep**	1581.10	3676.32	5790.62	7783.78	8749.01	9760.17	10739.63	8686.45	7466.76	8665.00	10686.65	13434.12	14479.16
**antibody + 1 pep**	1274.03	2931.58	4788.01	6355.17	6973.55	7653.81	8246.83	6656.40	5759.21	6339.64	7504.16	8960.89	9403.25
**antibody + 2 peps**	288.62	676.66	1105.89	1413.21	1572.74	1675.90	1778.90	1462.09	1190.97	1265.90	1402.58	1614.35	1675.85
**antibody fragments**									108.40	185.64	337.77	493.44	1709.46

Table S15. Apex heights of Gaussian fits of educt (antibody + 1 pep, antibody + 2 peps) and product (peptide, antibody + 0 pep, antibody fragments) ion signals upon gas phase dissociation of anti-Troponin antibody complexed with Troponin I peptide 6.

peptide 6, measurement 1

**ion**	**ΔCV**										
	**4**	**8**	**12**	**16**	**20**	**30**	**40**	**50**	**60**	**70**	**80**
**peptide 6**	161.71	363.88	754.95	1429.08	2621.11	4287.09	7028.12	8857.59	13064.58	15271.41	13482.67
**antibody + 0 pep**	2404.74	5143.09	4552.27	5629.39	7617.65	7156.95	7653.22	6759.93	8812.03	9183.83	8890.92
**antibody + 1 pep**	2752.61	5850.26	5815.58	6419.71	7702.72	7053.72	7099.32	6252.03	7288.72	7608.71	7042.60
**antibody + 2 peps**	935.94	1998.53	2111.50	2049.80	2132.50	1951.60	1850.22	1673.96	1594.65	1625.04	1488.90
**antibody fragments**							142.29	182.02	409.28	1075.05	2331.77

peptide 6, measurement 2

**ion**	**ΔCV**										
	**4**	**8**	**12**	**16**	**20**	**30**	**40**	**50**	**60**	**70**	**80**
**peptide 6**	32.59	139.45	317.28	681.44	1130.44	1526.55	4110.76	6296.58	10022.93	9926.29	9897.90
**antibody + 0 pep**	1429.6	3282.31	3340.73	3468.37	3429.15	3745.02	4525.63	4867.97	5215.11	5390.13	5282.73
**antibody + 1 pep**	1498.96	3428.99	3598.41	3826.56	3944.94	3903.71	4236.53	4329.18	4446.62	4506.29	4302.57
**antibody + 2 peps**	633.73	1474.36	1499.41	1612.58	1965.36	1397.88	1192.31	1217.47	1157.82	1116.02	1003.86
**antibody fragments**		64.71	49.42	48.66	59.68	46.59	49.70	105.37	184.18	371.44	623.81

peptide 6, measurement 3

**ion**	**ΔCV**												
	**2**	**4**	**6**	**8**	**12**	**16**	**20**	**30**	**40**	**50**	**60**	**70**	**80**
**peptide 6**	81.61	184.36	440.28	719.96	1714.80	3270.41	5249.41	9217.45	13902.38	28227.48	36243.18	43029.33	40402.45
**antibody + 0 pep**	2846.52	5051.41	6367.78	7747.68	9748.35	11484.79	12175.65	13557.85	15045.71	20475.44	21959.70	21867.54	21486.33
**antibody + 1 pep**	2851.01	5151.50	6336.65	7645.63	9478.69	11046.44	11472.97	12091.59	12691.32	14899.34	15283.39	14649.37	14059.47
**antibody + 2 peps**	777.53	1396.59	1687.42	2088.71	2448.87	2814.97	2849.73	2854.19	2843.57	2953.50	2846.46	2720.04	2496.47
**antibody fragments**									260.73	503.68	695.45	1284.29	2912.22

peptide 6, measurement 4

**ion**	**ΔCV**

	2	4	6	8	12	16	20	30	40	50	60	70	80
**peptide 6**	139.79	363.84	790.27	1107.26	2091.23	3889.96	5793.61	11460.78	17046.97	19315.68	22863.83	24783.70	23669.68
**antibody + 0 pep**	3650.01	5082.74	6842.96	8502.73	9728.51	11267.56	11949.41	13463.75	15501.74	16368.28	17388.78	17264.31	16006.27
**antibody + 1 pep**	3481.40	4777.05	6550.54	7930.91	9012.60	9916.36	10401.21	11228.29	11834.38	12079.90	12040.30	12001.19	10948.70
**antibody + 2 peps**	918.89	1346.66	1750.96	2110.07	2270.20	2483.84	2572.87	2598.82	2486.08	2408.69	2314.95	2172.60	2016.49
**antibody fragments**									232.87	352.02	518.85	932.71	1992.49

Table S16. Apex heights of Gaussian fits of educt (antibody + 1 pep, antibody + 2 peps) and product (peptide, antibody + 0 pep, antibody fragments) ion signals upon gas phase dissociation of anti-Troponin antibody complexed with Troponin I peptide 7.

peptide 7, measurement 1

**ion**	**ΔCV**												
	**2**	**4**	**6**	**8**	**12**	**16**	**20**	**30**	**40**	**50**	**60**	**70**	**80**
**peptide 7**	16.99	49.56	103.40	176.28	266.30	393.96	552.58	792.59	1057.89	1304.28	1357.71	1339.85	1437.95
**antibody + 0 pep**	7095.2	12380.7	16551.63	19823.82	21433.30	22070.35	23205.89	23545.16	24220.71	25871.93	25867.30	26712.96	26201.44
**antibody + 1 pep**	610.02	1065.06	1396.58	1682.05	1691.84	1765.41	1791.45	1749.91	1760.06	1740.46	1769.42	1762.40	1739.84
**antibody + 2 peps**	78.12	144.81	194.36	247.32	263.17	267.77	269.82	277.68	275.29	301.92	287.54	274.62	245.05
**antibody fragments**									184.80	241.73	376.19	736.80	1769.88

peptide 7, measurement 2

**ion**	**ΔCV**												
	**2**	**4**	**6**	**8**	**12**	**16**	**20**	**30**	**40**	**50**	**60**	**70**	**80**
**peptide 7**	48.50	72.33	159.67	217.77	293.98	419.61	714.04	897.91	1307.56	1345.92	1565.66	1770.27	1879.02
**antibody + 0 pep**	8702.3	11268.0	15448.70	17073.10	21349.07	23349.93	25869.88	26524.97	27168.09	27553.58	28365.05	31106.27	31622.05
**antibody + 1 pep**	795.91	968.86	1323.24	1450.15	1766.16	1871.28	2078.52	1989.34	1991.19	1925.86	1926.95	2028.19	2044.45
**antibody + 2 peps**	121.99	152.52	190.79	221.87	257.56	290.72	320.74	341.03	302.44	299.68	324.52	325.20	400.00
**antibody fragments**									193.79	324.57	448.49	1009.33	2534.65

Table S17. Mean charge states of educt ion signals upon gas phase dissociation of anti-Troponin antibody complexed with Troponin I peptide 1.

peptide 1, measurement 1

ion	ΔCV									
	4	8	12	16	20	30	40	60	70	80
antibody + 1 pep	24.18	24.17	24.11	24.17	24.19	24.14	24.12	24.08	24.07	24.07
antibody + 2 peps	24.45	24.43	24.36	24.43	24.42	24.41	24.37	24.37	24.31	24.37

peptide 1, measurement 2

ion	ΔCV												
	2	4	6	8	12	16	20	30	40	50	60	70	80
antibody + 1 pep	25.82	25.80	26.08	26.13	26.11	26.13	26.09	25.90	25.95	25.78	25.67	25.65	25.63
antibody + 2 peps	25.87	25.85	26.13	26.21	26.22	26.30	26.28	26.12	26.16	26.03	25.95	25.94	25.91

peptide 1, measurement 3

ion	ΔCV												
	2	4	6	8	12	16	20	30	40	50	60	70	80
antibody + 1 pep	25.88	25.95	25.89	25.77	25.72	25.95	25.89	25.80	25.72	25.69	25.62	25.60	25.56
antibody + 2 peps	26.08	26.12	26.08	25.96	25.92	26.15	26.10	26.03	25.95	25.93	25.89	25.90	25.86

peptide 1, measurement 4

ion	ΔCV												
	2	4	6	8	12	16	20	30	40	50	60	70	80
antibody + 1 pep	25.72	25.72	25.72	25.71	25.68	25.83	25.87	25.90	25.89	25.70	25.68	25.70	25.66
antibody + 2 peps	25.94	25.94	25.94	25.90	25.82	25.93	25.99	26.03	26.03	25.89	25.88	25.92	25.84

peptide 1, measurement 5

ion	ΔCV												
	2	4	6	8	12	16	20	30	40	50	60	70	80
antibody + 1 pep	24.55	24.47	24.47	24.56	24.59	24.54	24.61	24.56	24.57	24.53	24.49	24.51	24.50
antibody + 2 peps	24.79	24.74	24.75	24.82	24.83	24.79	24.86	24.81	24.85	24.81	24.81	24.84	24.84

peptide 1, measurement 6

ion	ΔCV												
	2	4	6	8	12	16	20	30	40	50	60	70	80
antibody + 1 pep	24.67	24.57	24.67	24.72	24.67	24.67	24.71	24.70	24.60	24.66	24.57	24.55	24.63
antibody + 2 peps	24.90	24.84	24.93	24.97	24.91	24.90	24.94	24.94	24.89	24.92	24.87	24.89	24.97

peptide 1, measurement 7

ion	ΔCV												
	2	4	6	8	12	16	20	30	40	50	60	70	80
antibody + 1 pep	25.38	25.40	25.40	25.34	25.32	25.35	25.32	25.31	25.28	25.26	25.19	25.18	25.19
antibody + 2 peps	25.61	25.69	25.65	25.61	25.61	25.63	25.62	25.61	25.61	25.61	25.61	25.60	25.62

Table S18. Mean charge states of educt ion signals upon gas phase dissociation of anti-Troponin antibody complexed with Troponin I peptide 2.

peptide 2, measurement 1

ion	ΔCV										
	4	8	12	16	20	30	40	50	60	70	80
antibody + 1 pep	24.07	24.17	24.20	24.14	24.19	24.10	24.10	24.11	24.05	23.99	24.01
antibody + 2 peps	24.28	24.39	24.41	24.38	24.43	24.36	24.38	24.40	24.37	24.34	24.35

peptide 2, measurement 2

ion	ΔCV												
	2	4	6	8	12	16	20	30	40	50	60	70	80
antibody + 1 pep	25.99	25.98	25.98	26.06	26.13	26.10	26.07	25.99	25.92	25.82	25.74	25.71	25.70
antibody + 2 peps	26.19	26.18	26.18	26.23	26.33	26.32	26.28	26.27	26.23	26.17	26.11	26.10	26.11

peptide 2, measurement 3

ion	ΔCV												
	2	4	6	8	12	16	20	30	40	50	60	70	80
antibody + 1 pep	24.95	24.94	24.96	25.05	25.08	25.05	25.09	24.96	24.85	24.89	24.80	24.73	24.73
antibody + 2 peps	25.21	25.22	25.24	25.31	25.32	25.31	25.36	25.26	25.19	25.24	25.19	25.12	25.12

peptide 2, measurement 4

ion	ΔCV												
	2	4	6	8	12	16	20	30	40	50	60	70	80
antibody + 1 pep	25.48	25.50	25.52	25.51	25.53	25.50	25.52	25.46	25.38	25.34	25.33	25.32	25.31
antibody + 2 peps	25.78	25.81	25.83	25.82	25.82	25.81	25.83	25.79	25.76	25.75	25.74	25.76	25.76

peptide 2, measurement 5

ion	ΔCV												
	2	4	6	8	12	16	20	30	40	50	60	70	80
antibody + 1 pep	25.45	25.49	25.51	25.51	25.52	25.52	25.52	25.49	25.42	25.37	25.33	25.32	25.31
antibody + 2 peps	25.74	25.79	25.79	25.80	25.81	25.83	25.83	25.83	25.80	25.78	25.76	25.76	25.76

Table S19. Mean charge states of educt ion signals upon gas phase dissociation of anti-Troponin antibody complexed with Troponin I peptide 3.

peptide 3, measurement 1

ion	ΔCV												
	2	4	6	8	12	16	20	30	40	50	60	70	80
antibody + 1 pep	25.26	25.28	25.40	25.34	25.47	25.41	25.43	25.42	25.36	25.29	25.27	25.26	25.21
antibody + 2 peps	25.48	25.49	25.60	25.55	25.64	25.62	25.60	25.59	25.56	25.54	25.45	25.51	25.54

peptide 3, measurement 2

ion	ΔCV												
	2	4	6	8	12	16	20	30	40	50	60	70	80
antibody + 1 pep	25.26	25.28	25.27	25.26	25.28	25.29	25.29	25.26	25.25	25.22	25.19	25.17	25.15
antibody + 2 peps	25.47	25.54	25.53	25.46	25.51	25.50	25.49	25.46	25.48	25.46	25.45	25.42	25.44

Table S20. Mean charge states of educt ion signals upon gas phase dissociation of anti-Troponin antibody complexed with Troponin I peptide 4.

peptide 4, measurement 1

**ion**	**ΔCV**										
	**4**	**8**	**12**	**16**	**20**	**30**	**40**	**50**	**60**	**70**	**80**
**antibody + 1 pep**	24.25	24.18	24.21	24.16	24.11	24.13	24.03	24.05	24.04	24.04	23.98
**antibody + 2 peps**	24.33	24.31	24.29	24.25	24.19	24.25	24.11	24.11	24.11	24.15	24.10

peptide 4, measurement 2

**ion**	**ΔCV**												
	**2**	**4**	**6**	**8**	**12**	**16**	**20**	**30**	**40**	**50**	**60**	**70**	**80**
**antibody + 1 pep**	24.77	24.81	24.89	24.92	24.91	24.90	24.90	24.91	24.91	24.77	24.76	24.79	24.72
**antibody + 2 peps**	24.92	24.90	24.95	24.97	24.99	24.96	24.97	24.97	24.97	24.91	24.94	24.92	24.83

peptide 4, measurement 3

**ion**	**ΔCV**												
	**2**	**4**	**6**	**8**	**12**	**16**	**20**	**30**	**40**	**50**	**60**	**70**	**80**
**antibody + 1 pep**	24.74	24.82	24.85	24.88	24.89	24.93	24.87	24.88	24.84	24.84	24.77	24.79	24.78
**antibody + 2 peps**	24.88	24.95	24.96	24.98	24.99	24.98	24.93	24.97	24.92	24.95	24.89	24.96	24.90

peptide 4, measurement 4

**ion**	**ΔCV**												
	**2**	**4**	**6**	**8**	**12**	**16**	**20**	**30**	**40**	**50**	**60**	**70**	**80**
**antibody + 1 pep**	25.44	25.39	25.45	25.39	25.40	25.39	25.37	25.43	25.45	25.48	25.47	25.47	25.48
**antibody + 2 peps**	25.50	25.43	25.45	25.45	25.44	25.45	25.45	25.43	25.54	25.52	25.49	25.54	25.58

peptide 4, measurement 5

**ion**	**ΔCV**												
	**2**	**4**	**6**	**8**	**12**	**16**	**20**	**30**	**40**	**50**	**60**	**70**	**80**
**antibody + 1 pep**	25.61	25.68	25.70	25.72	25.80	25.86	25.89	25.90	25.90	25.90	25.87	25.86	25.85
**antibody + 2 peps**	25.66	25.81	25.84	25.76	25.94	25.95	25.89	25.91	25.89	25.86	25.87	25.91	25.88

Table S21. Mean charge states of educt ion signals upon gas phase dissociation of anti-Troponin antibody complexed with Troponin I peptide 5.

peptide 5, measurement 1

**ion**	**ΔCV**												
	**2**	**4**	**6**	**8**	**12**	**16**	**20**	**30**	**40**	**50**	**60**	**70**	**80**
**antibody + 1 pep**	25.65	25.66	25.58	25.59	25.53	25.49	25.53	25.55	25.49	25.49	25.43	25.42	25.34
**antibody + 2 peps**	25.75	25.77	25.66	25.71	25.59	25.60	25.65	25.67	25.62	25.66	25.62	25.61	25.54

peptide 5, measurement 2

**ion**	**ΔCV**												
	**2**	**4**	**6**	**8**	**12**	**16**	**20**	**30**	**40**	**50**	**60**	**70**	**80**
**antibody + 1 pep**	25.58	25.59	25.54	25.46	25.47	25.47	25.42	25.42	25.33	25.31	25.23	25.25	25.23
**antibody + 2 peps**	25.66	25.69	25.61	25.57	25.56	25.57	25.54	25.53	25.48	25.49	25.42	25.45	25.44

peptide 5, measurement 3

ion	ΔCV												
	**2**	**4**	**6**	**8**	**12**	**16**	**20**	**30**	**40**	**50**	**60**	**70**	**80**
**antibody + 1 pep**	25.45	25.43	25.48	25.45	25.41	25.51	25.50	25.46	25.39	25.30	25.29	25.28	25.28
**antibody + 2 peps**	25.59	25.55	25.60	25.58	25.53	25.60	25.60	25.58	25.53	25.44	25.48	25.46	25.47

peptide 5, measurement 4

ion	ΔCV												
	**2**	**4**	**6**	**8**	**12**	**16**	**20**	**30**	**40**	**50**	**60**	**70**	**80**
**antibody + 1 pep**	25.50	25.53	25.53	25.52	25.55	25.56	25.58	25.57	25.54	25.54	25.51	25.54	25.52
**antibody + 2 peps**	25.71	25.74	25.74	25.72	25.77	25.75	25.80	25.80	25.78	25.77	25.77	25.79	25.79

peptide 5, measurement 5

ion	ΔCV												
	**2**	**4**	**6**	**8**	**12**	**16**	**20**	**30**	**40**	**50**	**60**	**70**	**80**
**antibody + 1 pep**	25.59	25.59	25.61	25.62	25.62	25.67	25.63	25.52	25.49	25.53	25.55	25.54	25.55
**antibody + 2 peps**	25.85	25.83	25.82	25.85	25.83	25.90	25.80	25.74	25.70	25.77	25.74	25.79	25.83

Table S22. Mean charge states of educt ion signals upon gas phase dissociation of anti-Troponin antibody complexed with Troponin I peptide 6.

peptide 6, measurement 1

ion	ΔCV										
	**4**	**8**	**12**	**16**	**20**	**30**	**40**	**50**	**60**	**70**	**80**
**antibody + 1 pep**	24.47	24.49	24.52	24.59	24.68	24.62	24.63	24.59	24.60	24.61	24.58
**antibody + 2 peps**	24.70	24.67	24.71	24.77	24.85	24.81	24.84	24.81	24.78	24.81	24.81

peptide 6, measurement 2

ion	ΔCV										
	**4**	**8**	**12**	**16**	**20**	**30**	**40**	**50**	**60**	**70**	**80**
**antibody + 1 pep**	25.41	25.42	25.50	25.52	25.49	25.47	25.51	25.46	25.36	25.32	25.28
**antibody + 2 peps**	25.66	25.65	25.74	25.78	25.79	25.74	25.75	25.72	25.64	25.61	25.54

peptide 6, measurement 3

ion	ΔCV												
	**2**	**4**	**6**	**8**	**12**	**16**	**20**	**30**	**40**	**50**	**60**	**70**	**80**
**antibody + 1 pep**	25.44	25.46	25.47	25.47	25.46	25.49	25.50	25.52	25.53	25.82	25.86	25.96	25.91
**antibody + 2 peps**	25.69	25.69	25.73	25.68	25.72	25.75	25.75	25.77	25.76	26.04	26.10	26.19	26.15

peptide 6, measurement 4

ion	ΔCV												
	**2**	**4**	**6**	**8**	**12**	**16**	**20**	**30**	**40**	**50**	**60**	**70**	**80**
**antibody + 1 pep**	25.83	25.82	25.81	25.86	25.85	25.93	25.94	25.95	25.95	25.89	25.91	25.90	25.83
**antibody + 2 peps**	26.09	26.10	26.03	26.08	26.10	26.14	26.18	26.20	26.19	26.17	26.11	26.15	26.11

TableS 23. Mean charge states of educt ion signals upon gas phase dissociation of anti-Troponin antibody complexed with Troponin I peptide 7.

peptide 7, measurement 1

ion	ΔCV												
	**2**	**4**	**6**	**8**	**12**	**16**	**20**	**30**	**40**	**50**	**60**	**70**	**80**

**antibody + 1 pep**	26.02	26.09	26.10	26.18	26.17	26.16	26.16	26.18	26.12	26.11	26.08	26.08	26.04
**antibody + 2 peps**	26.00	26.11	26.09	26.12	26.09	26.04	26.25	26.08	25.97	25.99	25.80	26.48	26.29

peptide 7, measurement 2

**ion**	**ΔCV**												
	**2**	**4**	**6**	**8**	**12**	**16**	**20**	**30**	**40**	**50**	**60**	**70**	**80**
**antibody + 1 pep**	26.17	26.10	26.08	26.10	26.15	26.19	26.20	26.14	26.15	26.10	26.12	26.09	26.11
**antibody + 2 peps**	26.28	26.20	26.00	25.95	26.30	26.26	26.25	26.20	25.94	25.91	25.93	26.34	n.d.

Table S24. Secondary structure category distribution of amino acid residues of hcTn I epitope peptides over the entire 50 ns simulation time.

peptide no.	amino acid sequence [b]	*secondary structure category* [a]									
		coil	β-sheet	β-bridge	bend	turn	α-helix	π-helix	$^{3}/_{10}$-helix	helical content	
**1 (wt)**	ENREVGDWRKNIDAL	36371	0	0	3368	12925	94726	5	2605	97336	(65%)
**2 (R186Q)**	ENQEVGDWRKNIDAL	38617	0	0	4570	16335	88447	5	2026	90478	(60%)
**3 (R192H)**	ENREVGDWHKNIDAL	54692	0	0	24741	17600	29252	0	23715	52967	(35%)
**4 (R192L)**	ENREVGDWLKNIDAL	54017	0	0	5038	15453	71559	180	3753	75492	(50%)
**5 (R192C)**	ENREVGDWCKNIDAL	60712	0	11	21860	27114	17158	661	22484	40303	(27%)
**6 (D190G)**	ENREVGGWRKNIDAL	70970	28	515	26646	31385	17911	0	2545	20456	(14%)
**7 (R192P)**	ENREVGDWPKNIDAL	69375	0	657	35179	18037	17918	0	8834	26752	(18%)
**8 (R192P) (K193E)**	ENREVGDWPENIDAL	49985	0	0	18306	37204	22674	1674	20157	44505	(30%)

a) color coding as in Figure 5 and in Suppl. Figure 21

b) aa184-aa198 from Tn I (UniProt: P19429); the epitope region of the monoclonal anti-hcTn I antibody is underlined; amino acid exchanges in peptides 2-8 are printed in color

Table S25. Secondary structure category distribution of amino acid residues of hcTn I epitope peptides over the first 25 ns simulation time.

peptide no.	amino acid sequence [b]	*secondary structure category* [a]									
		coil	β-sheet	β-bridge	bend	turn	α-helix	π-helix	$^{3}/_{10}$-helix	helical content	
**1 (wt)**	ENREVGDWRKNIDAL	16889	0	0	198	6898	49750	5	1260	51015	(68%)
**2 (R186Q)**	ENQEVGDWRKNIDAL	19611	0	0	3450	8666	42700	0	573	43273	(58%)
**3 (R192H)**	ENREVGDWHKNIDAL	27633	0	0	6245	9816	23645	0	7661	31306	(42%)
**4 (R192L)**	ENREVGDWLKNIDAL	27514	0	0	3403	7106	34994	0	1983	36977	(49%)
**5 (R192C)**	ENREVGDWCKNIDAL	21198	0	5	10264	15993	15970	661	10909	27540	(37%)
**6 (D190G)**	ENREVGGWRKNIDAL	30462	28	513	10680	14423	16489	0	2405	18894	(25%)
**7 (R192P)**	ENREVGDWPKNIDAL	30224	0	42	11817	10809	17874	0	4234	22108	(29%)
**8 (R192P) (K193E)**	ENREVGDWPENIDAL	22543	0	0	8273	18331	15581	1674	8598	25853	(34%)

a) color coding as in Figure 5 and in Suppl. Figure 21

b) aa184-aa198 from Tn I (UniProt: P19429); the epitope region of the monoclonal anti-hcTn I antibody is underlined; amino acid exchanges in peptides 2-8 are printed in color

Table S26. Secondary structure category distribution of amino acid residues of hcTn I epitope peptides over the last 25 ns simulation time.

peptide no.	amino acid sequence [b]	*secondary structure category* [a]									
		coil	β-sheet	β-bridge	bend	turn	α-helix	π-helix	$^{3}/_{10}$-helix	helical content	
**1 (wt)**	ENREVGDWRKNIDAL	19470	0	0	3170	6027	44985		1348	46333	(62%)
**2 (R186Q)**	ENQEVGDWRKNIDAL	18996	0	0	1120	7669	45754	5	1456	47215	(63%)
**3 (R192H)**	ENREVGDWHKNIDAL	27048	0	0	18501	7785	5607		16059	21666	(29%)
**4 (R192L)**	ENREVGDWLKNIDAL	26493	0	0	1635	8350	36572	180	1770	38522	(51%)
**5 (R192C)**	ENREVGDWCKNIDAL	39507	0	6	11597	11124	1188		11578	12766	(17%)
**6 (D190G)**	ENREVGGWRKNIDAL	40499	0	2	15970	16963	1426		140	1566	(2%)
**7 (R192P)**	ENREVGDWPKNIDAL	39142	0	615	23368	7228	44		4603	4647	(6%)

8 (R192P) (K193E)	ENREVGDWPENIDAL	27432	0	0	10034	18882	7093		11559	18652	(25%)

a) color coding as in Figure 5 and in Suppl. Figure 21

b) aa184-aa198 from Tn I (UniProt: P19429); the epitope region of the monoclonal anti-hcTn I antibody is underlined; amino acid exchanges in peptides 2-8 are printed in color

TableS 27. Protonation states, solvated charges, and pI values of hcTn I epitope peptides.

peptide no.	amino acid sequence a)	pI b)	solvated charge	charge positions
1	ENREVGDWRKNIDAL	4.6	-1	$E^{0(-1+1)}NR^{+1}E^{-1}VGD^{-1}WR^{+1}K^{+1}NID^{-1}AL^{-1}$
2	EN**Q**EVGDWRKNIDAL	4.1	-2	$E^{0(-1+1)}NQE^{-1}VGD^{-1}WR^{+1}K^{+1}NID^{-1}AL^{-1}$
3	ENREVGDW**H**KNIDAL	4.6	-1	$E^{0(-1+1)}NR^{+1}E^{-1}VGD^{-1}WH^{+1}K^{+1}NID^{-1}AL^{-1}$
4	ENREVGDW**L**KNIDAL	4.1	-2	$E^{0(-1+1)}NR^{+1}E^{-1}VGD^{-1}WLK^{+1}NID^{-1}AL^{-1}$
5	ENREVGDW**C**KNIDAL	4.1	-2	$E^{0(-1+1)}NR^{+1}E^{-1}VGD^{-1}WR^{+1}K^{+1}NID^{-1}AL^{-1}$
6	ENREVG**G**WRKNIDAL	7.1	0	$E^{0(-1+1)}NR^{+1}E^{-1}VGGWR^{+1}K^{+1}NID^{-1}AL^{-1}$
7	ENREVGDW**P**KNIDAL	4.1	-2	$E^{0(-1+1)}NR^{+1}E^{-1}VGD^{-1}WPK^{+1}NID^{-1}AL^{-1}$
8	ENREVGDW**PE**NIDAL	3.5	-4	$E^{0(-1+1)}NR^{+1}E^{-1}VGD^{-1}WPE^{-1}NID^{-1}AL^{-1}$

a) aa184-aa198 from Tn I (UniProt: P19429); the epitope region of the monoclonal anti-hcTroponin I antibody (clone MF4, ab38210 from abcam) is underlined; amino acid exchanges in peptides 2-8 are printed in bold and are colored

b) https://www.ncbi.nlm.nih.gov/clinvar/; n.a.: not applicable

Table S28. Solvent accessible surface areas (SASAs) of single amino acids of hcTn I epitope peptide 1.

residue No.	residue	SASA before sim. a)	SASA after sim. b)	SASA difference c)
1	GLU	162.38	217.03	54.65
2	ASN	70.39	44.04	-26.35
3	ARG	154.37	218.09	63.72
4	GLU	105.48	128.51	23.03
5	VAL	93.09	81.89	-11.20
6	GLY	1.11	46.75	45.64
7	ASP	69.96	64.60	-5.36
8	TRP	155.60	124.97	-30.63
9	ARG	98.86	149.53	50.67
10	LYS	76.07	112.99	36.92
11	ASN	93.97	85.10	-8.87
12	ILE	98.07	113.10	15.03
13	ASP	96.79	104.67	7.88
14	ALA	85.96	47.72	-38.24
15	LEU	184.75	237.82	53.07

a) SASA calculation with initial structure model of the peptide

b) SASA calculation after atomistic molecular dynamics simulations for 50 ns

c) Difference of SASA values: (SASA after sim. – SASA before sim.). Values are color coded: -20-20: white, 20-60: light green, >60: green, -20 - -60: light red, <-60: red

Table S29. Solvent accessible surface areas (SASAs) of single amino acids of hcTn I epitope peptide 2.

residue No. a)	residue a)	SASA before sim. b)	SASA after sim. c)	SASA difference d)
1	GLU	164.69	170.81	6.12
2	ASN	155.84	101.81	-54.03
**3**	**GLN**	138.74	164.72	25.98
4	GLU	106.48	144.99	38.51
5	VAL	102.83	50.32	-52.51
6	GLY	45.44	39.24	-6.20
7	ASP	90.57	103.56	12.99
8	TRP	158.32	139.37	-18.95
9	ARG	127.77	127.27	-0.50
10	LYS	137.50	132.14	-5.36
11	ASN	86.69	72.31	-14.38
12	ILE	85.32	94.78	9.46
13	ASP	97.34	119.57	22.23
14	ALA	83.27	65.02	-18.25
15	LEU	190.21	182.41	-7.80

a) amino acid exchange is printed in bold and is colored
b) SASA calculation with initial structure model of the peptide
c) SASA calculation after atomistic molecular dynamics simulations for 50 ns
d) Difference of SASA values: (SASA after sim. – SASA before sim.). Values are color coded: -20-20: white, 20-60: light green, >60: green, -20 - -60: light red, <-60: red

Table S30. Solvent accessible surface areas (SASAs) of single amino acids of hcTn I epitope peptide 3.

residue No. a)	residue a)	SASA before sim. b)	SASA after sim. c)	SASA difference d)
1	GLU	173.68	109.46	-64.22
2	ASN	73.76	28.76	-45.00
3	ARG	175.83	167.60	-8.23
4	GLU	119.09	105.92	-13.17
5	VAL	72.96	154.84	81.88
6	GLY	4.96	16.47	11.51
7	ASP	92.66	124.49	31.83
8	TRP	148.91	158.22	9.31
**9**	**HIS**	92.26	28.54	-63.72
10	LYS	108.60	121.18	12.58
11	ASN	85.90	128.53	42.63
12	ILE	81.90	130.12	48.22
13	ASP	129.92	47.44	-82.48
14	ALA	83.78	59.40	-24.38
15	LEU	191.78	180.43	-11.35

a) amino acid exchange is printed in bold and is colored
b) SASA calculation with initial structure model of the peptide
c) SASA calculation after atomistic molecular dynamics simulations for 50 ns

d) Difference of SASA values: (SASA after sim. – SASA before sim.). Values are color coded: -20-20: white, 20-60: light green, >60: green, -20 - -60: light red, <-60: red

Table S31. Solvent accessible surface areas (SASAs) of single amino acids of hcTn I epitope peptide 4.

residue No. a)	residue a)	SASA before sim. b)	SASA after sim. c)	SASA difference d)
1	GLU	167.14	223.72	56.58
2	ASN	135.63	136.48	0.85
3	ARG	196.24	123.53	-72.71
4	GLU	149.40	134.55	-14.85
5	VAL	96.21	102.19	5.98
6	GLY	32.38	41.82	9.44
7	ASP	65.54	81.00	15.46
8	TRP	156.94	142.82	-14.12
**9**	**LEU**	87.69	113.93	26.24
10	LYS	97.62	136.94	39.32
11	ASN	79.67	59.92	-19.75
12	ILE	94.69	121.04	26.35
13	ASP	113.51	127.57	14.06
14	ALA	84.18	63.27	-20.91
15	LEU	193.16	190.31	-2.85

a) amino acid exchange is printed in bold and is colored
b) SASA calculation with initial structure model of the peptide
c) SASA calculation after atomistic molecular dynamics simulations for 50 ns
d) Difference of SASA values: (SASA after sim. – SASA before sim.). Values are color coded: -20-20: white, 20-60: light green, >60: green, -20 - -60: light red, <-60: red

Table S32. Solvent accessible surface areas (SASAs) of single amino acids of hcTn I epitope peptide 5.

residue No. a)	residue a)	SASA before sim. b)	SASA after sim. c)	SASA difference d)
1	GLU	170.27	144.76	-25.51
2	ASN	79.67	117.54	37.87
3	ARG	155.75	166.74	10.99
4	GLU	144.80	46.33	-98.47
5	VAL	83.27	121.59	38.32
6	GLY	7.77	13.75	5.98
7	ASP	50.97	149.08	98.11
8	TRP	150.74	189.46	38.72
**9**	**CYS**	49.44	62.35	12.91
10	LYS	109.33	157.37	48.04
11	ASN	97.69	26.67	-71.02
12	ILE	84.91	144.14	59.23
13	ASP	127.96	129.62	1.66
14	ALA	82.83	49.88	-32.95
15	LEU	188.45	231.85	43.40

a) amino acid exchange is printed in bold and is colored
b) SASA calculation with initial structure model of the peptide

c) SASA calculation after atomistic molecular dynamics simulations for 50 ns
d) Difference of SASA values: (SASA after sim. – SASA before sim.). Values are color coded: -20-20: white, 20-60: light green, >60: green, -20 - -60: light red, <-60: red

Table S33. Solvent accessible surface areas (SASAs) of single amino acids of hcTn I epitope peptide 6.

residue No. [a)]	residue [a)]	SASA before sim. [b)]	SASA after sim. [c)]	SASA difference [d)]
1	GLU	143.73	82.56	-61.17
2	ASN	156.59	147.80	-8.79
3	ARG	232.83	150.22	-82.61
4	GLU	59.70	51.38	-8.32
5	VAL	131.87	147.51	15.64
6	GLY	84.30	91.72	7.42
**7**	**GLY**	15.08	37.88	22.80
8	TRP	162.79	94.09	-68.70
9	ARG	114.11	131.33	17.22
10	LYS	100.14	129.56	29.42
11	ASN	94.15	132.62	38.47
12	ILE	78.04	122.89	44.85
13	ASP	105.63	36.26	-69.37
14	ALA	88.91	67.74	-21.17
15	LEU	183.26	148.94	-34.32

a) Amino acid exchange is printed in bold and is colored
b) SASA calculation with initial structure model of the peptide
c) SASA calculation after atomistic molecular dynamics simulations for 50 ns
d) Difference of SASA values: (SASA after sim. – SASA before sim.). Values are color coded: -20-20: white, 20-60: light green, >60: green, -20 - -60: light red, <-60: red

Table S34. Solvent accessible surface areas (SASAs) of single amino acids of hcTn I epitope peptide 7.

residue No. [a)]	residue [a)]	SASA before sim. [b)]	SASA after sim. [c)]	SASA difference [d)]
1	GLU	128.43	215.13	86.70
2	ASN	117.69	155.28	37.59
3	ARG	103.29	89.65	-13.64
4	GLU	144.53	178.70	34.17
5	VAL	116.45	62.04	-54.41
6	GLY	67.00	33.24	-33.76
7	ASP	105.23	165.56	60.33
8	TRP	143.31	192.29	48.98
**9**	**PRO**	2.25	46.05	43.80
10	LYS	81.56	198.91	117.35
11	ASN	88.39	88.78	0.39
12	ILE	94.53	159.11	64.58
13	ASP	75.54	117.57	42.03
14	ALA	84.54	45.49	-39.05

15	LEU	181.13	211.12	29.99

a) Amino acid exchange is printed in bold and is colored
b) SASA calculation with initial structure model of the peptide
c) SASA calculation after atomistic molecular dynamics simulations for 50 ns
d) Difference of SASA values: (SASA after sim. – SASA before sim.). Values are color coded: -20-20: white, 20-60: light green, >60: green, -20 - -60: light red, <-60: red

Table S35. Solvent accessible surface areas (SASAs) of single amino acids of hcTn I epitope peptide 8.

residue No. a)	residue a)	SASA before sim. b)	SASA after sim. c)	SASA difference d)
1	GLU	163.95	179.78	15.83
2	ASN	119.03	93.09	-25.94
3	ARG	159.85	168.74	8.89
4	GLU	111.33	133.77	22.44
5	VAL	135.54	103.29	-32.25
6	GLY	67.89	57.85	-10.04
7	ASP	33.56	70.86	37.30
8	TRP	165.01	122.01	-43.00
**9**	**PRO**	63.89	51.29	-12.60
**10**	**GLU**	135.88	126.05	-9.83
11	ASN	33.92	116.56	82.64
12	ILE	95.47	126.00	30.53
13	ASP	131.91	121.00	-10.91
14	ALA	84.66	85.45	0.79
15	LEU	177.04	231.06	54.02

a) Amino acid exchange is printed in bold and is colored
b) SASA calculation with initial structure model of the peptide
c) SASA calculation after atomistic molecular dynamics simulations for 50 ns
d) Difference of SASA values: (SASA after sim. – SASA before sim.). Values are color coded: -20-20: white, 20-60: light green, >60: green, -20 - -60: light red, <-60: red
